# Engaging experts and patients to refine the nutrition literacy assessment instrument

**DOI:** 10.1186/s40795-017-0190-y

**Published:** 2017-08-22

**Authors:** Heather D. Gibbs, Susan Harvey, Sarah Owens, Diane Boyle, Debra K. Sullivan

**Affiliations:** 1 Department of Dietetics & Nutrition, University of Kansas Medical Center, Mail Stop 4013, Kansas City, KS 66160 USA; 2 Department of Health, Sport, and Exercise Sciences, University of Kansas, Lawrence, KS USA; 3 Fay W. Whitney School of Nursing, College of Health Sciences, University of Wyoming, Laramie, WY USA

**Keywords:** Health literacy, Patient education, Chronic disease, Nutrition literacy, Portion size, Nutrition education, Surveys and questionnaires

## Abstract

**Background:**

An objective measure of nutrition literacy is unavailable for use in the primary care population. The Nutrition Literacy Assessment instrument (NLit) is a tool designed to measure nutrition literacy across six domains and has been previously piloted in breast cancer and parent populations. The purpose of this research was to engage nutrition experts and patients to guide revisions of the NLit for use in adult primary care.

**Methods:**

Experts (*n* = 5) reviewed each item in the NLit using a survey to assign rankings of their agreement according to relevance, clarity, and reading difficulty. Relevance rankings were used to calculate Scale Content Validity Index. After suggested revisions were made, patients (*n* = 12) were recruited from urban primary care clinics of a University Medical Center located in the Midwestern United States and were interviewed by trained researchers using the cognitive interview approach to generate thoughts, feelings, and ideas regarding NLit items. Data analysis involved qualitative and quantitative methods.

**Results:**

Content validity from expert review was confirmed with a total Scale Content Validity Index of 0.90. Themes emerging from the cognitive interviews resulted in changes in the NLit to improve instrument clarity.

**Conclusion:**

These data suggest the NLit achieves its target constructs, is understood by the target audience, and is ready to undergo validity and reliability testing within the primary care population.

**Electronic supplementary material:**

The online version of this article (doi:10.1186/s40795-017-0190-y) contains supplementary material, which is available to authorized users.

## Background

Health Literacy, or “the degree to which individuals have the capacity to obtain, process, and understand basic health information and services needed to make appropriate health decisions” [1] is a skill fundamental to an individual’s ability to make positive health choices. Landmark studies revealing a high prevalence of low health literacy [2] as well as relationships between low health literacy and poor health outcomes [1] led to development of the U.S. Department of Health and Human Service’s National Action Plan to Improve Health Literacy [3]. This report included seven goals and suggested strategies to achieve a health literate society.

Nutrition education research has produced little evidence base to inform best practices for improving health literacy with regard to nutrition information, despite the critical importance of nutrition for preventing and treating chronic disease. Nutrition is a major underlying factor in both the development and treatment of diabetes [4], hypertension [5], hyperlipidemia [6], and obesity [7]. However, there is some evidence that consumers are confused with regard to nutrition concepts. For example, in one survey of consumers, 52% said “it is easier to do their taxes than figure out how to eat healthfully” [8]. The Nutrition Facts Panel on a food label provides detailed nutrient information and can assist with making nutritious choices, yet increasing evidence demonstrates that most people struggle to apply information found on food labels [9, 10] and those with low health literacy and/or numeracy have greater difficulty [11–13].

The ability of individuals to navigate nutrition-related information is a critical component of nutrition literacy. However, health literacy assessments lack a nutrition focus and generally only identify print literacy and/or numeracy in the context of health care [14]. Although these assessments [15–18] may assist health professionals in determining appropriate reading levels, they do not adequately assess the individual’s proficiency with decisions involving food and/or nutrition. Thus, it is not possible for educators, clinicians, or researchers to identify low nutrition literacy or document improvements in nutrition literacy in the absence of a validated assessment tool.

We took several steps in an effort to address this gap in instrumentation. In previously reported work, we developed an instrument specific to nutrition literacy, based upon input from experienced nutrition educators and registered dietitians [19], pilot-tested the initial design in a small sample of patients in nutrition clinics, and invited critique of instrument methodology from registered dietitians online [14]. Modifications of the instrument were subsequently pilot-tested in two distinct populations separately, including breast cancer patients [20] and parents [21]. These modified instruments were mostly similar to the NLit discussed here but were altered to reflect the nutritional needs of these differing audiences.

The purpose of this study was to build upon our previous work by revising the Nutrition Literacy Assessment Instrument (NLit) for use in the primary care setting with patients who have nutrition-related chronic disease. To this end, we tested the hypothesis that the revised instrument (NLit) is content valid and clearly understood by the general healthcare population.

## Methods

We engaged two audiences to guide revisions of the NLit: 1) nutrition experts, and 2) primary care patients. The Human Subjects Committee of the Institutional Review Board approved all methods for this study with expedited review (HSC# 13805). All data were collected between August 2014 and December 2014.

### The nutrition literacy assessment instrument

**Fig. 1:**
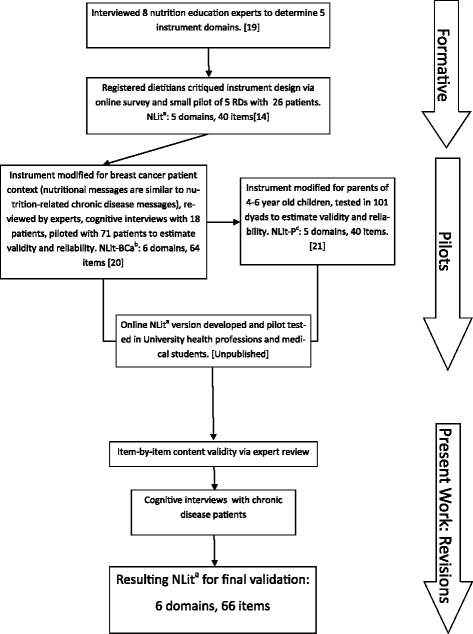
Development of the Nutrition Literacy Assessment Instrument (NLit) ^a^. ^b^ Nutrition Literacy Assessment Instrument in Breast Cancer; ^c^ Nutrition Literacy Assessment Instrument in Parents

The NLit includes measures of print literacy and numeracy, similar to health literacy assessments, while also including measures of nutrition knowledge and skills that nutrition educators identified were needed for following a healthy diet. Prior to content review, the NLit was expanded from five domains totaling 40 items [14] to six domains totaling 71 items in order to ensure internal consistency of the final instrument [22]. The ‘Nutrition & Health’ domain is comprised of a section of prose text summarizing the Dietary Guidelines for Americans written at the 6th grade reading level, followed by fill-in-the-bank-style questions about the reading with answer options in multiple-choice format, also known as the cloze procedure for testing reading comprehension [23]. The ‘Energy Sources in Food’ domain presents questions in multiple-choice format that measure one’s prior knowledge of carbohydrate, protein, and fat sources in food. Questions in the ‘Household Food Measurement’ domain present a photograph of a portion of food, and the amount pictured is provided in the question. Respondents are asked to identify whether the portion is a recommended portion. The term “portion” was used due to previous cognitive interviewing with breast cancer survivors finding that “servings” and “portions” are synonymous terms [20]. The ‘Food Label and Numeracy’ domain presents the United States Food and Drug Administration’s (FDA) food label graphic (which is identified as a label from a package of macaroni and cheese in the NLit), with questions that require reference of the nutrition facts panel in order to choose from the multiple-choice style answers. The ‘Food Groups’ domain requires the ability to classify foods by nutritional category with correct answers in accordance with the food groups as portrayed by the United States Department of Agriculture’s (USDA) MyPlate [24] as well as the American Diabetes Association’s Exchange System [25] for meal planning. Finally, the ‘Consumer Skills’ domain measures the respondent’s ability to navigate food and nutrition products and marketing in order to make nutritious choices. Figure 1 illustrates the sequence and developmental process of the NLit.

#### Expert content review

Experts were recruited based upon their published expertise in survey development (*n* = 1) and current or recent experience with nutrition education (*n* = 4). The four content experts, who are published academics with clinical nutrition experience, evaluated each item in the expanded pool for relevance to the content domain, clarity, and reading difficulty using a survey to assign rankings of their agreement [26]. The remaining expert reviewed the instrument from a psychometric perspective and did not complete the survey of item rankings. Item Content Validity Index (I-CVI), or the content validity of individual items [27], was calculated based upon the combined relevance score for each item in the following fashion: 1) Experts ranked items on a 4-point scale ranging from 1 to 4 with ‘4’ being the most relevant, 2) ‘1–2’ rankings were assigned a score of ‘0’ = ‘not relevant’, and ‘3–4’ rankings were assigned a score of ‘1’ = ‘relevant’. An acceptable I-CVI was set a priori at 0.75 (three of four experts scored the item as relevant). Scale Content Validity Index (S-CVI), or “the average proportion of items given a rating of 3 or 4” [27] was calculated for each domain and for the instrument overall by averaging the I-CVIs. Acceptable S-CVI was set at ≥0.90. Expert rankings of item clarity and additional comments made by all experts were used together to identify the need for item modifications and themes for suggested modifications.

#### Cognitive interviewing

After modifications to the NLit were made resulting from expert review, the instrument was tested using the cognitive interviewing technique [28] with primary care patients. Cognitive interviewing involves an open conversation about mental processes and interpretations on the part of the participant as s/he answers instrument items [29]. The interviews allow the research team to identify problems such as interpretation, decision processes, response selection, as well as problems with instructions and organization.

### Selection of participants

To improve generalizability in the final instrument [30], an intentional sampling approach was used to recruit 12 participants to achieve representation of local ethnicity and race estimates, or approximately 60% Caucasian, 30% African American, and 20% Hispanic. It was also important that all four chronic diseases of interest were represented including diabetes, hyperlipidemia, hypertension, and overweight/obesity. Participants were recruited through providers in a primary practice clinic located in the urban University hospital as well as a registry of patients from the University’s multidisciplinary clinics who have agreed to be contacted for research purposes. Participants received $25 for completed interviews.

Eligibility criteria included age ≥ 18 years and with one or more of the following chronic diseases: diabetes, hyperlipidemia, hypertension, and/or overweight/obesity. We restricted our sample to these chronic diseases because they have strong relationship to dietary treatment of disease. Individuals were ineligible if any of the following criteria were present: overt psychiatric illness, did not speak English, visual acuity insufficient to read the testing instrument, or cognitive impairment.

### Methods and measurement tools

After written informed consent, participants were asked to complete a short demographic survey via REDCap, an electronic data capture tool [31]. They were then prompted to “think aloud” about their thoughts, feelings, and ideas for each instrument item and response option using verbal probing techniques [28]. For example, one commonly used probe researchers would use to check for passage understanding was “How would you explain these sentences to your [mother/friend/neighbor]?” In an effort to increase the usability of the final instrument, the research team chose to develop the NLit into an online format via REDCap. The online version was used in the cognitive interviews to determine ease of use with the target population. Feedback from content experts informed the researchers of potential clarity concerns that required further investigation during cognitive interviews. It should also be noted that a similar version of the NLit was previously tested via cognitive interviewing of 18 breast cancer patients with subsequent pilot testing [20]. The revisions determined from the pilot were incorporated into the present NLit along with minor modifications for the general chronic disease population and feedback from content experts. In order to avoid participant fatigue, interviews did not exceed 60 min. Because the survey was quite long, this consideration for time and fatigue meant that none of the participants were interviewed on the entire instrument. Consequently, researchers prioritized interviews to focus on domains with lower S-CVIs. As a result, the number of participants interviewed about each domain varied, with the Household Food Measurement domain receiving the most attention. Interviews were conducted by two trained research staff and were audio-recorded and transcribed verbatim. Transcription was performed by one author and resulting transcriptions were checked for accuracy by a second author.

### Data analysis

Data analysis included both quantitative and qualitative methods. Frequencies of incorrect answers or difficulties with questions were tabulated. Qualitative data (cognitive interview transcripts, field notes) were analyzed using the constant comparative method [32] and data triangulation [33] to identify patterns of problems and recurrent themes. One member of the research team with over 8 years of qualitative experience led the analysis, while a second member of the research team, also with qualitative experience, assisted in the analysis. Both researchers independently coded the transcripts by hand and then met to discuss the analysis and come to a consensus for recommendations for instrument edits.

## Results

### Content review

For all domains included, the initial S-CVI of the NLit combined was 0.881. After deletion of items with CVI ≤ 0.50, the total S-CVI was 0.90, meeting the target set a priori. Individual domain S-CVIs resulting from reviewer comments are provided in Table 1. Based upon reviewer feedback, four items were deleted while 22 modifications to items were made with slight changes to question wording or answer option wording. For example, in the Consumer Skills domain, the original answer options required a choice between two food items assigned either “A” or “B” while a third option, “C” could be chosen if “A and B are equal in nutrition.” Reviewer feedback identified that “all of the ‘C’ responses should be spelled out, such as ‘Applesauce with no added sugar is equal to an apple in nutrition.’” Other modifications were made to instructional text and formatting. The prose text of the Nutrition & Health domain underwent significant modification to more closely align the questions with the text. For example, several questions in this domain require understanding and application of the terms ‘nutrient density’ and ‘energy density’, terms that are used in the US Dietary Guidelines, so these terms were bolded and given more explanation and food examples for greater clarity. The intention of these textual changes was to ensure that the reader does not need to have prior nutrition knowledge, but rather reading comprehension skills, in order to find correct answers from the text and choose accordingly.

**Table 1:** Summary of Results of Content Expert Review (*n* = 4)

NLit Domain	Scale Content Validity Index (initial)	Scale Content Validity Index (after deletion of items with I-CVI ^a^ ≤ 0.50	Resulting Changes
Nutrition & Health	0.77	0.80	Prose text modified, 1 question deleted, 1 question modified
Energy Sources in Food	0.875	0.91	Definition in instructions modified, 1 question deleted, 2 questions modified
Household Food Measurement	0.72	0.75	1 question deleted
Food Label & Numeracy	0.98	0.98	Instructions modified, “this food” replaced with “macaroni and cheese” throughout questions
Food Groups	0.92	0.92	Food category added, 1 item deleted, 2 items modified, 2 items added
Consumer Skills	0.93	0.93	Organizational structure of answers modified, added “If [calories or portions] are equal…” to 4 items, replaced “claim” with “package states” on 3 items; Spelled out option C for all (i.e. “Applesauce with no added sugar is equal to an apply in nutrition”)

^a^ I-CVI = Item Content Validity Index

### Cognitive interviews

The 12 participants in the cognitive interviews were 59.2 ± 14.3 years old and were more often female (7/12), Caucasian (8/12), and completed some college (11/12). More participants (7/12) reported annual household income ≥ $50,000, however, four reported participation in food assistance programs. Diagnoses were patient reported and included: 1) hypertension, *n* = 6; 2) hyperlipidemia, *n* = 4; 3) diabetes, *n* = 3; Obesity/Overweight, *n* = 6 (total yield more than 12 due to comorbidities reported). Characteristics of participants in the cognitive interviews are shown in Table 2.

**Table 2:** Demographic Characteristics of the Participants of Cognitive Interviews

Characteristics	Caucasian Race (*n* = 8)	Black/African American Race (*n* = 4)
Ethnicity (*n*)		
Hispanic Non-Hispanic Unknown	2 6 0	0 3 1
Age, years		
Range Mean	30–77 59.3 ± 13.7	37–71 59.0 ± 19.1
Education, (*n*)		
≤ High school graduate Some college ≥ Bachelor’s degree	0 6 2	1 3 0
Household Income (*n*)		
< $25,000 $25,000 to $49,999 $50,000 to $99,999 ≥ 100,000	2 1 4 1	1 1 2
Participation in Food Assistance Programs “yes”	2	2

In addition to previous pilot testing, responses from content experts informed the researchers of areas requiring more investigation using cognitive interviews. Overall, several themes emerged that related to terminology, nutrition content, familiarity with food items, and interpretation of instructions. The analysis of themes, their implications, and revision decisions are presented in Table 3.

**Table 3:** Summary of themes, their implications, and revision decisions resulting from cognitive interviews with patients (*n* = 12) concerning the Nutrition Literacy Assessment Instrument

Original Content	Issues Uncovered by CIs	Potential Action Taken	Implication	Revision
Nutrition & Health, *n* = 7 ^a^				
Prose Text	1. ‘Shelf-stable’ is an uncommon term, though all participants who noted unfamiliarity provided examples of foods that meet the definition 2. ‘nutrient-dense’ and ‘energy-dense’ were unfamiliar terms to most participants. All were able to give correct examples of foods in each category. Two were unable to answer questions about the terms correctly.	1. “packaged or prepackage foods” were suggested replacement terms 2. More examples could be given to explain the terms. Alternatively, different terms could be used, such as ‘nutrient-rich’ or ‘nutrient-poor’.	1. Even though the term is unfamiliar, it was interpreted correctly. Many fresh foods come in packages and are low in sodium, so either of the suggested terms could also be misleading. 2. Strong readers use clues from the text to interpret and apply unfamiliar terms. These clues were sufficient for the majority of the sample to answer questions, which suggests these are good terms to include for testing literacy.	1. No revision made. 2. No revision made.
‘An example of an energy-dense beverage is _____.’	Five of seven chose incorrect answers. Three chose ‘diet-soda’, acknowledging the belief that diet soda also has calories. One chose ‘unsweetened tea’ and another chose ‘black coffee.’ The latter two had difficulty with the term ‘energy dense’, and chose these options because they are healthy.	An alternative answer option to ‘diet soda’ could be used.	Incorrect answers reflect inaccurate understanding of the calorie contents of lemonade and diet soda. This may be an actual nutrition literacy problem, reflected in increasing intakes of sugar-sweetened beverages. Thus, this question could be a discriminating question.	No revision made
Energy Sources in Food, *n* = 6 ^a^				
All questions	Most questions were answered correctly by the entire sample. Two questions were missed by 1 or more individuals, but no consistency in logic for incorrect answers	Add more distractor options	If the entire sample answers questions correctly, the questions are more likely to be non-discriminating. Adding slightly wrong answer options may increase item difficulty.	Distractor options added
Household Food Measurement, *n* = 10 ^a^				
Instructions	Instructions begin with, “We all have different nutrition needs. Sometimes we eat food in the right amounts and sometimes we choose smaller or larger portions than might be best to achieve a healthy diet…” Two participants felt these statements personalized the portions and answered questions based upon the portions they serve themselves rather than what they thought the portions should be as intended.	These sentences were removed and tested with two more participants. Both answered some questions based on what portions they think they should eat and other questions based on actual portions consumed. One said, “…if people aren’t familiar with recommended portion sizes, they’re just gonna compare to how much they usually serve themselves…”	If people do not know recommended portion sizes, their only reference is their own experience, causing them to sometimes use the amounts they consume as their comparison. More instruction about the discrepancy between recommended portions and actual consumption is needed. However, answering based on actual consumption habits reflects behavior, which may be the action upon one’s nutrition literacy.	Instructions read, “Sometimes we eat food in the right amounts as advised by nutrition experts…For each food in question, choose what you think is the right portion size. This portion may or may not be the amount you usually eat. The portion amounts given in the question are also shown in pictures.”
“Pictured at right is one 5-oz chicken breast.”	9/10 participants who answered this question chose incorrectly. Most identified this portion as ‘about right.’	Delete question or modify	A different format for the question, similar to the format for the hamburger patty question, requiring selection of the right portion may be more effective.	“Using the photos above, choose the right portion for chicken.” [options are 3 oz, 5 oz, 10 oz]
Household Food Measurement, *n* = 6^a^				
“Pictured at left are 2 cups of pasta noodles.”	Participants suggested adding “cooked” before “pasta” to be clearer. Some incorrectly identified the portion as ‘about right’ because it appeared that was all that would be eaten at a meal.	Add “cooked”; Add side items to photo	While 2 cups of pasta is more than a recommended serving, eating 2 cups of pasta if nothing else is consumed is not excessive. People could use good logic and arrive at an incorrect answer.	“The spaghetti and meat sauce pictured at left includes two (2) cups of cooked pasta and 1 cup of meat sauce.” A glass of water and a side of garlic bread also pictured.
“Pictured at right is ½ cup of uncooked carrots.”	Most (7/9) incorrectly selected that ½ cup of uncooked carrots is ‘about right’ for a portion.	Delete question, keep unchanged, or modify	There is no consensus among guidelines as to what a serving of vegetables should be. Some say ½ cup, others 1 cup. ½ cup cooked is traditional standard. If item is removed, no vegetables are represented.	No revision made.
“Which portion of peanut butter as pictured above is equal to the portion for one serving according to a food label for peanut butter?”	Participants responded awkwardly to this question, some explaining the answer to this question was too obvious.	Delete question or modify	The question tests one’s ability to read the serving size on a food label, which is accomplished by another section of the instrument.	Question deleted.
Food Label & Numeracy, *n* = 6 ^a^				
“If your doctor has advised you to limit your total fat intake to 60 g per day, what percentage of your day’s intake have you eaten in one serving of macaroni and cheese?”	Question was missed by 4/6 participants. Most were unwilling to calculate a % and guessed “18%”	Delete question, keep unchanged, or modify	Calculating a % may be too advanced for most people, but the question may also be a distinguishing question. More data is needed on item performance before deletion.	No revision made.
Food Groups, *n* = 3 ^a^				
Butter	Might also be considered dairy, “…when you churn it…‘cause I thought it was made with milk…”	Keep unchanged	Butter is a significant source of saturated fat and little else. This may be a true nutrition literacy issue.	No revision made.
Lemonade	Incorrectly identified as a fruit, with logic that “if homemade, then it’s a fruit; if store-bought, then it’s an added sugar”	Keep unchanged	Whether store-bought or homemade, lemonade is an added sugar. This may be a true nutrition literacy issue.	No revision made.
All	No foods were unfamiliar to any participant	Keep unchanged	Familiarity with all foods is important to ensure that incorrect answers are due to problems with nutrition literacy.	No revision made.
Original Content	Issues Uncovered by CIs	Potential Action Taken	Implication	Revision
Consumer Skills, *n* = 6 ^a^				
‘Which green bean option is lowest in sodium content?’	Only question this section answered correctly by all participants	Delete, keep unchanged, or modify	May be a non-discriminating question, but not enough data from pilot testing to remove at this point. Sodium intake via processed foods is an important nutrition concept to include.	No revision made.
‘Which section on a food label provides the best information for choosing a whole grain food?’	Answered incorrectly by 3 participants because they felt it was a nuisance to read, ‘…the label is just full of all kinds of words I don’t even understand.’	‘best’ could be interpreted by some as ‘easiest’	Even if interpreted as ‘easiest’, those with stronger nutrition literacy may read ingredients lists more and be more comfortable. In pilot testing, ‘best’ was most often interpreted as ‘most reliable.’ Item testing is needed to determine action.	No revision made.
Hard Copy version	Formatting of this section was problematic for the 1 participant who used the hard copy of the instrument. He automatically disregarded option C (choices are equal) for each item, stating the “C” options did not seem to be “parallel” with the other answer options. The electronic version presented no similar problems.	Reformat to same presentation as electronic version, which gives all 3 answer option with a reference picture below.	Because no picture was associated with option C, it did not capture attention. Using the pictures as a reference below answer options removes the separation of this answer from the others	Paper version formatted according to electronic format.

^a^ A similar version of the NLit was previously tested via cognitive interviewing of 18 breast cancer patients with subsequent pilot testing [20]. The revisions determined from the pilot were incorporated into the present NLit along with minor modifications for the general chronic disease population. Thus, the varying number of patients interviewed here reflects the proportion of revisions to the domain between the two cognitive interview samples

In the Nutrition & Health domain, both the prose text addressing nutrition recommendations as well as the domain items were explored. The main concern with the prose text was the unfamiliarity with several nutrition-related terms. The most common terms with which participants had difficulty were “energy-dense,” “nutrient-dense,” and “shelf-stable.” For example, one participant stated that “Energy-dense…seems like a positive thing to me… If someone said, ‘those are energy-dense foods,’ I’d probably say those are good for me ‘cause they provide energy.” Another participant relayed a similar interpretation: “Energy-dense is kind of confusing… I buy energy bars… I just buy them because sometimes I skip eating and they’re better for me than anything else.” However, most were able to interpret the terms correctly with clues from the text and apply them to answer questions correctly.

There were no discernable concerns with the clarity of items for the Energy Sources in Food domain because most questions were answered correctly by the sample. When asked to rate the difficulty of this section on a scale of 1 to 5, a majority of the respondents (80%) rated it as a “1/very easy” or “2/easy.”

The Household Food Measurement section created the most problems for respondents. When asked to explain what the instructions were asking them to do, most responded that they needed to select which portion is the correct portion size using the information from the question and the picture. However, many respondents would start off answering the questions based on what they felt was the correct portion size, but then would begin answering the questions based on what they would eat or serve themselves, or what other family members would eat. For example, one participant answered questions and would justify her answers by saying, “‘cause that’s about what I serve [myself].” Similar statements were made by other participants. While many of the participants rated this section as “very easy” or “easy,” the frequency of incorrect answers for this domain was highest compared to the other domains.

No major concerns were uncovered for the Food Label & Numeracy domain. Participants identified the difficulty of performing some of the calculations as the main challenge. The last question (“If your doctor has advised you to limit your total fat intake to 60 grams per day, what percentage of your day’s intake have you eaten in one serving of this macaroni and cheese?”) was the most difficult for participants (or most often answered incorrectly), and this involved calculating a percentage.

The fewest interviews were completed on the Food Groups domain because previous testing of these items during our breast cancer patient pilot indicated familiarity with all food items. Most interesting were requests for more specificity with item descriptions, such as “is the rice white or brown?” and “is the tortilla a corn tortilla or flour tortilla?” Also, because the 'Added Sugars' category was added as a result of expert suggestion, researchers explored the lemonade and fruit punch items more than others. Lemonade was confused with the fruits group by 2/3 participants, both noting that it depends on the type of lemonade. One classified “fresh lemonade” as a fruit and “bottled lemonade” as added sugars, while frozen lemonade “would just depend on the brand.”

No major concerns were identified with the electronic version of the Consumer Skills domain, though one participant referencing the paper version found the formatting problematic. He noted that he automatically disregarded option C (choices are equal) because it appeared to him as a statement, and it did not have a food photo underneath this answer option similar to other potential answers.

As a result of content review and cognitive interviews, the NLit was reduced to 66 items. In total, 17 items were modified, five items were deleted, instructions for two domains were modified, and the formatting of question presentation was modified in one domain. There were some themes that arose in which no revision was made because the research team concluded from the interviews and review of transcripts that items missed were due to inaccuracies in nutrition knowledge, and not due to misunderstanding the intent of the questions and/or answer options.

## Discussion

### Key findings

The NLit is the first instrument designed to assess the nutrition literacy of adults in the primary care setting. Individual item review by nutrition and psychometric experts provided confirmation of content validity while also aiding in revisions to increase clarity. Involvement of patients from the target population using the cognitive interviewing approach largely assured researchers that the instrument was understood as intended, while also identifying potential confusing language that those interviewed were able to help rewrite for clarity.

In some cases, those who struggled with items often demonstrated lesser understanding of nutrition information. For example, the inabilities of some to acknowledge that homemade lemonade contains sugar and yet classify it as a fruit rather than an added sugar may represent a nutrition literacy problem. This could be important considering the potential ramifications of excessive intake of sugar sweetened beverages in the US population [34].

Assessing skills with portion sizing, represented by the Household Food Measurement domain in the NLit, proved difficult throughout the revision process. Research demonstrates that extensive training is required for accurate estimation of portion size [35], and even dietetics students in upper level training struggle to accurately estimate portion sizes [36]. Instead, the research team formatted items in this section to address the teachable skill of identifying the recommended portion size of various foods. In fact, this type of educational approach is often used for those who follow calorie-controlled or carbohydrate controlled diets [37].

Further complicating matters, sources providing portion size recommendations are inconsistent. For example, a portion of rice (1/3 cup) recommended by the American Diabetes Association is different than a portion (1/2 cup) according to the USDA [24]. The FDA’s Nutrition Facts Panel is required by the Nutrition Labeling and Education Act to list serving sizes that are reflective of actual portion consumption by Americans [38], and therefore suggests a different portion of rice (1 cup). Another example is fruits and vegetables. In the 2005 Dietary Guidelines, the USDA changed recommended amounts of fruits and vegetables from ‘servings’ to ‘cup equivalents’ [39], though this change was not reflected in other sources for portion recommendations, such as for the Dietary Approaches to Stop Hypertension (DASH) [40]. We accounted for these discrepancies in choosing item questions and correct answers that are consistent among all previously mentioned sources for portion recommendations, but the discrepancies limited our item pool, particularly in relationship to fruits and vegetables. Regardless, although there are logical explanations for the differences in recommendations (i.e. different nutrients of interest), the differences also may contribute to public confusion about recommended portion sizes.

### Limitations

An important limitation of this study is the time intensive nature of cognitive interviewing, which limits the sample size. As noted by Willis, the goal of sampling in cognitive interviewing, is not a large sample size, but to include a variety of individuals who are believed to represent the target population of the survey to be tested [41]. Although we used intentional sampling to represent the local urban racial and ethnic demographics, the data are not generalizable to other population groups or geographic locations. Additionally, data generated from cognitive interviews are not causal in nature [42], however, through our thorough analysis of the transcripts using the constant comparative method, and in the context of all prior formative work, all major saturated themes were identical between coders.

## Conclusions

As a result of revisions made to the NLit, courtesy of expert and patient review, the NLit is both content valid and understood by a sample of the primary care patient population. These steps are necessary and helpful in producing an instrument that achieves its target constructs and is understood as intended by the target population. Although further testing is required to establish validity and reliability, review by these important audiences increases the likelihood that the final instrument will accurately identify nutrition literacy issues rather than difficulties with navigating the instrument itself.

## Additional file

Additional file 1: Transcribed Cognitive Interviews. (DOCX 161 kb)

## Abbreviations

DASH: Dietary Approaches to Stop Hypertension
FDA: United States Food and Drug Administration
I-CVI: Item Content Validity Index
NLit: Nutrition Literacy Assessment Instrument
S-CVI: Scale Content Validity Index
USDA: United States Department of Agriculture
