# Supplementary material for: Engaging experts and patients to refine the nutrition literacy assessment instrument
Source: BMC Nutr. 2017 Aug 22;3:71. doi: 10.1186/s40795-017-0190-y (PMC5589339; doi:10.1186/s40795-017-0190-y)
Supplement: Supplementary file 1 — Transcribed Cognitive Interviews. (DOCX 161 kb) [file 40795_2017_190_MOESM1_ESM.docx]

**Adaptation of the Nutrition Literacy Assessment Instrument**

**Transcribed Cognitive Interviews**

**Dialogue from the participant is bolded**

Dialogue from the interviewer is regular text

***Interview 1***

So, what I’m gonna ask you to do is just to read a paragraph at a time, so it’s each paragraph has about three or four sentences or less. And so, after you read each paragraph, I just, I want to ask you a followup question, ok? Alright, so you can go ahead and read it there. Is that print large enough for you to read? **Yes.** Ok, go ahead and read out loud to us. ***(reading)*** **“This survey will help the research team to get an idea of the nutrition background information you already know and what may be missing. Instructions: Please read the text below and answer the questions that follow. Nutrition and Health. Eating a healthy diet and staying fit are key factors to your health and quality of life. A healthy diet helps to prevent or improve chronic diseases. Some of these diseases includes heart disease, high blood pressure, type 2 diabetes, and cancer. A healthy diet helps people achieve a healthy weight.”** Great. Ok, very good. Now, can you just kinda tell us in your own words, so if you were to explain what you just read, um, to one of your children, how would you tell it? What would you say to them? How would you describe it, in your own words? **Well, first of all, I would take out the word diet.** Ok. **Because a lot of times, I know for a fact, just explaining diets to my children and they’re like, “well that means I can’t, I can’t, I can’t.” They automatically associate diet with what they can’t have. So, I would just change that to a healthy lifestyle.** Ok, ok. **And then, um, I would also let them know that creating a healthy lifestyle change can deter you from having the following diseases that I just read about.** Great. Ok. **And, that would be my only critique of that … paragraph.** Ok, ok. But overall you found it, like if it was on a scale of zero to five, zero being really easy and five being really difficult, how would you rate that, the reading level of that? **The reading level, was, was easy.** Easy. **Yeah.** Ok, great. Ok, let’s go ahead and go, move on to the second paragraph. ***(reading)*** **“In order to follow a healthy diet, eat more nutrition-dense foods. Fruits and vegetables are examples of nutrition-dense foods. Plant foods are nutrition-dense because they provide more vitamins, minerals, and other need, needed nutrition. At the same time, they are low in calories. Eating more of these foods may improve weight control and decrease disease risk.”** Ok. Tell me a little bit about how you would explain that to somebody, what you just read, how you would explain that to your children. **Basically, I would just say, you know, that eating more fruits and vegetables, um, encourages a healthy weight control, for, for their life.** Ok, great. Very good. Ok, you’re doing great. Go ahead and move on. Ok. ***(reading)*** **“Whole grain foods, made, made from the entire grain seed of wheat, rice, oats, and barley, are known as whole-grain. Whole grain foods are nutrition-dense and they improve weight control and disease… I’m sorry, decrease disease risk.”** Ok, go ahead and move on to the next one. **Ok.** *(reading)* **“Lean protein: foods such as chicken and turkey, skin removed, low-fat dairy, lean meat, dry beans and peas, and fish are also nutrients-dense foods when compared in, with a few added calories.”** Ok. Can you just kind of walk me through and tell me what, how you would explain the last two sections that you read, um, to maybe your kids or to your mother or somebody? **Basically, I would more likely… Well, we’ll do my mom now (mumbles…) So, um, she for a while didn’t know what food were more rich in protein than others… And I was basically letting her know that chicken and turkey both are rich in protein, um, as well as, I believe, fish is too, and to stay away from the more fattier foods, which is, uh, ground beef. And that the process of it through our body takes a lot longer than these particular foods, so she um, we were arguing about that the other day, because she’s so used to those foods and it’s kinda hard to have a person break a habit that they’re so used to. But when she finally did come home (laughs), she found that it, she felt a lot better, she didn’t feel as sluggish and she had more energy…** Great. **…when she at those particular foods versus the other ones.** Great, so you’ve had these conversations with your mom… **Yeah.** So (can’t understand) Ok, great. Ok, go ahead and, and read on. **Ok. So…ok.** ***(reading)*** **“In order to follow a healthy diet, eat less: energy-dense foods; calories found in foods supply energy to the body. Some foods, such as chips, soda, fruit juices, and desserts, are high in calories and low in healthful nutrition. These foods are known as energy-dense foods. Taking in too many of these foods can lead to weight gain and may increase disease risk.”** Go ahead and go on to the next one. ***(reading)*** **“Refined grains: these foods are made from grains that lack a healthy part of the grain. Some examples include white bread and saltine crackers. Refined grains are low in healthful nutrition and too many can lead to weight gain.”** Ok. So when you look at those two paragraphs together, again, how would you maybe talk to your mom about that, in your own words? **I would let her know once again that too many… it’s, I mean it’s ok to have it, but to, to do it in moderation.** Ok. **Because if you have too many of it, either one of those things, you’re running the risk of gaining weight, and it’s, you’re not wanting to gain weight, you’re wanting to cut back.** Ok. Ok, great. Go ahead and move along. ***(reading)*** **“Saturated fats, cholesterols, or nutri-nutritions mainly found in animal-based products like meat and dairy products. These nutritions may increase the body’s level of cholesterol in the blood, a condition which is reduced blood flow to the heart.”** Go ahead. ***(reading)*** **“Sodium is found in table salt and many shelf-stable foods. Taking in less sodium may decrease blood pressure. High blood pressure is harmful because it makes the heart work harder.”** Ok. Yeah, sorry, yeah. Can you read these checks right here? ***(reading)*** **“Added sugars are found in foods that have high sugar content and have few nutr-, uh, nutritions. Foods such as soda, dessert, and candy. Taking in too many of these foods can lead to weight gain and cause high blood sugar for those who have diabetes.”** Ok, so, just taking in those last few, the saturated fat, sodium, and added sugars; again, can you just kind of summarize how you would explain what you just read to, um, your kids or your mother, that conversation with her again. **I would let them know that, um, when it comes to the saturated fats, we tend to find that, um, like, one example would be McDonald’s. When you’re eating lunch at McDonald’s on a daily basis, then you, you’re running the risk of causing your heart to have fatty tissue around it, to where it doesn’t work properly. And so, if it’s over the course of time, we’re talking years, then it could lead to a person having a heart attack. And I’m like, “do you want to have a heart attack,” and of the course the first answer is “no.” Okay, well then you have to stop eating McDonald’s; McDonald’s is not good for you. When it comes to, um, sodium, once again, fast food food or any restaurant that you go to, they, the portions are high and the sodium is tripled the normal amount that you would use if you were to cook at home. So I would advise my mom or my kids to, if you wanted something, you could cook that same dish that you would find at Applebee’s, you could cook that at home and then you control how much salt or how much sugar is in, is in that particular, uh, meal. And then if you want to continue to eat it, at least you know how much you’re eating.**  Great. **And then, um, for added sugars, everyone loves, well I won’t say everyone. I know the people in my family loves them; they want to have they desserts, they want to have candy, so once again, it’s kind of back to portion control. I’m not saying you can’t have it, but it’s not something that I would recommend that you would do in the morning for breakfast or, um, late at night right before you’re about to lay down and go to sleep.** Ok. All right, can you just, um, so that was a big section that you just read. Can you just, let’s, let’s rate it again on a scale of zero to five. Zero would be, you know, it’s pretty easy to read and I understood it. And five being kind of difficult, kinda like I could see other people having problems with this. How would you rate the readability under how well you could understand after, after you just read it? **Um, well for me it’s easy.** Ok. **Because I know about it, as far as if you were to say if I was my mom sitting here, would she be more likely to probably rate it a five.** Ok. **Because she really, honestly does not understand.** Dr. Gibbs (G): It would be all new information? **Yeah, it’s the new information, and we have saturated this, saturated that…** Ok.  **So if… so if you asked somebody who honestly does not know about nutrition, the bigger words can be confusing.** But you knew that? **Yeah.** Ok. So, you would rate this as about a zero? **Yeah.** Ok, great, all right, very good.

Ok, so we are just gonna, we’re gonna go ahead and move on to that first section right there. Um, which is Part One, uh, Nutrition and Health, and I believe it’s eleven questions? G: Yes. Um, ok, so let’s go ahead and move along to that. Begin, let’s just go through the similar process. And you’re just gonna read it aloud, and along with it, you want her to read the answers as well and then which of the answers she would select? Yeah, so just talk through as you’re reading through the answers, tell us what you’re thinking as you’re reading through the answers. **Oh, ok. *(reading)* “Foods such as blank should be included often in a healthy diet.” So you have “A, red meat; B, butter; C, whole grains; D, refined grains.” And I would say C, whole grains.** Ok. **Because red meat is, um, good to have but it’s not necessary. Butter is loaded with saturated fat, you know, depending which brand you get. And refined grains are, as I read above, it’s not the best one to have, so whole grains.** Ok, great, you were doing… That’s perfect. That is absolutely what we want you to keep doing, so we know it’s kind of a tedious process, but you’re doing absolutely what we want you to do. So, let’s just keep going at the exact same pace; keep doing exactly what you’re doing, because you’re doing perfect.

QUESTION 2: ***(reading)* “An example of energy-dense food includes: A, ice cream; B, air popcorn; C, fresh orange; or D, raw carrot sticks.” I would say A, because, um, it’s a dessert and it’s a energy-dense food. Air popcorn; I’ve had air popcorn before (mumbles). And a fresh orange is more healthier, but that’s not what the question is asking for. And D, raw carrot sticks is healthier, but that’s not what they, the question’s asking, so it’s A.** Ok, great.

QUESTION 3: ***(reading)* “Nutrition-dense foods such as blank should be consumed most often.” And the answers are “A, regular soda; B, French fries; C, an orange; or D, apple juice.” I would say C, an orange, um, because A is what you would consider energy-dense food and that’s not what the question asked. B would be the same, um, and that’s not what the question is asking, and D, apple juice would be the energy-dense food and not a nutrition-dense.**

QUESTION 4: ***(reading)* “A healthy diet is low in saturated fat, blank, sodium, and foods that… I’m sorry, foods with added sugars.” (mumbles) So it would be… I would say A, um, cause carbohydrates tend to… too many carbs, you will gain weight. And um, calcium is mainly, um, speaking of bones. And um, let’s see…** Niacin. **I’m honestly not sure what that…** Is? Ok. That’s fine.

QUESTION 5: ***(reading)* “Broccoli is an example of a food that is blank-dense.” So “A, energy; B, protein; C, nutrition; or D, calorie.” I would say, let’s see, C, and because, um, it’s a vegetable. And it is packed with a lot of, um… I would think it’s packed with some protein, but it’s more so, um, just think that I would put it in with a vegetable, it also helps to stabilize your blood sugars (mumbles).**

QUESTION 6: ***(reading)* “Saturated fat is mostly found in blank foods. A, animal-based; B plant-based; C, grain-based; or D, sugar-based.” And, I would say A, animal-based foods, and that’s because when you are eating, uh, meats, such as, like, pork chops or steaks, um, it’s coming from an animal, plus it’s, it does have a fatty layer, and if you don’t trim that off, then you’re eating it as well as the regular portion of the meat and the majority of the time you’re getting more fat that you think.**

QUESTION 7: ***(reading)* “Which meal is the most nutrition-dense? A, three-ounce hamburger on a wheat bun, twenty potato chips, and eight-ounce lowfat milk; B, one cup of spaghetti with meat sauce, one slice garlic bread, and eight-ounce lowfat milk; C, three-ounce skinless chicken, one cup of steamed green beans, and eight-ounce lowfat milk; or D, a pork chop…a four-ounce pork chop, a half a cup of steamed white rice, and eight-ounce lowfat milk.” I would say C, and that’s mainly because, um, the chicken without the skin, I have the vegetables, and I… so basically, I have a, um… a nutritional meat, I have, um, also incorporating my vegetables, and then I also have a dairy product, so it’s basically… I could probably add something else in there, but it’s basically the biggest required nutrition that I’ll eat out of everything that they have listed.**

QUESTION 8: ***(reading)* “Lowering sodium in the diet may lower blank, which is good for the heart. A, blood glucose; B, blood pressure; C, blood sodium; or D, blood cholesterol.” I would think blood pressure, which is B, because blood pressure affects your heart.**

QUESTION 9: ***(reading)* “An example of food with added sugars is… A, milk; B, baby carrots; C, brown rice; or D, chocolate pudding.” I would say D. And, because it is a packaged food and the majority of packaged foods are high in sugar.**

QUESTION 10: ***(reading)* “An example of an energy-dense beverage is… A, diet soda; B, lemonade; C, black coffee; or D, unsweetened tea.” Ok, with this one… has two answers.** Ok, tell us about that. **It could either be diet soda, or it could be lemonade, because both of them have sugar in it. So it’s gonna give you a false sense of, of energy. And then, once you crash, you know, you’re right back down to feeling sluggish, so.**

QUESTION 11: ***(reading)* “Which of the following foods is most likely to be high in sodium? A, a can of tomato soup; B, frozen corn; or C, frozen squeezed orange juice; or D, strawberries.” I would say A, because I’ve had tomato soup, and it is extremely high in sodium. So, um, and anything canned, they add stuff to it, so you’re getting double than if it was natural, from the ground.**

Ok, great, so let’s just stop after that section for a second. Let’s go back to that rating scale again, ok, and let’s just look at the questions themselves. So overall, how would you rate the reading, the ease of reading of those, of those questions, on a scale of zero to five. So zero, they were really easy, pretty easy to understand, I could follow them. Or five, they were really kind of difficult to understand, some of the language was confusing, um, it’d be hard for other people, um, other than myself to maybe read these questions. **Um…** What’s your perception? **I would say about a three.** Ok. **Some were easy, some were difficult.** Ok. Can you tell us which ones you thought were more difficult? **Um, the ones that are like, when it comes to energy-dense, anything that says “energy-dense” or “nutrition-dense,” if you don’t really understand that, then it can be difficult.** It can trip you up? **Yeah.** Ok. Great. Any others, besides those that say “nutrient-dense” or “energy-dense?” **No.** So you feel that those are the ones that some people might have the most, if they don’t understand the differences between those they’re going to have some problems with that? **Right, yeah.** Ok, what about some of the answers? I know you said “niacin,” you didn’t really know what niacin was. Um, and that’s, that’s definitely understandable. **Yeah.** Any other answers that were in there that maybe didn’t make sense or might, um… **No, that was the only one that kinda caught me off guard.** Caught you off guard, ok. Could you see that being relatable to other people as well? **Yeah.** Yeah, that niacin. Ok. Anything you want to add there? Nope. I think I’m good. Ok, great.

Ok, so we’re ready for section two. **Ok, so next page?** Yep. “Energy Sources in Food,” right? Great, and we’re looking at about ten questions there. Ok, so do exactly what you’ve been doing, you’ve been doing a gr—perfect, perfect. This is really hard to do, so you caught on really quickly. Ok. ***(reading)* “Energy sources in food. These questions concern carbohydrates, proteins, and fats, the nutrients that supply energy to the body. Use what you know about nutrition to answer the following questions. Foods like olive oil and butter are a source of… A, vitamin E; B, carbohydrates; C, protein; D, fat.” I would say… D?** Ok. **The fat… it’s a, I believe it’s a more healthier fat. So, um, but I know that um, I know it’s not protein. And I know it’s not, um, I don’t believe it’s not Vitamin E. It could also be a carbohydrate, I think, especially, like, the olive oil. But I’m sticking with D for fat.** Ok, can you just kinda tell us a little bit about why you would think the olive oil, um, why you would maybe lump that in with the carbohydrates? **Um… I guess, just taking a wild guess. I don’t necessarily know, so I’m just taking a wild guess.** Ok, yeah. That’s fine. **It may or may not be; I’m not sure. But I do know, without a shadow of a doubt, that it is a fat.** Ok. **That I do know.** Ok. Just wanting to clarify a little on our end there. Ok, great.

QUESTION 2: ***(reading)* “The blank found in fresh-squeezed orange juice is a type of carbohydrate.” So you have A for sugar, B for calcium, C for protein, or D for folate. And I believe that it is D, folate. “The folate found in fresh orange juice is a type of carbohydrate.”** Ok, and just kind of walk us through that. **Um, sugar is, I don’t believe it’s… what? Oh, that’s a tricky one!** Ok, why is it tricky. **Because if a person really, honestly does not understand carbohydrate or how to tell for it to qualify as a carbohydrate, then they may say all of the above. Even though that’s not one of the answers.** Ok. **So, um, but I, like I said, I think it’s D.** Ok, so just so I understand better, so in order for a person to really understand this question, they would really need to understand what a carbohydrate… **What makes it a carbohydrate.** What makes it a carbohydrate. So that could be difficult for some people. Ok. Um, but do you think, too, that it just has… maybe it’s the type of food that we’re asking here too, or any, any thoughts on, you know, the type of food that’s being asked? **Um, it could be that as well.** Ok. **But also, like I said, um, a lot of times when you have big words that most people don’t understand, then it makes the… it automatically makes the question difficult.** Ok. How about the way the question is worded? Just the way it’s asking it? **Yeah, it could be that as well. I mean, it’s a number of things that could lead to the question not…** Being confusing? **Yeah. It could be the way that it’s answer—the way that it’s asked, what type of food that they listed, and the fact that it has the word “carbohydrates.” And I’m the person who doesn’t know what carbohydrates are, what components make up a carbohydrate, then it’s like, “ok, um, I don’t know what to answer.” Cause I was gonna say all of ‘em. Just, you know, to keep it moving.** So, when we look at that question, if we could look at that difficulty rating of zero to five, this one probably pushing more towards the… **The five.** A five? **Yeah.** Ok. And if I told you that the answer to that question is “A, sugar,” how would you re-write the question? **Um, let’s see… I think I would say… I would say, “What carbohydrate is found in freshly-squeezed orange juice?”** What kind of carbohydrate? **Mmhmm.** Ok. Ok. **Because then, that would let them know that the answer is truly listed over here.** Mmhmm, so you were kind of thinking this was a trick question? **Yeah.** Ok, great, great feedback there. Ok, let’s go ahead and move on to the next question.

QUESTION 3: **Ok. *(reading)* “A source… A good source of blank is found in foods like eggs, chicken, and fish.” So, A, um “starch; B, protein; C, fiber; D, carbohydrates.” Protein.** Ok. **Is found in foods like eggs, chicken, and fish; and mainly because I know that, um, eggs, chicken, and fish is not a starch; neither is it a fiber. Um, and… I’m not quite sure if it’s a carbohydrate, but I do know that they all are packed with protein.** Ok. **So, because I know that that’s, when I’ve researched what foods are good in protein, eggs, chicken, and fish have always come up, so that’s the only reason why I know it’s protein.** Ok, good.

35:51 QUESTION 4: ***(reading)* “Which group of foods provides the most protein?” Is it “A, bread, rice noodles; B, bananas, applesauce, broccoli; C, pork chops, eggs, cheese; or D, peanut butter, olive oil, and salad dressing.”** Talk us through this. **I would say… um, it’s kinda tricky, because I know eggs is full of protein, and I know peanut butter is full of protein, so it would be like I would be picking something out of each one that I know has protein in it, and then making a group, you know, for an answer.** Ok. So you would come up with a… **A totally different answer, yeah.** Ok, so what are the, what are maybe the choices that are causing some, you know, difficulties? **I would say because of the combinations…** Ok. **Of what’s there.** Ok, can you kind of walk us through each, each choice and what, you know, what you know, you know, where you think the protein is, you know, where it could be lumped into maybe a totally separate answer because maybe the other two don’t seem to fit in there with a good source of protein? **Um…** So in option A? **In option A, I would say the protein would be the bread, because if it’s wheat bread then yeah; if it’s white bread, no.** Ok, ok. **Um, in B, I would say the protein would be broccoli.** Ok. **In C, I would say that the protein would be eggs.** Ok. **And in D, I would say that the protein would be, um, peanut butter.** Ok. So you’re, so for you, it’s kind of, again, it’s a trick question to you, because you’re looking at it as there’s one in each example or in each answer there’s a good source of protein. **Yeah, right.** Ok. Which could be confusing to people. **Right.** Ok, again we’re thinking, “you know this might be, this is a tricky question, it might be tricky for some people.” **Yeah.** Ok.

38:25 QUESTION 5: ***(reading)* “Which of the foods provides the most carbohydrates? A, oatmeal, potato, milk; B, eggs, peanut butter, cheese; C, peanuts, bacon, vegetable oil; or D, chicken, sausage, and fish.”** Go ahead and sort through those answer selections. **Um… I would say… A.** Ok. And why, why were you thinking A for that one? **Mainly because of the potato, the potato and the milk. Not so much the oatmeal, but, um, but yeah, I know that potatoes are high in carbs and milk can be too.** Ok. **Depending if you get whole milk versus skim milk.** Ok.

39:23 QUESTION 6: ***(reading)* “Which group of foods provides the most fat? A, rice, corn tortilla, saltine crackers; B, potato, pear, orange juice; C, carrots, avocado, yogurt; or D, mayonnaise, margarine, almonds.”** Ok, now talk us through. **I would say D.** Ok.  **Mayonnaise, margarine, and almonds. Not so much the almonds, but the mayonnaise and, and the margarine.** Ok.

40:01 QUESTION 7: ***(reading)* “Which breakfast is highest in carbohydrates? A, eight ounce orange juice, two slices of toast with strawberry jam; B, eight ounces orange juice, two scrambled eggs; C, eight ounces reduced-fat milk, two sausage patties; or D, eight ounces reduced-fat milk and two slices of bacon.” I would say A, the eight ounces of orange juice with two slices of toast with jam, strawberry jam. And mainly, not so much the orange juice, but the two slices of bread with both having the strawberry jam on it.** Ok. And why do you think the strawberry jam is grouped in there with, has more carbohydrate? **Because it’s a jar; it’s a, a artificial food, so it has a lot of preservatives inside of it, and so, pretty much… (mumbles)** Ok.

41:10 QUESTION 8: ***(reading)* “If your doctor asked you to eat more protein, which food is the best choice to increase protein and healthy fat in your diet? Salmon…” Uh, the answer, “A, salmon; B, sausage; C, cheese; or D, bread.” I would say A, salmon.** Ok. Ok, walk us through why you think salmon. **Because it’s, it’s also a fish. And fish is high, is high in protein. So, um, bread, to me is a carb, is grouped in with the carbs, and I believe cheese is too. So, salmon is just the, the most better choice out of the four; it’s more healthier.** Ok. Let’s go back to that, just when it says “healthy fat.” What do you think of when you… because I think that can, that’s a, um, I think those two words together can kind of trip people up, “healthy fat.” But what, when you think of a “healthy fat,” what do you think of? Just so I understand a little bit better. **Well…** The salmon, you would also categorize the salmon in there, or is there something else in there that you would also think of for “healthy fat?” **Cheese, is that what you’re saying with healthy fat? So…** How do you determine a “healthy fat,” I guess is… **I guess, just reading what’s on the back of the nutritional label.** Ok. **But also if you have a person who honestly does not understand how that works either, then they’d be more likely to still choose the wrong foods.** Ok. **Because a lot of people think that if you go low-fat, it is healthy. But when you read the back of the nutritional labels, a lot of the low-fat or low-sugar items are high in sodium, so I’m basically trading one bad thing for another. And since I am a person who’s living with high blood pressure, I really have to watch what I eat and my nutritional labels, so anything that is over the recommended amount for sodium, I, that’s not best for me to consume. Because I’ll pay for it later.** Ok, very good.

43:38 QUESTION 9: ***(reading)* “If your doctor asked you to eat less fat, which food should you eat less often?” Um, I would say, well the answers are, “A, black beans; B, regular salad dressing; C, potatoes; D, bread.” I would say B.** Regular salad dressing? **Mmhmm.** Ok, why would you say… tell us why you think regular salad dressing. **Because it’s a jar food; it’s packed with different ingredients and the majority of the time, those, um, those shelf foods have a lot of sugar, they have a lot of sodium, and they are high in fat content.** Ok.

44:26 QUESTION 10: **Ok. *(reading)* “Olive oil is more healthful than margarine because… A, it’s natural; B, it’s lower in fat; C, it’s lower in calories; D, it’s more healthier, it’s a more healthier type of fat.” I would say “D, because it’s a more healthier type of fat.” Margarine, uh, I’ve found, has a lot of different words that I truly do not understand.** Oh, yeah. **So I tend to stay away from, I think the rule is “if you can’t pronounce it, don’t eat it.”** So you read the ingredient lists on food labels, do you really? **Mmhmm. And if I can’t pronounce it, I put it right back on the shelf.**

Ok, ok. So, let me ask you this; that completes that section for energy sources in food, and this one took us a little bit more time to get through, so, um, and we definitely know that one of the questions would be rated higher on the scale of difficulty, but overall, how would you rate the ease of readability and understanding of this from that scale of zero, “it’s pretty easy,” to five being, “this one was more difficult, I, you know…” **This one’s a four.** It’s a four. And why would you rate it that way? **Because some of the questions were tricky to me, and some, um, were easy.** Ok. **But the answers really didn’t match the question…** So… **In my head, what was being asked.** So, for you in this section, so I’m understanding you correctly, the answers were probably more difficult to, to kind of really think through. The question, yeah, I understood the question, what it was asking, but boy some of those answer choices were a little bit more difficult to think through. **Yeah.** Do you think if we reworded some of the questions maybe differently that that would help, or you still think, ah, it has more to do with those answer choices? **I think it has more so to do with the answer choices than the way the question was worded.** Ok. Ok.

46:41 Ok, great. You are doing awesome! Ok, so, we’re gonna move on to the next section, which is “Household Food Measurement.” Um, and this has eleven questions, and this one’s probably the trickiest section, maybe? We’ll see. We’ll see. I know it’s tricky for me, so you might be able to fly right through it, so this one we really want you, um, just like you were doing before, just to really talk through it and let us know what you’re thinking on this one. Ok? And you can, for those pictures, with it being an iPad, make them larger, if that helps you. **Um, let’s see… number one. *(reading)* “Pictured is a glass that contains eight ounces of milk. This is… A, more than one portion; B, less than one portion; or C, about right for one portion.” And it is, um, C, about right for one portion. Because, um, relating to me, that’s about the amount of milk that I drink.** Eight ounces? Ok, so that is one serving? **Right.** Ok, ok.

48:02 **Two. *(reading)* “Pictured below is one five-ounce chicken breast. This is… A, more than one portion; B, less than one portion; or C, about the right portion.” And I would say C, about the right portion, and that’s because that’s about the size of the chicken breast that, when I do eat chicken breast, that’s about the size.** That you eat? So five ounces? Ok.

48:37 ***(reading)* “Pictured below is one cup of rice. This is… A, more than one portion; B, less than one portion; or C, the right portion.” And I would say, “A, more than one portion.” And that’s because when you, um, the rule of thumb is it’s supposed to be the size of your fist, and that looks like all of that would not fit in my hand to make a fist without it spilling out.** Ok, so more than one portion just based on that, that, that hand is what you think? Ok. **Right.**

49:24 ***(reading)* “Pictured below is one cup of strawberries. This is… A, more than one portion; B, less than one portion; or C, about right portion.” And I would say A… I’m sorry, I would say C, about right, because that looks about what I normally eat when it comes to strawberries.** Ok, one cup of strawberries? **Mmhmm.** Ok.

49:56 ***(reading)* “Pictured below are two cups of pasta noodles. Is this more than one portion, less than one portion, or about right?” And it’s A, more than one portion, because it goes back to fitting in my hand, it’s not gonna all fit without spilling over.** Ok, can you just explain to us a little bit more, when you do that rule of thumb with your fist, what foods are you normally thinking about when you measure with what fits into your hand? **Um, when it comes to, like, my fruits, my vegetables, and even with pastas and rice, and that’s because I have had issues in the past with being obese, and I ate more than what the normal size portion is. So it’s just a mental thing for me.** Ok. What do you think that is, when you think of a food size fitting into your hand, about, like, if you were to measure that out, if you were to take it out into a measuring cup and…? **It probably would be either right at a full cup or maybe a little bit less.** Ok. Ok, go ahead; you’re doing great.

51:09 ***(reading)* “Pictured below is a half a cup of black beans. Is this A, more than one portion; B, less than one portion; or C, right at one portion?” I would say, um, “B, less than a portion,” because it’s half, and a whole cup would be…** So again, you’re thinking of what fits into your hand? **Yeah.** Ok.

51:36 ***(reading)* “Below is half a cup of uncooked carrots. So, A, more than one portion; B, less than one portion; C, right at one portion.” And it would be B, less than one portion.** Ok. And again, is that… that hand? **A cup, yeah.** Ok.

51:58 ***(reading)* “Use the picture below to help answer question seven above. … So, A, more than one portion; B, less than one portion; or C, right for one portion.” I would say… C… well, no, because two slices of cheese is more than one portion, so A, more than one portion.** Ok, so you based that because of the slices of cheese? **Mmhmm.** Ok. Ok.

53:11 **So the next question is… should be the picture with the salmon in it, correct?** Yes. Yes, sorry. **Ok. So, *(reading)* “Pictured below is three ounces of salmon. Is this… A, more than one portion; B, less than one portion; or C, about right for one portion?” And I would say, um, C, right for one portion.** Ok. **The salmon looks like it’s about, it could fit in my hand. Not so much make a fist with, but it could fit in the palm of my hand.** The palm of your hand. So you’re going by the palm of your hand when it comes to the salmon? **Yeah.** As far as what looks like the right portion size. Ok.

54:10 ***(reading)* “Using the pictures below, choose the right portion for a hamburger patty. Three-ounce, five-ounce, or eight-ounces.” And it would be, um, three ounce.** Ok. Why would you say three-ounce? **Because it, it fits the bun; it’s not overflowing out of the bun, and it’s just, for that bun size, it’s just the right size.** Ok.

54:41 ***(reading)* “In the picture below, which portion of rice is the right portion? A fourth-cup, a half-cup, or a whole cup?” I would say a fourth-cup.** Ok, and why would you say a fourth-cup? **Because, um, once again, it’s the right amount that would fit in my hand without overflowing.** Ok. The cooked rice. Ok.

55:12 **Ok. *(reading)* “Which portion of peanut butter below is equal to one serving according to a food label for peanut butter, pictured to the right?” So, it says two tablespoons is a serving portion. So it would be B, the picture, um, with two tablespoons.** Ok. **Because that’s what it says in the nutritional facts, that a serving is two tablespoons. And the picture B is the only one that has exactly what the serving size is.**

Ok. So that finishes up that section; let me ask you a couple questions about that. Um, so, the pictures that went along with the, the questions, um, were they helpful to you or were they confusing? What kind of feedback can you give us? And it can be, um, on individual questions or you can just kinda tell us overall what you thought of the, the pictures with the questions. And look at the picture as a whole, not just what the question is asking about, but maybe what’s along in that picture as well. Just give us some feedback of what you were thinking about as you were, you know, looking at the pictures. **The pictures were very helpful.** Very helpful. **Because it allows you to have a mental… visual of what the question is asking. It was very helpful.** Ok. Would you say that you used the pictures or the amounts that were in the question, or both? **Both.** Both. Not one more than the other? Ok. So the picture was a reference point for you? What if the pictures weren’t there? **Then it would have been a little bit difficult to answer the questions because my half a cup may be different from a normal half a cup.** It’s harder to, to see that? **Yeah.** As far as it not being in the picture. **Yeah.** Ok. Overall with that section, how would you rate the difficulty of it, on a zero to five… **It wasn’t difficult.** Not difficult at all? How would you think other people might see that, that section? **Not difficult.** Think about your mother; if she were to go through that… **It would be easy because she has the pictures to reference.** Ok, so the pictures really helped to answer the questions? Let me ask you a question. Do you use measurement tools at home; do you use measuring cups in the kitchen? **Mmhmm.** Ok, so you feel like you’re pretty familiar with what a half a cup is, what one cup is? **Mmhmm. And I actually have a, when I was in weight watchers, they had the, uh, measuring spoons. So I use those still today for when I’m dipping, you know, food out of the pot to my plate. That way I know exactly the amount that I’m eating.** Ok. **And that helps, it helps me with portion control.** And it sounds like, too, for you, you know, you think of body parts to maybe relate to… is that something you learned? **Mmhmm.** Yeah. **They gave us this, um, it was, I want to say it was a “nutritionpyramid.com.” It was… basically they gave you, it was a visual based off a tape that, an actual cassette tape. Um, let’s see, what else was on there… I think it was… Like, it was saying, like the size of your fruit, your apples, your oranges, your pears should be able to fit in your hand like a baseball would fit. Um, when it comes to your meats, it should be on the order of, that size should be, should be as long as that cassette tape. So that way, you’re not, you’re not overeating.** Yeah, good. Were there any foods in this section that were less familiar than others? Any foods you weren’t familiar with? **No.** Ok, good.

***Interview 2***

Food Labels, ok. … Ok, alright, let’s have you, we’re gonna have you read the instructions first, and then we’re gonna have you go through, um, each question, read the questions and the answers aloud, out loud, and tell us what answer you would choose and how you arrived at that answer. Ok? Alright.

So everything you think, you just say out loud.

**Alright, Nutritional Facts; you’ve got a cup serving, and there’s 226 grams… Serving per container is 2 … Amount per serving is, uh, 250; calories from the fat is 110. Is that what you want, me to read all that?**

You don’t have to read the nutrition label; um, let’s see here.

As you’re answering the question, if you want to read through whatever you’re looking at to answer

Yeah.

**Ok.**

Um, yeah, so I think if we just scroll, just like that, there’s your first question.

***(reading)* “How many calories would you eat if you eat the whole container?” Well, five hundred.** Ok, tell us how you got to that answer now. **Well, you look up here, and you see it’s two servings, and two hundred fifty times two…** Ok. Ok, perfect. Ok, that’s all we’re wanting to know. **I’m a, I’m a label reader.** Great! Good, so this section… **It takes me forever to go through the store while I read everything.**

6:40 QUESTION 2 Ok, great. That’s great. Ok, so let’s go ahead and move on to the next question there. **Let’s see. *(reading)* “If you are trying to eat fewer calories and 500 miligrams of sodium per meal, how many cups of macaroni and cheese can you eat if you eat nothing else at the meal?” This is macaroni and cheese. Sodium… wow.** Just tell us what you’re thinking there. **A cup.** Ok. **Because if you go over a cup, it’s going to be more than five hundred.** Ok. Great, ok. Alright, doing good. **‘Course I never stop at a cup, if it’s just not the only thing I’m eating.** Yeah.

7:37 QUESTION 3 ***(reading)* “If you are limiting your total fat intake to sixty-five milligrams a day, er, grams, and you eat one cup of macaroni and cheese, what is the highest amount of total fat you could eat from others?”** Ok, so walk us through this. **(mumbling) twelve… total fat… subtract the twelve from the sixty-five, so… fifty-three.** Ok. So from that, you said it was simple subtraction, how you got to the answer? **Yeah.** Ok. **I did, I add and subtract in my head.** Ok. **So, how do you do that?** You did it perfectly well, actually. **Good.** It’s amazing how much we rely on calculators, isn’t it? **That’s what’s wrong with these kids today, they don’t know how to do anything without their cell phone.** Yep. **I hate calculators sometimes.**

8:53 QUESTION 4 ***(reading)* “How many grams carbohydrates would you eat in two cups of macaroni and cheese?” A lot. Sixty-two grams.** Ok. So how did you come to that answer? **Well, you take the amount of carbohydrates, is thirty-one grams, times two…** Ok. Good. **If you’re diabetic, that’s not what you want.** Ok, good.

9:24 QUESTION 5 ***(reading)* “If you are advised to increase your fiber intake, is macaroni and cheese a good food of choice?” I wouldn’t think so.** Ok, and why, why would you say that? **Because it’s all starch.** Ok. So you’re thinking about what you know about the food? **Right.** Ok. **It’s not, there’s no fiber, really; I wouldn’t think so. See, there’s zero fiber.** Ok. **I just…** Ok, yep. From past experience you answered that question? **Right.** Didn’t even have to look at the label for that one, huh?

10:16 QUESTION 6 ***(reading)* “If you are trying to limit your intake of saturated fats to seven grams per meal, how many cups of macaroni and cheese would you eat if you eat nothing else?” I’d say a cup. Saturated fat… two?** Ok. **Wow.** Ok, and you got that answer from…? **The label.** The label? Ok, looking at the label. But you first just assumed that a cup, yeah. And so, would you normally, you know, would you say you would normally have looked at the label, answering that? **Yes.** Yeah. Ok, ok. Good.

11:08 QUESTION 7 ***(reading)* “If you eat a half a cup of macaroni and cheese, how many grams of total fat would you eat?” Hmm. Well, total fat, ok, not saturated. I’d say six grams. No, I want twelve grams.** Ok, and how did you get to that? Tell us, tell us how you got to that answer. **Oh, wait, no, it wouldn’t! It would be six grams.** Ok. **‘Cause if it’s a half a cup and a serving is a cup, half of it would be six grams; yeah, I was right.** Ok. **That’s what I thought, I was right, but I thought, “Well, I’ll, I’d better double check.”**

12:06 QUESTION 8 ***(reading)* “How many grams of protein would you eat if you eat two servings?”** Ok. **I don’t think it’s a whole lot. I’d say about five grams.** Ok. **Two… two servings… I don’t think there’s a lot of protein in this… No, it would have been ten. So it was five grams of protein per serving, so if you multiply that by two, you get the ten.** What, so you said you wouldn’t believe there would be a lot of protein in macaroni and cheese. **I wouldn’t have.** Why? Why would you say that? **Well, it’s, it’s a lot of starch, plus it’s powdered cheese.** Ok, yeah. **Now if you go and make your home-made cheese where ya… I would think there would be more protein in it.** Ok. **‘Cause you know the, the cheese is protein.** What you’re adding exactly to it? **Yeah.** Ok. **So that was kind of a surprise.** Ok. More than you expected, then? **So, should I put what I would have put, or correct it?** Well, we want you to answer how you would have answered the question. If you were taking this survey without talking to us, how would you answer the question? **I would have said five… yes, that’s what I want. Ok.**

13:51 QUESTION 9 ***(reading)* “If you are advised to eat forty-five grams of carbohydrates per meal and you eat one serving of macaroni and cheese, how many grams of total carbohydrates could you eat from other foods at the same meal? … If you are advised to eat forty-five grams…” Hmm. Ok, subtract thirty-one, so that would be fourteen more grams.** Ok. You got that information just from… **Subtraction.** Subtraction, looking at that food label and making… Ok, doing some basic math there. **(mumbles)** Ok, a lot of information on it, isn’t there? **Yes, it is.** Ok, yes. **And it gets more and more every day, too.** Yes. **Just like gluten, they don’t never put it on there, so people that are allergic to stuff; I didn’t know what gluten was, and I was working in a nursing home and this guy had this gluten-free diet…** It’s hard. **What is gluten? They should teach that more.** Definitely. **‘Cause sometimes I want, if I’m gluten-free, I mean, I got a problem with it…** Ok.

15:06 QUESTION 10 ***(reading)* “If your doctor advises you to limit your total fat intake to sixty grams a day, what percentage of your day’s intake have you eaten on one serving?” Let’s see… (mumbles)… total fat… There’s twelve grams… (mumbles)… Forty-eight? That don’t seem…** Just walk us through what you’re thinking. **“If your doctor advises you to limit your fat intake total fat to sixty grams a day, what percentage of the day’s intake have you eaten?” Oh, ok. Makes more sense. Total fat would be twelve… I don’t, I guess I’m not getting it. ‘Cause if it’s twelve, and there’s not twelve here. “If the doctor has advised you to limit your total fat intake to sixty grams per day, what percentage of your day’s intake have you eaten in one serving of this macaroni and cheese?” Total fat… Oh, percentage! Not grams, percentage! Ok.** Ok. **That’s what I was, I was looking at the twelve grams, not the percentage. Eighteen percent, that makes sense now.** Eighteen. And tell us how arrived to eighteen percent. **I had to look at it three times. I had to read, I had to read this a couple times to figure it out. I was trying to go with the grams instead of… It says sixty grams a day, so what percentage, “what percentage of the day’s intake have you eaten in one serving?” So, you had to separate the grams from the percents.** So you’re looking at the, the column of percents that next to… **That’s what you gotta do.** Ok. **Because you want sixty grams total for the day, but what percentage of that is coming out?** Mmhmm. **And that’s not what was registering.** Ok. Ok, but once you did, so once you did you came up with that answer by looking at the percentage column? **Looking back, yes.** Ok. **I had to look back. And double-read it, cause there wasn’t an answer here for the grams.** Right. **But the percentage. I wasn’t paying enough attention to that.** Ok, to the answer choices as far as the percents, looking at the percent sign. **Right, yeah. That’s it, sometimes I gotta double-read stuff in order to…** That’s ok! That’s how most of us are, for sure. **Ok, next question…**

18:30 Ok, so, I just want to ask you a couple questions about, about this section. So, this one walked you through a lot of, about food labels, and it sounds like you read food labels normally as well. So, can you tell me on a scale of zero to five your perception of how difficult this section was, where zero is, “this is pretty easy, I do this a lot, so it’s not a problem,” to five being really difficult, so you know, “even though I read food labels, this is kind of, this is hard for me…” **I would say about a two.** A two? Why would you rate it a two as far as difficulty level? **I thought, since I deal with it every day, and I’ve worked in, I was a dietary aide, so I kinda had to watch the amounts, it made it a little bit easier.** Ok, ok. **But if I hadn’t done that, it probably would have been more a three.** You would have rated it a little bit higher as far as difficulty level? **Yeah.** Are there, were there any questions in this section that… it sounded like that last questions was… **Yeah, it kinda threw me off!** Any other questions in there that you were like, “ah, this is really difficult to answer this one.” Any one that stuck in your mind? **Not really.** What about some of the answer choices, any, any confusing answer choices in there that tripped you up? That you thought this doesn’t belong or I can see where this can be confusing for people or… or it didn’t seem like the, the, the answers might have matched up with the question we were asking? **Well, if I would have had it side-by-side, then I could have just kinda look over, instead of having to go back.** Oh, ok, with the nutrition label you’re talking about? **Yeah.** So keeping the nutrition label with… **With the questions, instead of…** Instead of jumping… **Because I have, I can’t seem to click in on the memory part.** Ok, ok. So… **My short term memory is…** Yeah. So with this, how often do you think people would rely on more what they think or know as opposed to… because it sounded like you did it with a couple of questions. How much, how many times, how often do you think people would maybe look at, you know, what they think that the answer is as opposed to looking at the food label? Do you think that would happen quite often? **They would more or less go with what they thought.** They thought. And why do you think that is? **Because it’s too much trouble to go back and look.** Look at the food label; do you think food labels are confusing for people to read sometimes, too? Or it’s just too time consuming? **The don’t want to take the time to do it.** Ok. Ok. Yeah. **Although it would benefit a lot.** Yes! **Especially if you’re diabetic, or… My sister-in-law is diabetic and she just hates it. But she has to watch her carbs.** Right. So it’s very important. **And I didn’t realize how much, you know, carbs are worse than, I mean fiber is fine, but I thought with diabetics, sugar. But it’s the starches, and I was like “Wow.” In terms of your compound sugar, which, yeah. I’ve learned a lot! Working in that…** Yeah, I bet! Great, great. Ok, great! Well you are doing wonderfully, so, um, we’re gonna go ahead and move on to the next section.

**Great.** So, let’s see here, let’s… where are we at here? … So the food groups. **Some of these I didn’t know a couple years back. They surprised me; I watched The Chew, and uh, I watched a lot; I go through a lot on the computer on nutrition and…** Good! Ok, so, now just do exactly what you did before, because you’re doing great. Um, read the instructions to us and then go through and answer… ***(reading)* “The next group of questions will give you the type of food and ask you to select the food group which it belongs to according to its nutritional balance (?). For example, bread would be in the grain group.”**

**An apple belongs in the fruit.** Ok, and tell us, yeah, just tell us… **It’s the only one that made sense.** There you go, ok.

***(reading)* “Which food group does the milk belong to?” Well, that would be dairy.** Ok.

***(reading)* “Which group do noodles belong to?” Well, it’s grain; I think it depends on the type of noodles it hits.** What other category were you thinking of? **Well, like a white… plain noodles, it’s a starch.** Ok. **Or ah, the whole wheat, it’s more of a grain; more fiber.** So is there another category that’s listed that you would consider as a better choice? **Not really.** Ok.  **I mean, that turns to a sugar after a while.** Ok. So if starch were on here, that’s how you would categorize it? **Yeah. Starch might work.** If starch were on there. But since starch wasn’t available, the next best choice for you… **Was grain.** Was grain. Ok. Now if it said, um, uh, if it said, um, maybe brown rice, then what would you, how would you categorize it? **A grain.** A grain, ok. But if it was, um, egg noodles, how would you categorize it? **Starch.** Starch? Ok. Ok, go ahead.

**“Which food group does orange juice… belong to?” And that would be fruit.** Ok. And, why would you say that orange juice belongs in the fruit category? **Because it’s a fruit.** Ok, ok. **It’s better to eat fruit than it is to drink because you get, you have to use a lot of oranges in order to make the juice, which causes more calories. I never thought about that until Dr. (?) said something. You’re … problems with being overweight, just eat the fruit, you’ve got the fiber in there. Well, that makes sense; but sometimes I just like a big glass of juice.**

**“Which food group does cheese belong to?” Dairy.** Ok. **Dairy and, well, cheese is a big protein too… But you, I would say dairy.** Ok. Why would you choose dairy over the protein? **Well, it’s a milk product.** Ok, ok. **So, which turns into cheese, which creates the protein.** Ok. But at first glance you would choose the dairy? **Yeah, the dairy.** First, would be your first choice. Ok.

**“Which group does the rice belong to?” Depends on what kind! I would say grain because there’s not a starch there.** Ok, again, when you say “depends on what kind,” can you just elaborate a little bit more, tell us… **Well, you got, you know, you got brown whole-grain brown rice, which hasn’t been hulled; and the white rice, it’s got the, they take the outside off so you don’t have that fiber and… they take away from it.** Ok. **Which, you have more starch than what you do the brown for the, the grain.** Ok. How about like a, if it was a pre-packaged, like a, you know, one of those Uncle Ben’s rice, you know, that you threw in the microwave, or, um… **It depends on if it’s white or, uh, whole grain.** What if it was like Spanish rice? **That’s a lot of starch.** Ok. What if it was… **If you add some tomatoes to it or peppers or onions, stuff like that, then you make it more nutritious.** What about long-grain wild rice, what would you say about that? **It depends on if it’s white or brown.** Ok. **You got the two different kinds, you got the Jamaican and you got the whole-grain long rice.** Ok. What would you say that the Jamaican rice would, would go into? **Uh, I know there’s a Chinese or an Asian place that uses only the Jamaican and they said it’s more flavorful, supposed to be better for you, but I don’t see much difference.** Ok, and would you say that would be a grain or a starch then? **More of a starch.** Starch, ok. **Because if I eat Chinese rice, or any kind of white rice, it makes me very lethargic, just kind of... I just want to sleep. And, uh, the brown rice don’t bother me. I don’t know why.** Ok. That’s great. **It’s weird. But my sister’s diabetic and I told her, I said, “Whatever!” And, uh, I started paying more attention to, uh, the way I felt after I ate, and if I eat brown I’m fine, but if I eat the white, I don’t… so…** Ok, good.

**Uh, “Which food group does a tortilla belong to?” Uh, grain.** Ok. **And that depends on what it’s made out of.** Ok, so if it was, um, so if it was… a white, white tortilla. **Starch.** Starch. But if it was whole wheat? **You got less starch…** Ok. So then it’s… **More grains.** Then it’s a grain? Ok. Corn tortillas? **I would say that’s more of a grain kind of a… it’s more wholesome. I’ve made tortillas before and it’s like, they come up with these spinach ones and… it depends on what you add to ‘em.** Yeah, yeah. So, so again it’s, you’re—**I’m not real fond of the… (mumbling).** So the tortilla to you is like the rice. It depends; if it had more information about what kind it was. **Right.** You think you’d be able to, able to categorize it easier. **Right. How much starch it has in it, how much protein, whatever.** Oh, you’d like the nutrition information? **Yeah.** Ok. **I’m just a nutrition…** Ok, good, good. **Especially with my mom being… she got that septis and everything, so I’m just really careful about what she eats.**

**“Which food group does the chicken belong to?” Protein.** Ok. **Chicken’s high in protein.** Ok, good. **Depends on if you leave the skin on it or not, but that’s the best part.** So why would the skin matter? **Well, if it’s a older chicken, you got more fat, and if you cook it with the skin on it, you might as well just eat the skin. If you take the skin off of it, you got less fat, more protein and you can still get a crisp crust on it. Less skin… (?) A whole chicken, and you still get a crispy crust.** So do you think it needs to say “skinless chicken” or would that change you answer at all? **Well, chicken’s protein no matter what.** Ok. So regardless if it said “skin-in,” “bone-in” you’d still… **Depends on how many calories you want to, how much you’re able to add and how much…** But it wouldn’t change what food group you would put it in? **No.** Ok.

**“Which food group does a pork chop belong to?” Well, that’s protein.** Ok. **If it’s in the meat group, it’s it’s, it’s gonna have protein.** Ok. **A lot of it depends on how you fix it.** Ok. **But it would still fall in the protein…** Good, ok. **The other stuff that you put on it, it adds on the other groups. Am I talking too much?** You’re fine. You’re doing great. **I don’t get to talk to adults very much.**

**“Which food group do carrots belong to?” That would be a veg, yeah, a vegetable.** Ok. You kind of wavered on that. **It grows under the ground plus it’s a root, so it’s a vegetable. I didn’t know that tomatoes are fruit. I’m like, “wow!” Yeah, it’s a, it’s a vegetable.** Ok, good.

**“Which food does butter belong to?” It’s got milk in it, but yet it’s a fat.** Ok. So you’re thinking between those two? **Yeah, that would… that’s a tough one. I, ah, my though was fat right at first.** Ok, so your gut reaction would be fat. **So, it’s, it’s fat, but it’s made with the fat from the milk, so it’s fat.** Ok, ok. That’s the one that you would most likely go for though, that’s, ok. **Oh, yeah.** Ok.

**“Which group does fruit punch belong to?” Depends on if it’s real fruit or if it’s a fruit cocktail.** Fruit punch that’s, yeah, the beverage, have you seen it? **Hawaiian Punch is nothing but sugary. Is that what they’re referring to?** Fruit punch? **Just like a Hawaiian Punch, or…** When you think of punch, when you think of punch, what do you think of? **I think of a sugary drink.** Ok. **Ten percent fruit and the rest of it’s sugar.** Ok. **I’d say added sugars.** Ok. So that was a little unclear, the term? **Right.** Fruit punch wasn’t clear to you? **Right.** How would you, um, describe it? **My, my fruit punch is grapefruit, orange, apple, I add grape juice and mix them.** So you, like, freshly squeezed? **Right. No, not freshly squeezed, but a hundred percent juice.** Hundred-percent juice… **No added sugars.** Ok, so like a cocktail that you’re think of. **No, not cocktail, that’s not a hundred percent.** Ok. But you’re thinking… Like a mixed juice? **Like a hundred percent, uh, grapefruit or a hundred percent cranberry.** Ok. **They’re more expensive, but…** So when, when she described what we meant by “fruit punch,” what—**That’s sugar.** So you said sugar. Is there a better word that we could use to describe what we mean by “fruit punch?” **Well, a fruit punch, uh…** You said “Hawaiian punch.” **Yeah, like Hawaiian Punch, that’s a…** Like a soda? The soda brand? **Oh…** That’s a brand of fruit punch. **I like “cocktail.”** Ok. **Hawaiian punch is a fruit.** Hawaiian punch is a brand of fruit punch. **Right.** Ok. **You can get, uh, like JuicyJuice, now that’s a hundred percent fruit.** Ok. **At, uh, WIC will let you get it ‘cause it’s a hundred percent. But then you get, like, uh, oh what do you call that, like, grape, grape juice, it’s, uh…** Grape juice cocktail? **Cocktail; it’s got ten percent fruit, the rest of it is sugar.** You’re right. **Water and sugar, that’s all it is. I consider that not fruit, but fruit punch is like a fruit.** Ok. **I make my own.** But in reading this, you would need someone to describe to you what was meant by “fruit punch?” **Are you talking about mixed juices or…** Ok. **‘Cause you can make a punch using different juices, or you can buy the Hawaiian punch or cocktail, that’s, that’s totally different.** If it had said—**It’s confusing.** If it had said fruit juice cocktail… **Right. That would have been a sugar.** You would have automatically said sugar. Ok. But fruit punch could be several different things? **You could say a hundred percent fruit punch, hundred percent fruit punch, or the other one would be a fruit cocktail.** Ok. **There’s a big difference.** Ok. **So when I see a fruit punch that, the sugary one comes to mind automatically.** Ok. Ok, good. **And I will never buy it.** Ok, great. **I never bought Kool-Aid or any of that kinda crap. It’s a cheap drink for kids, but all you’re doing is putting sugar into them. Give them something that… a piece of fruit cost about as much. Yeah, that’s what I was trying to say.** Ok.

**“Which food group does spinach belong to?” Vegetable.** Ok. **It’s a, it’s a green vegetable.** Yeah. Ok, good.

**Banana… That would be a vegetable.** Ok. **Or, no, fruit! I’m sorry. I’m looking at vegetable while I was thinking fruit.** Ok. **I know a half a banana is a serving, it’s like, but they won’t give those to diabetics.** They’ll give a half of a banana. Is that what you mean? **Yeah, a half a banana, they can’t just hand them a whole one. But they’ll give them sherbet. Sherbet is nothing but sugar.** You mean at the home that you worked at? **Right.** As a snack. **So it’s like, why you gonna give them that?** That is a good question. **It didn’t make any sense to me. They can’t have regular ice cream, but they’ll give them sherbet. Sherbet has more than regular ice cream.** That’s a good question. Good point, yeah, really good question. **It does, it really does! I love sherbet, but it’s too much sugar.**

**“Which group does a regular salad dressing belong to?” Fats.** Ok. **That’s all that is, is fat.** Ok, good. **And when I have one it’s got to be regular. I’d rather have a little bit than none. Take your fork instead of pouring it over your lettuce, dip your fork in your dressing, take a bite. You got the flavor without all the extra calories. I learned that from WIC.** Yeah. It’s a good idea.

**“Which food groups does lemonade belong to?” I’d say the fruit.** Ok. **If it’s fresh lemonade.** Ok. **If it’s bottled lemonade you’re getting more sugar.** So if it was bottled lemonade where would you, how would you classify it? **Added sugars.** Ok. What if it was frozen lemonade? **Ah, you got me on that one!** If we put “what food group is frozen lemonade…” **It just depends on the brand.** Ok, so… **A lot of those, some, some of those has, you know, fifty percent sugar, some of it only has slightly sugar.** Ok. **‘Cause I buy it where the frozen one where you have to add to it to make it sweeter.** Ok. And so with that you would classify it as… **More fruit.** More fruit, ok. **Hundred percent natural lemonade opposed to the generic that they’re… it’s cheaper and you’re gonna get what you pay for, pretty much.** Ok. **Cheaper is not always better.** Ok, so, so that’s finishing up that section. **I (mumbles) a fruit.** A fruit, if it just says “lemonade,” your first gut reaction would be fruit. **Right.** If it just asks “lemonade.”

So, with this section, let’s look at that scale again from zero to five, zero being “this was pretty easy” to five being difficult. How would you rate the difficulty of this, uh, this section? In answering the questions. **I’d say about a two, it was pretty…** Pretty easy? **Yeah.** Ok. How about some—**I didn’t, I expected to see something about a tomato, I was like “I know that.” People don’t know that a tomato is a fruit.** Thought we were gonna trick ya? **I was like, “wow!”** Yeah. So, um, but it sounds like from what, some of the feedback you gave us there might be some options missing. **Right.** Ok. So like the—**Like on the, on the juice, you know, that one… it’s not explaining itself very well.** Ok, so clarifying some of that. **Or the lemonade; is it fresh or is it bottled or…** Ok. Ok. Are there food groups you think were missing to categorize some of these questions into the foods? **Uh, no you got, they covered pretty good.** Ok. Are all of the foods familiar foods? **Yes.** Ok. Ok. Just, maybe, when we ask what kind of fruit punch or lemonade, maybe more clarification or be more specific? **Whether it’s a hundred percent or whether it’s a cocktail.** Ok. And that would have helped maybe categorize some of the foods a little bit easier for people to do. **Yeah. See I wanted some grapefruit juice. My mom comes home, she’s got this grapefruit cocktail; it’s like “what?” And I just couldn’t drink it. I gave it a, I, I just gave it away. It’s too sweet. But I get it, you can’t hardly find a hundred percent, unless you’re gonna pay out a (mumbles). And I got where I buy that Texas, uh, canned grapefruit or orange juice and it, the orange juice tastes like grapefruit. And, uh, it’s pretty good stuff.** Great. Well you’re doing, you’re doing awesome, so think you got it in you to do one more section for us? **Oh, yeah.** Ok, great, ok, good.

**Apple sauce, I just bought about ten of those yesterday. … Ok. “If calories are equal, of the two foods pictured below, which provides the most healthful nutrition overall?” This one is… Oh. The apple.** Ok, and why would you say the apple? **Because you got the apple, plus you got the fiber.** Ok. So the fiber is… Even though you love applesauce, you’d choose the apple only? **“An applesauce with no sugar added, an apple, or an applesauce with no sugar added and an apple are equal in nutrition.” No. A whole apple, because you got the nutrition plus you got the fiber.** Ok. **And you need fiber to regulate.** So you’d choose the apple for that? **Right.** Ok. **Unless it’s a small child, then I would peel it.** Ok. **But other than that, (mumbles).**

**Ok, “If calories are equal for one serving of each food pictured below, which food would make the most nutritional snack?”** So what do we have there? **“Fruit snacks that are made with real fruit” on the left, or “raisins” on the right.** Ok. **Let me see, let me read that again. “If calories are equal for one serving of each food pictured below, which food would make the most nutritional snack?” Well it depends on if it’s kids or if it’s an adult.** As far as who’s eating it? Or… **Right.** Ok. **I would say the melody [sic] because, uh, it’s variety, it has fruit, vegetables, and, uh…** That’s the fruit snacks? The fruit medley? **Yeah, it has fruit and vegetables … “Fruit snacks that are made with real fruit and raisins are equal in nutrition.” I don’t know if they’re equal in nutrition. I would think “A.”** A? And that’s, to you it has more variety in there? **Uh, it has, it’s a vegetable and a fruit. It’s mixed.** Ok, ok. **And raisins is just a fruit.** Ok. Have you ever purchased— **Kids, especially kids, they like different colors.** Have you ever purchased fruit snacks before, or are you familiar with them? **Uh, I like those gummies. But that’s just all sugar.** Ok, so you are familiar with them. Ok. **That’s not really, uh, I wouldn’t say a nutritious snack.** Ok. **But something with real fruit, kids are more likely to eat that than what they are to eat raisins.** Ok. **Raisins are more… it’s not good for your teeth.** Ok. **Unless they’re brushing their teeth all the time, you know, cause it sticks.** But if it was, if it was, you know, say, it was you or your mother that were choosing between the options, what would you choose if it wasn’t for, if you weren’t choosing for a little kid? **I would choose the melody [sic].** Ok. **If you had cranberries on there, I would choose that.** Ok. You’d choose Craisins? **Cranberries.** Dried Craisins? Dried cranberries? **Oh, ok, yeah.** Ok. Because you like them better? **Because I’m older and I don’t like raisins, really. My, my taste has changed.** But you like the dried cranberries? Yeah? Ok. So when you’re, when you’re reading that question and choosing an answer are you thinking about what you like better too? **Yes.** Ok. **What would I choose or what would my kids have chosen?** Ok. **Now for an older person, raisins, they would probably choose raisins or cranberries. Or dried fruits.** Ok. So, if it was between the medleys and the cranberries, or the craisins, what would you have chosen? **I would have chosen the Craisins.** The cranberries, ok. **For, uh, for me.** For you, but not necessarily because you think it’s more healthy? **My kids, it would have been the melody [sic].** Because they would like it? **For my mom, the melody [sic], because she doesn’t like raisins or cranberries.** Ok, but not necessarily because it’s more nutritious? **Right.** Ok. **I think it’s about the same. I don’t know what the nutrition of raisins is. Raisins have more sugar than most of the pears and apples and stuff.** Ok. **It’s just a natural sugar.** Ok. Ok, great.

**“If portions are equal, which meat pictured below is lower in fat content?” Oh, ok. Sirloin.** Ok. And why would you choose sirloin? **Because of the marbling.** Ok. So, with this the picture really helped. **Right.** Ok. Not because you knew the cuts of meat, but the picture really helped you decide that one. **This, this would be a, it’s a fattier. You got it here on the end and then you got it marbled. This would probably be more tender ‘cause you got more fat. This one would have less fat because it’s not a lot of…** So if the pictures hadn’t been there, what would you have chosen? **Sirloin, because I know the cuts of meat.** Ok. You know those, ok. Ok, but somebody who might not understand the cuts of meat, it would have been harder to do without the pictures. **They would probably not. Whatever they like.** Ok, yeah. Would have chosen what they know. **I mean, I like sirloin (mumbles) KC strip.** Ok, great, ok. **Got more flavor.**

**“Which beverage pictured below has more calories per eight ounces?” One, ok, one fluid cup. Naturally, the apple. Because it’s got, apple’s just a natural sugar.** Ok. **And the milk’s low fat, so you get less fat, but calories are usually just about the same.** As the apple juice? **No.** Oh. **I mean the different milks…** Oh, ok. Ok, I understand. **It’s not, it’s the fat content that raises… Most of the time it’s like a hundred calories for a glass.**  Ok. **But the hundred-percent, uh, apple juice, it has more sugar.** Ok. **Natural, but sugar. I wouldn’t think that they’d be equal (mumbles) sugar… fat…** So with that, more sugar is more calories? **Right.** Ok. **But it’s natural, so it would be different than having like a fruit cocktail.** Ok, yeah. **You only get ten percent juice, whereas it’s a hundred percent here.** Ok.

**“Which green bean option pictured below is lowest in sodium?” Well, frozen.** The frozen green beans? **Yes.** Ok, and why do you say the frozen green beans? **Because they’re froze without salt and when you buy canned goods, ninety-nine percent of the time they’re gonna add salt to it first. To preserve them.** Ok, ok. **They do have sodium-free ones, but they don’t, they don’t hold as long.** Ok. They don’t stay as shelf, they’re not as shelf-stable? **They’re not as shelf-life, as the salted.** Ok. **But if you freeze these fresh, they just… they freeze. They’re gonna last for a year.** Ok. **As long as you don’t defrost, then freeze, defrost. It makes a big difference.**

**Let’s see, “If portion sizes are equal in the food pictured below, which food provides the best nutrition?” Fresh. Fresh is always better.** Ok. Fresh is always better, why? **It always tastes better, I mean, you don’t have the freezer…** Ok. **I don’t know, I just like… Fresh is always better.** Ok. You like the taste of it better or you think it’s better in nutrition? **Well, see I need to say it’s better in nutrition. Not what I like better. I’d say they would be about equal. (mumbles)** Ok. **Zero trans fats, so they’re not adding anything to ‘em. They’ve just got, they’re quick-freeze. So they’re about the same, only those have been washed and cut, easier and more expensive. And you save money on this; and you’ve gotta wash ‘em. I’d say the nutrition’s about the same.** Ok. **I gotta make myself read all three. I go the first two and then I…**  With the pictures? **With the…** Is that because you feel like you do have to choose between them? **Right.** Ok. **Well it’s, it’s, I mean fresh, to me, is always better, in my opinion. But the nutrition, you don’t have any added preservatives.** Are you saying that with every question that you get you don’t think about that third option? **No, not every one.** Oh, ok, just this one. **But when I see potatoes, I’m like, yeah that’s all good!** Ok. **But the frozen, I mean there’s no difference in the nutrition. It’s just, it depends on who… it depends on how they’re packaged. Cause a lot of places, they’ll add all that salt. MSG they used to use, they don’t use a lot of that anymore.**

**“Which type of salad greens pictured below provide the most nutrition?” Kale.** You would say kale? **Yeah.** Ok, why would you say kale? **Because it’s a darker green.** Ok. Darker green leafy vegetables? **Right.** Ok. **And iceburg lettuce, it doesn’t, it’s just water pretty much.** Ok. **There’s no nutrition in it.** Ok. Are you familiar with kale, have you had kale before? **Yes.** Ok. **But not for a while.**

**“Which section on the food label pictured below provides the best information about sugar content?” (mumbles) “The nutrition facts panel and the package stating ‘no sugar’ are equal.” Nutrition fact… the back.** Ok. The facts panel? And that’s, I like what you, you know, what you just said is you can’t just go by the front of the package. **No, because anything can say “sugar-free” but it can have a lot of other crap to compensate for it.** So again, that’s why—**Some people can’t take, like me, if I have Equal it’ll give me headaches if I have too much. Or Splenda, it’ll bother me. Or even that Saccharine, I could use that, it don’t bother me.** Ok. **But, uh, no there’s a bunch of crap in that stuff and if you don’t read the actual… you don’t really know. That’s just cause “sugar-free” don’t mean much for Diabetics, you know, it’s not good for ‘em. (mumbles) And I know a lot of that stuff’s sweeter (mumbles) less is better. (mumbles) half a cup instead of a cup.**

**“If calories are equal, which food provides the best nutrition?” Hmm. Berries or juice, that is tricky. All natural, hundred percent smoothie. Blueberries or blueberry juice? That one, I’m kinda puzzled with. I’d say myself, to look at it, cause I don’t like Naked anyway, but I would say the blueberries because it’s a whole fruit, it’s fresh, it’s got the skin on the outside and a lot of nutrition is on the skin.** Ok. **And you can eat, you’ll actually eat less by using the fresh than the Naked because you have to have so much more to fill that bottle up.** Ok. **So you get more calories.** Ok.

**“Which section on the food label pictured below provides the best information for choosing a whole-grain food?” Well that stuff’s confusing.** It is for a lot of people. **Because it’s, it’s like a doctor when they go to talking. You know, for another doctor to be talking to ‘em or a nurse they understand what they’re saying. But to talk to an individual that hasn’t went to medical school it’s like baby talk, you know, you’re not gonna understand. This ain’t gonna work. Is that “unbleached?” Well, what’s unbleached mean? You know, you don’t know. For me, I know, cause unbleached is like a wheat flour. Hmm.** So what are you, what information are you having— **“Whole Wheat,” you know, it catches my eye with the “Whole Wheat.” Five grams. Five grams is half a cup.** Ok. **I think. I’m pretty sure.** So you’re looking at the front? The whole grain statement? **Yeah, because here it’s kinda, I don’t know, there’s too much crap on there.** Ok. **What’s a sulfate, I mean, how many people are gonna know what a sulfate is?** So there’s just too many unknowns on that ingredient list? **Right. I mean you’ve got corn syrup… a lot of people don’t know that the further, you know, the first part of it has more, it’s saying ‘I have more of this,’ the further down you go it goes less.** Ok. So when you’re trying to decide on this food at the grocery store, which piece of information are you gonna use? **Myself, I’d look at the back. And the front, but…**  The back, when you say the back, the nutrition, the actual nutrition label? **The nutrition, how much fat…** Gives you calories… So, neither the front of the package or the ingredient list? **Well the ingredient list goes with the… so I look to see, and now I look down here and say, you know, what’s listed first. And the further down you go you have that, like, fat and sugar listed the second ones, you know it’s gonna be full of crap.** Ok. **It’s not gonna be nutritious. But I would, I would first, if it was on the shelf, I’d see “Whole Wheat.”** Ok. **Well, let’s pick that up and see.** And what, and then, so let’s pick it up and see, then what would you look for next? **I would flip it over to…** Look at the… **carbs and the…** So you’d look at the nutrients, the actual nutrients? **Right.** But as far as the ingredient list… **Not really.** So since you only have those two choices, which one are you gonna choose or are they the same? **“Whole grain… ingredients… the whole grain package statement and ingredients list are equal sources of information.” Yeah.** So which one would you select? **C.** C? **That’s what I was saying, I would look at, I would go to both.** Ok. But when you say both, you would, you would look at that ingredient list along with the nutrition facts panel. **Right.** Ok. **I would look at this, then…** Everything? **Yeah.** Ok.

***Interview 3***

Ok, so if you wouldn’t mind, just, um, reading a paragraph at a time, and I’ll probably stop you after a paragraph or two, just to ask you some questions about what you just read. So go ahead and read out loud to us. **Ok. “This survey will help the research team get an idea of the nutrition information you already know and what may be new. Instructions: please read the text below and answer the questions that follow. Nutrition and Health. Eating a healthy diet and staying fit are key factors for good health and quality of life. A healthy diet prevents—helps to prevent… delay or improve chronic diseases. Some of these diseases are heart disease, high blood pressure, diabetes, and cancer. A healthy diet also helps people achieve a healthy weight. A healthy diet is high in nutrient-dense foods, such as fruits, vegetables, whole grains. A healthy diet is also low in energy-dense foods, refined grains, and added sugars. While these foods can provide energy, too much energy can lead to weight gain and chronic disease.”** Ok, I’m gonna stop you there. So, let’s say, um, you get home and your wife asks you, “Well, what did you do today? So what is it that they had you do?” And so, say you want to explain to her what you just read, about these two paragraphs. In your own words, what would you say to her? **Ok. Well, uh, basically they wanted to know how I was, my intelligence level on certain, you know, nutrients and stuff like that. Ok, and so, wanted to know, you know, basically if I knew that, you know, your fruits and vegetables are good for you and your sugars are bad for you and things like that.**  Ok, good. Um, what do you think of when you read the words “healthy diet?” What comes to mind when you think about a healthy diet? **Typically low sodium, low fat.** Ok. Ok. **Um, but you can’t, you can’t eliminate fat, you still have to have a certain amount of fat.** Right. **And basically, if you eliminate too much sodium, everything tastes terrible. So you have to find a balance, you know.**  Exactly. Yes. **Um, it’s just, uh, my wife is in the hospital and she had me try some of her food and it just tasted, it didn’t have any flavor at all.** Yeah. So you don’t, do you associate “healthy diet” as being positive or negative, or you don’t really think about it? **Well there’s certain parts of a healthy diet that, uh, you know… I tend to eat more fruit now that we watch the grandbabies than I used to because we’re trying not to feed them too much junk food. They, they prefer the chips and the cookies, but we usually give them, like, grapes, and the granddaughter likes the Cuties, the Clementines, the tangerines. The grandson used to eat the, you know, stuff like that, but I had, I got some oranges that just, I don’t know, maybe they weren’t ripe, but they were just really sour and I gave him one of those, and he won’t eat them anymore. That’s it, you know. One bad experience and…**  Right. **But she still eats them and, and so there’s usually grapes and bananas and oranges in. As long as the oranges are in season, they’re not always in season. You buy them out of season they’re not, they don’t taste as good. They tend to be more sour or tart, and I don’t want her to quit eating them like he did, cause we got a bag of them. So usually I sample them before I give them to her.** That’s a good idea. **And then that way if it’s, if it’s bad I won’t give it to her, I’ll try to find something else for her. Usually, we, we eat healthier now than we did because of the kids, we keep healthier foods around now because of the kids.** Ok, good, good. Ok, let’s go ahead and read, keep on reading. **Ok. … “In order to follow a healthy diet, eat less energy-dense foods. Calories found in foods supply energy to the body. Some foods such as chips, soda, fruit juices, and desserts are high in calories and low in healthful nutrients. These foods are known as ‘energy-dense’ foods. Taking too many of these foods can lead to weight gain and may increase disease risk.”** Ok. Just, after that, is, were there any confusing terms, um, or any words in there that might be difficult to understand, or anything that you would suggest that we change? **No, there’s not really. Um, not sure why the fruit juices would be there as opposed to… Because you would think fruit juice would be, um, healthy. Because, I mean, fruits are healthy.**  Ok. **Why wouldn’t the juice from the fruit be healthy?**  Ok. Go ahead and… So when you see “energy-dense foods” you think of not healthy? **Sugar, really.**  So when you read all of those things you thought of “not healthy?” **Yeah.**  Ok. Ok, go ahead and… **Ok. “Refined grains. These foods are made of grains that lack health, a health part of the grain. Some examples include white bread and saltine crackers. Refined grains are low in healthful nutrients and too many can lead to weight gain.”** What about in that, um, paragraph right there? Um, anything… **I think it’s good, it’s really pretty clear.** Ok. Anything that doesn’t seem to make sense? **No, I mean, I know that for a few years my wife has been eating wheat bread, but, uh, my daughter won’t eat wheat bread, so we usually keep white bread around instead…** Ok. Is “refined” a new term to you? **Um, yeah, I don’t really… I guess I don’t really understand “refined grains.” Um, as far as, um, what they are. Does that mean “processed?”** Mmhmm. **Ok.**  So, when you read the rest of the, that sentence, um, does that help to explain, or do you need more explanation? **I think you really probably ought to, uh, explain a little bit more about what the refined grains are.** Ok. **You know they’re…** So in addition to including the, some of the examples of “refined,” going a little bit more in-depth? Not too much, but maybe another sentence? **Yeah, if you, not too much, cause if you go in too much…** It gets to be like a chemistry experiment. **Yeah. And what was the, there was something that when I was going to school, they said, uh, you have to grab your reader’s attention, and you’ve gotta, because I think the attention span of the average person is like seven minutes or something like that. If you don’t get their attention by then, you’ve lost them.** Right. **So, if you’re giving them a lot to read, there’s something… I had a class where we were supposed to read books, and the teacher assigned the books. And the one book I got was done by a French author and about every fiftieth word, you had to go to a dictionary.** Right. **To find out what he was saying. And there was some stuff that he was saying, wasn’t in the dictionary. And I just, ended up just skimming, just, you know, though “to heck with it.” Cause it, I mean, that’s a pain to sit there with a dictionary, (mumbles) and you’ve got the time…** So here’s a question for you: The part of the grain that’s missing is the bran. **Ok, and the bran is the healthy part.** Yeah, but do you think that if I said, “the healthy part known as the bran” or… **“The bran has, has been extracted from it,” or something like that. Say that they found a way to pull the bran out.** Ok. And you think that’s something that a lot of people would know? **I think a lot of people know that bran is healthier for you.** Ok. Ok. But they, they wouldn’t know that that is what, the ingredient that’s missing, to make… **No.** Ok. **No, they wouldn’t know that.** Ok. Good. Ok, go ahead and, uh, move on, you’re giving us some really great feedback there. **“Saturated fat and cholesterol are nutrients mostly found in animal-based foods like meat and dairy products. These nutrients may increase the body levels of cholesterol in the blood, a condition which can reduce blood flow to the heart.”** Ok, tell me a little bit about that one. You kind of made a face at the end there. **Um, well, I think also the, there’s two kinds of cholesterol, isn’t there, one good for you and one bad, L-D-H and H-D-H or something like that?** LDL and HDL, mmhmm, yeah. **Ok, so, but this here kinda makes it sound like all cholesterol’s bad for you.** Ok. So, what could we do, how would you suggest that maybe we could re-write that? So, that maybe it includes that, you know there is, that the body does need some cholesterol? **Yeah, um…** Is there a way that you could suggest that maybe we could re-write that or do you have any suggestions? **I think as far as the, where it says “may increase the body’s level of cholesterol in the blood” and stuff, maybe down there you ought to specify the, the…** Harmful cholesterol? **Yes. Because I think, you know, the top is really, ok, both cholesterols are found in food, but, you know, if you eat too much of these you’re gonna increase the harmful cholesterol in your, uh, in your blood. So, I think, you know, that’s what you’re more concerned with is that one than the other one.** Ok. Good. Ok, go ahead and read on. **“Sodium is found in table salt and many shelf-stable foods. Taking in less sodium may decrease blood pressure. High blood pressure is harmful because it makes the heart work harder.” I think that’s really pretty clear.** Ok. When it says “shelf-stable foods,” um, do you have a clear understanding of what that is? **Well, they’re using the salt basically as a preservative in the shelf-stable foods.** Ok. **Um, that’s how I would interpret that.** Ok, would you have any… do you think that’s a pretty good term to use there or do you think, is there another…? **No, I think it’s probably a pretty good term.**  Ok. What foods come to mind with “shelf-stable foods?” **Oh, I think of soups, all your chips, I mean all your potato chips are. You know, I guess they’re now coming out with the baked potato chips that may not have much salt in them. But I know most of the stuff now is, you know, they’ll tell you right on the label, in fact it’s usually right up front, um, “no salt added” or “low sodium” or say something like that on the…** Is this something that you are looking for? **Um, when I buy potato chips, I look for the Lay’s in the blue bag, which is, I think it says like twenty or fifty percent less salt. But, uh, I actually prefer the baked ones because the baked ones are only a dollar a bag more for the same bag. Because all your chips, and this is where I guess it’s probably the, it’s probably the saturated fat, when you first open a bag of chips, the chips are nice, they’re not… but when you get down halfway through the bag of chips, they’re kinda oily, greasy, and, I don’t know, I think all the fat that was on the top ones or something dripped down on the bottom. So… But you guys probably don’t eat potato chips.** Oh, I do. **Haven’t you ever noticed that? The first part, the first bag, when you open it up, you pull out some chips, they’re all nice and, you know. When you get to the bottom you feel like your hands have gotten oily.** Oh, yeah, definitely. **So, I guess that’s the fat, I guess.** Ok. So let’s move on to the added sugars. **“Added sugars are found in foods that have high sugar contents and have few good nutrients. Foods such as soda, desserts, and candy. Taking in too many of these foods can lead to weight gain and cause high blood sugar for those who have diabetes.”** Anything in that paragraph that sticks out to you or… **No, I really think, I think that this is one that most people should know. Unless you’re talking about adolescents, you, um, I mean I would think that that was one that people should know.** Ok. Ok, can you kinda tell me overall, of everything that you just read, you know, tell me on a scale of one to five with one being “that was pretty easy, you know, I was able to go through that and understand it” to five being… **Well I think, definitely the added sugar was, I really understood that. I mean, you know, that’s something, basically why people eat these foods is, is the sweetness, you know, there’s the taste. And I guess, um, some people may use honey as a substitute for sugar but I don’t, it’s just kinda really inconvenient, you know. And everything… ok, if you buy pop, it’s got sugar, um, you know, I don’t… I kinda binge eat on sweets, I mean, I may go without, a couple weeks without eating anything, and then all of a sudden I walk by and the bakery’s got donuts on sale. Well, if it’s on sale and you can get, you know, seven dollars worth of donuts for four dollars, that’s a buy! You gotta have it, you know. And so, when, when uh, my daughter was growing up and lived at home, every Sunday I’d go buy a dozen donuts (mumbles) used to drive my wife nuts, but still, we wouldn’t eat much sweets in between, so donuts was kind of our little, “oh, we’ve made it through the week” or “we’re starting a new week” whichever way you want to look at it. But, I would say the added sugar, that’s pretty understandable.** Ok, so you would rate that as pretty easy to… **Yeah, to understand.** Ok. **Um, the sodium too I think is especially easy to read.** Ok, so you’d rate that as a one probably? **Yeah. I think with the saturated fat, probably, uh, maybe a three or a four because I think we do need to specify that the two types of cholesterol and it’s, you know, the one being L-D-C…** LDL, **LDL, you know, is the harmful one. That’s the one you’re concerned with. Cause you’re not, you’re not really concerned with the other one. You know.** Ok. **Uh, the refined grains, I think we just need to explain what the “refined” means and then we’re pretty good on that, so that’s probably, you know, a three or four too.** Ok. **Um, as far as the “energy-dense,” um, I think that’s probably about a three too.** Ok. Why would you rate it right there? That’s kind of, kind of there in the middle. **Well, uh, it’s cause, I mean, overall probably, I think people that have any, that have any knowledge at all understand it fairly easily.** Right. **But I think people that don’t have a good knowledge of it may have some difficulty with it, you know. Uh, cause, you know, you still say “energy” with food, you know, being active, being able to do things.** Ok, ok. **Ok, but, you know, uh, it’s like, uh, when you’re eating your carbohydrates, you want pastas, you don’t want sugars, you know, cause the sugars will give you a big charge right off. The pasta’s kind of like a time-release capsule and, you know, could last for hours. So, I mean that’s…** So do you think some people could get tripped up on when it says “energy-dense” and “nutrient-dense?” **Well I think it, the energy-dense makes it sound like these are food that are gonna give you a lot of energy right away, you know. Now I never used any of the supplement drinks like, I don’t know if they’re good for you or not.** Energy drinks? **Yeah, you know like five-hour energy and stuff like that. But they sound like they’d belong in this category. But I’ve never really used any so I can’t look at them and see what’s involved in it, is it ninety percent caffeine? Or it’s just like, you know, when you’re drinking sodas, everybody says that the Mountain Dew is probably the worst because it’s just loaded with caffeine. Well, um, occasionally I drink Mountain Dew because the variety, I don’t drink Coke-Coke-Coke, you know I don’t drink Pepsi, so when I go to a place that sells Pepsi, I’ll have a Mountain Dew instead of a Coke, so…** So, when it says “energy-dense foods” and the information that follows that, is there anything, is there a term that you could think of that, maybe, would fit in better than “energy-dense foods?” Um, that would maybe explain that paragraph? Whereas people wouldn’t think “energy” and being physically active, or… **Um, well, well, see I think, would “boost” be ok, energy-boost foods?**  Um, it would still have the word “energy” in there, you know, so… So is it “dense” that seems to be confusing? Or is it “energy?” **Well, ok, the “dense” means a lot. And…** That’s ok. **I’m not sure, let’s see this.** Did the word “calories” jump out to you at all in the sentence and trying to understand what “energy-dense” means? **Well, no, cause you do need some calories too, don’t you? I mean…um…** So when you read this paragraph, what kinds of foods come to mind that would be described as “energy-dense?” **Ok, things like sugars, um, because with sugars you get a quick boost of energy.** Ok. **Um, actually, well, I think mostly I’d say it’s the sugar-based foods that come to my mind. Um, so the things where—** And you said the fruit juice was surprising to you? **Yeah, that surprised me.** Because you considered that because of fruit, it was healthy? **Yeah, and maybe it’s not, because most of the fruit juices you buy now aren’t really fruit juice, they’re, um, they’ve added sweeteners to them or something like that to make them appeal more to the consumer. Um, ‘cause it does surprise me that you think if you’re eating an orange and then you drink a cup of orange juice, it seems like they ought to have the same benefit for you.** Ok. Well why don’t we move on to the questions and see how those work for you. **Ok.**

Ok, so you have done perfectly, so just keep doing what you’ve been doing and reading out loud. And again, go ahead and select an answer that you think is correct, but we’re not judging you on whether it’s correct or incorrect. **Ok.** So you can just go ahead and read it to us. **Ok. “Foods such as whole grains should be included often in a healthy diet.”**  When you read them, do you mind, can you just read out loud, like the choices and what’s going through your mind. **Oh, it’s uh, “Red meat, butter, and refined grains.” Ok, and we’ve already discussed the refined grains, they’ve had the bran pulled out of them. Uh, butter, I guess it’s, I guess I’m not really sure why butter’s got the rap because I hadn’t really looked at the label, but I guess it would be a high fat content. Uh, and red meat would have your, your high fat content too.**  Ok.

**Um, “An example of an energy-dense food is…” Uh, A is ice cream and that’s… The other selections were air-popped popcorn, fresh orange, and raw carrot sticks. Well, again, we discussed that, you know, your vegetables and fruits are good for you. Uh, air-popped popcorn is, well I mean, it’s corn that’s just been popped, and corn would be a vegetable, so, uh, and I guess as long as you’re not putting the butter and salt on there, popcorn would be healthy. Or at least filling.** Ok.

**“Nutrient-dense foods such as… should be consumed most often.” Ah, an orange would be the… Your choices are regular soda, French fries, an orange, or apple juice. And an orange would be the correct answer. Apple juice, again, you know, there must be sweetened juices or something like that to make it more appealing to customers.** Ok. **French fries are fried in an oil and typically, I don’t think most people would think about what kind of oil they fry it in.** Had you not read the reading before, would you have been debating between an orange and apple juice? **I would have debated between the apple juice and the orange too, because we would, and my wife’s usually up on these things, we usually give the kids fruit juices. We usually keep grape juice or apple juice there. Now we don’t give it to them straight, we usually, um, you know, give it like half juice, half water. But, uh, I wouldn’t have known that without…** Ok.

**Ok, “A healthy diet low in saturated fat, … sodium, and foods with added sugar.” Your answers are “cholesterol, carbohydrate, niacin, and calcium.” And again, on the, on the, I would say cholesterol on this.** Ok. **Uh, but again, you know, we’re not, we haven’t specified between the two cholesterols.** Ok. **I think that’s something that, you know, maybe (mumbles) to do.** Were, were there any answers on that one that was, um, that you would have debated between. **No, cause really, you need a certain percentage of carbohydrates. You want to be low on carbohydrates. Now, niacin is one of your vitamins, is it vitamin B or C or something like that?** Mmhmm, that’s right. **Ok, so you want vitamins too. I know a lot of people are taking calcium supplements, ok, so you need to, you’re gonna need calcium too, so it would be the cholesterol. But I think it needs to be specified.** Ok.

**Um, “Broccoli is one example of a … dense food.” And your answers are “(mumbles), protein, nutrient, or calorie.” And I think you answer is calorie—or I’m sorry, nutrient.** Ok. **Um…**

**“Saturated fats are found mostly in…” Um, then you know, your answers are “animal-based, plant-based, grain-based, or sugar-based” and the answer is “animal-based.”** Ok. **Um, so the saturated fats are found in the animal-based foods.** Ok.

**“Which meal is the most nutrient-dense?” And your selections are “three-ounce hamburger on a wheat bun, twenty potato chips, eight ounce low-fat milk; one cup of spaghetti in a meat sauce, one slice of garlic bread, and eight ounces of low-fat milk; three ounces skinless chicken, on cup of steamed green beans, and eight ounces of low-fat milk; four-ounce pork chop, one half cup of steamed white rice, and eight ounces of low-fat milk.” Uh, I would say it’s C, um, the three ounces of skinless chicken and one cup of green beans, and eight ounces of low-fat milk. I would, you know, if I didn’t choose C or if C wasn’t there, I probably would have chosen D, the four ounces of pork chop and, uh, half-cup of steamed white rice, and eight ounces low-fat milk. But I think it’s actually C.** Ok. **Um, you know, the potato chips obviously aren’t healthy for you, I don’t think the slice of garlic bread would be extremely healthy for you because usually with the garlic they’ve also got it in butter, you know. So I think it’s probably C on that.** So with each answer choice you were basically able to rule out with the other ones…  **There’s something here that doesn’t fit… The other one, the four ounces of pork chop… I think the skinless chicken is healthier than, than the pork chop.** Ok. **But otherwise, I don’t know about the green beans, the steamed white rice, I think that obviously the eight ounces of low-fat milk is (mumbles).** Ok.

**“Lowering sodium in the diet may lower … which is good for the heart.” Um, it’s gonna lower, your answers could be “blood glucose, blood pressure, blood sodium, and blood cholesterol.” And I would be a little bit confused on this one here… I think the answer’s B, but I’ve never heard of blood sodium, but just looking at it, it sounds like, ‘well that ought to be it.’ You’re lowering sodium in the, you know, which, so, you ought to be lowering the blood sodium, but is there actually something called blood sodium? See I’m not sure whether it’d be B or C is the answer then. I have to say B, because I know it’s gonna lower your blood pressure.** Ok. But you see those two answers as very similar? With sodium in the answer and in the question, you see that as, that could be a potential problem? **Yes. I see that, yeah.** Ok. **So is it the blood sodium or blood pressure?** It’s blood pressure. **Ok, I was right.** Yeah. **But see you would think it would naturally lower blood sodium too.** So it seems like a trick question with it… **It does, kinda.** Ok.

**“An example of a food with added sugars is…” Then your, um, answers could be “milk, baby carrots, brown rice, chocolate pudding.” I think that one should be pretty obvious, the chocolate pudding.** Ok.

**“And example of an energy-dense beverage is… diet soda, lemonade, black coffee, or unsweetened tea.” And I think that should be diet soda. Um, ‘cause they don’t eliminate all the sugars from them. They still have some sugar in it.** Ok. Ok. **Now, is that wrong or right?** What do you think? Tell us, kind of walk us through the other answers too. **Ok. Black coffee is basically, uh, it’s basically just caffeine and water. You know, uh, unsweetened tea is, is basically caffeine and water. Uh, the lemonade is possible, ‘cause people sweeten their lemonade, and is lemonade like a fruit juice? So, lemonade could be possibly an answer, so between the, I think it’d either be A or B, and I would say A because even though it’s diet, it’s usually not sugar-free.** Ok. So you see, even though it’s got the word “diet” on it, um, there’s typically still… **Something added to it to sweeten it, because it tastes sweet, so… So is it B? The correct answer?** B is the correct answer. **Yeah, ok.** But you see lemonade as a, as a whole fruit juice? **You do see it as kind of like a fruit juice.** Ok. So is that kind of confusing then? **I think so, I think, I’m still not used to the idea that fruit juices are not really that good for you.** Ok. Do you think a lot of people have that perception when they think of fruit juice? **Yeah, I think, I don’t think a lot of people realize that when they process the fruit juice, they’re adding, you know, sweeteners or sugars or something to it, you know. I think, you know, I mean, going back to the fruit juice thing, if somebody’s squeezing their own fruit juices, now, is that more like a fruit juice or is that more like fruit?** Well, nutritionally, you’re gonna get some of the benefits of the fruit, but you’re not gonna get all of the benefits of the fruit from just drinking the juice. **Ok.** So, it’s better than drinking sweetened fruit juice, but it’s not as good as eating the fruit. **I don’t think a lot of people realize that fruit juice has been sweetened, you know. I think a lot of people realize… cause, you (…)labels. You know, one hundred percent pure fruit juice makes you sound like.** Well, and a lot of them are. A lot of them are. And it’s not that drinking fruit juice itself is a bad thing, it’s just not as good as eating the fruit. Now what if we had, what if we had put on there, um, with the answer B, what if we had said that it was a frozen lemonade? Would that have made a difference to you? **Um, it probably wouldn’t have. I probably still would have chosen the diet soda. ‘Cause I’m… when you see the diet soda, you still think of, the word that hits you is ‘soda.’** Ok. **You know.** That packs the punch right there. **Yeah, I think it’s the word ‘soda’ is the one that makes you think ‘ok, this has been sweetened in some way or another.’** Ok. Ok, go ahead.

**Ok, “Which of the following is most likely to be highest in sodium? A can of tomato soup, frozen corn, fresh-squeezed orange juice, or strawberries.” I would say the can of tomato soup.** And you mentioned earlier when we asked what shelf-stable means to you, you automatically said it’s a lot of your canned soups, so that one was pretty easy for you to, to answer.

Ok, so with that section, I just want to ask you again, you know, kind of, let’s go back to that scale of one to five with that section overall. With one being “that was a pretty easy section, I understood it pretty well, it didn’t take me very long,” to five being “you know there were some spots that tripped me up, it was really difficult.” How would you rate that section overall? **Overall?** Mmhmm, overall. **There was some, a couple that were, you know…** So like the lemonade one… **Lemonade and coke, yeah. But otherwise, the rest of them are pretty straight-forward, I think.** Ok. So maybe a two? **Overall I’d say probably a two.** Two? Ok.

(Skipped to Household Food Measurement)

**Ok, “We all have different nutritional needs. Sometimes we eat food in the right amounts and sometimes we choose smaller or larger portions than may be best to choose a healthy diet. For each food in the picture, choose what you think is the right portion. The portion amounts given in this question are shown also in the pictures.”**

**“Pictured in this is eight ounces of milk. This is…” I’d say that’s probably, uh, the correct portion. So, I’d say that’s probably about the right amount of milk.** And are you looking at the picture, or are you looking at the amount, or both of them? **Well, I think, uh, I think probably the eight ounces is, is, so… ‘Course it doesn’t look like the glass is full, but it doesn’t look like the glass is empty.** So, do you think it looks like eight ounces? **Actually, that, looking at the picture, that looks like, uh, that looks like more than eight ounces because of the, it looks like more than a cup to me, and a cup would be eight ounces, correct? So that looks like more than a cup.** So the picture looks to be more than eight ounces. **Yeah, but not two cups. Maybe like a cup and a half.** Ok. But you think the amount of eight ounces is about right? **I think the amount of eight ounces is correct.** So do you think with that, if you were, if somebody were to look at both the question and the picture it could be confusing to them or misleading, or, or, or what do you think people are going to look at? **It’s just, to me, it looks like it’s more than eight ounces, but to somebody else it might not. You know, it’s just, ok, I do most of the cooking, so I’m used to pouring things into the measuring cup. And when you, now a measuring cup is fatter than that, you know, but usually a measuring cup is like, you know, a cup is about like this, you know, when you pour it in a measuring cup. And that, looking at that glass that’s probably like, it’s tall, and it looks like the milk is up over halfway, so…** Now I kind of lead you in that question in asking you does it look like eight ounces. **No, it looks more than eight ounces.** Would you have thought that if I hadn’t asked you that question? **After I read this I probably would have thought that was eight ounces.** Ok. Then that’s perfect, because we do have that last (…) that says “the portion amounts given are also shown in the pictures.” So, I lead you to question that. **Yeah. To me it does look like more than…** You wouldn’t have questioned it otherwise? **No.** Ok. It is eight ounces. **Ok.** So, ok, let’s move on to the next question.

**Ok, um, “Use the picture below to answer question two. Pictured below is five ounces chicken breast. This is more than one portion, less than one portion, about right for one portion.” I’d say that’s probably about right for one portion. Now as far as being healthy, it looks like they’ve probably given you a little extra potatoes than you…** (…) ask you about potatoes? **Yeah. So, go on to the next one?**

**“Pictured below is one cup of rice. This is…” I think one cup of rice is probably, uh, about right for one portion.** So C, you’d say, that’s the right one, that’s about right for one portion? **Yeah.** Ok, one cup. Ok. And when you think of the word ‘portion’ what does that make you think of? **Uh, the amount of a certain thing that you’re having for a particular meal. Um, so, I mean, you would have probably a portion of meat, a portion of one or two vegetables, and then maybe a cup of fruit or something like that.** Would you use a different word for, uh, to describe the recommended portion? The amount that you would be advised to eat by a dietitian? **Well you could put the word ‘recommended’ in front of the word ‘portion,’ but I don’t really think you need it.** Ok. It’s understood that’s what we’re asking for? **I have… ‘a healthy portion,’ or ‘the healthiest portion,’ you know. I think it’s understood, though, that that’s what you’re asking.** Ok, good.

**“Pictured below is one cup of strawberries. This is…” See, I don’t really eat strawberries, so one cup to me would seem like a lot of strawberries. But my wife loves strawberries, one cup to her probably wouldn’t be enough. So I would think one cup is probably a healthy portion of strawberries.**

**“Pictured below are two cups of pasta noodles. This is…” Ok, I don’t, I think that’s probably more than a healthy portion. Um, it just seems like two cups of pasta is a lot. I mean, that’s probably about what I would eat, but my wife would only eat about half that.** Ok. So your wife is your reference? **Well she’s, she tends to eat healthier than me.**

**“Pictured below is one half cup of black beans. This is…” I think that’s probably, um, about right for a portion.** One half cup, ok.

**“Pictured below is one half cup of uncooked carrots. This is…” I think that’s about the right portion.** Ok.

**“Pictured below is three ounces of salmon. This is…” I think it’s probably about the right portion. But it looks awful small next to those, the rice.** So it looks disproportionate? **Yeah, it looks like, boy if you’re ordering salmon and they bring you, you know, a cup of rice and only three ounces of salmon, you feel like you’re getting cheated on the salmon.**

**Ok, I think, you know, looking at the pictures below uh, and choosing the right portion for a hamburger patty, it’s probably three ounces, but I would actually order the eight-ounce.** So you think that the correct portion size would be the three-ounce one? **I would think that was…** Whereas your preference would be totally different. **Yeah.** Ok. **‘Course eight ounces is big. That’s actually half a pound. And then, are you talking pre-cooked or after it’s cooked?**

**“In the photo below, which portion of rice is the right portion?” Probably the one cup.** Ok.

**“Which portion of peanut butter is equal to one serving according to the food label pictured at the right?” Ok, serving size is two tablespoons. I think, well, it’s B. ‘Cause it says two tablespoons.** So you looked on the label to answer the question? **Yeah.** Ok. **Well, I wouldn’t have thought… Unless you’re putting jelly on it too, one just wouldn’t have been enough.**

Are you, were there any foods that were asked that were unfamiliar foods? **No, I think I knew, you know, I’m familiar with all of them. I don’t, I don’t regularly eat all of them, like salmon would probably, we maybe eat salmon twice a year. Uh, chicken’s probably about twice a week, but like I said, I’d sooner not have it in the house at all.** And you said that you do a lot of the cooking at home? **I do most of the cooking.** Do you do the grocery shopping too? **I do the grocery shopping. Well it’s, my wife is kind of, she has some health issues, so and she just, she has a very poor immune system. …** So, I was just curious about how often you use measuring cups and, um… **Yeah, when I’m making the food I do.** You do. **Uh, we’ve been (mumbles) lately that we have like a frozen pizza once a week and we have like chili once a week, so those things I don’t really use a measuring cup for. A lot of times when I’m cooking I’ll use a measuring cup. I think probably two or three times a week I’ll use a measuring cup.** Ok, ok. Um, those first several questions where it asked you, it said, it had the actual measurement, and then it showed the picture, what did you rely on more? Did you rely on the words, or did you look at the picture? **You know I, I think it was a combination.** A combination. **Because, you know, uh, especially the rice. Rice looks like they’re giving you a whole lot of rice, you know, but then you start to think about, ok, um, they’re saying a cup of rice and you look at it and think ‘I think this would be (…) took a cup and poured it out… And here again, it’s like, with rice, are they talking, are they talking a cup of cooked rice or a cup of uncooked rice? Because rice is like your pastas, you know, uh, I think probably a cup of cooked rice is gonna end up… … A cup of uncooked rice is gonna end up like two cups.** You’re right about that. Um, so is this about question number three? Is that what you’re thinking of? It says “Pictured below is one cup of cooked rice.” **I think so.** Ok. **Uh, because, you know, somebody that does the cooking is gonna know that rice will swell up. They may not know what a healthy portion is.** Do you think that’s the case also for the, the chicken, the… **No, cause I think, I think your meats, they’re (mumbles). They may not be though, so.** We are talking about cooked. **We are? So I think we probably need to specify cooked or uncooked.** Ok. **You know, because, uh—** And you think the same thing about, so the, the pasta, or the rice you said should be specified as cooked or uncooked. The pasta as well? **Yeah, those are things that tend to swell up, like that when you cook them.** Ok. **So, a cup of cooked pasta and a cup of uncooked, a cup of uncooked pasta would probably yield at least two cups of cooked pasta.** Ok, ok. What about the beans? **The beans? Uh, I think, I think beans swell, but I think different beans swell differently.** Ok. **Some beans I think absorb more moisture than others do. But I think, I think maybe if at the top of it, you know, when they’re first reading the introduction into it, ‘all portions are considered to be cooked weight,’ or something like that, then you wouldn’t have to say it on every one.** Ok. I’m only concerned that people won’t read it. They won’t read all the instructions. Yeah. Um, I’m curious, I don’t know about you, Heather… You said you’re the main shopper? Consumer skills? Let’s move on to consumer skills.

**Ok, “Choose the best answer for the questions that follow. A picture of the answer options are shown below. If calories are equal, of the two foods below which provides the most nutrients overall?” And, you know, we already kinda discussed that fruits are… and I think with applesauce, you’re gonna get the same thing with the apple juice. So I would say that the apple is gonna provide the most nutrients.**

**“If calories are equal, which food pictured below would be the most nutritious snack?” Um, ok, you got, uh, Mott’s fruit vegetables, you got some raisins. Uh, I would think it would be the raisins, because I think the raisins are just dried… I don’t, I don’t know what they do with the fruit and vegetable snacks, you know, whether they’re, whether they’re dried… You know, it says they’re made with real fruit, but they could have sweetened it…** Have you, around your grandchildren, do they eat fruit snacks, have you seen them before? **They eat raisins and occasionally, you know, I’ll buy the fruit snacks for them, but usually we just give them the real fruit.** Ok. … So they are kind of familiar. **Yes, but we don’t give them to them on a regular basis. Now, the raisins they both like, so we do give them the raisins.**

**Ok, uh, “If the portions are equal, which meat below is lower in fat content?” You’ve got the beef strip steak or beef sirloin. Well, just looking at the pictures, I would say the beef sirloin is a lot lower in fat.** So the picture helped you choose? **Yes.** Yeah. Ok.

**“Which beverage pictured below (misreads question) eight ounces, one fluid cup?” Ok… Well, the milk says it’s ten ounces, so I would say the fat-free milk provides. Oh, more calories per eight ounces. I think it’s one hundred percent juice that’s providing more calories. Then again, you know, you get…** So is that a distraction that the milk container says ten ounces on it? **I think so. I mean…** We might need to blur that out. Should it, or, um, yeah, blur it out or would a cup of milk, like if we showed a cup of milk, the portion sizes, and said it was fat-free? **A cup of milk might, a cup of fat-free milk, you know, might, uh… but, I mean, here again with calories is, you know, not all calories are good calories, you know. So, I would say it would be the apple juice would probably have more calories.** Ok.

**“Which green bean option pictured below is lowest in sodium?” It’s the frozen. Uh, again because salt’s added to a lot of canned goods. Preservative.** Ok.

**“If the portion sizes are equal in the foods below, which food provides the best nutrition?” And it’s the fresh potatoes.** Ok. **Now I know I used to hear that lot of your vegetables and fruits that people skin, that a lot of your nutrition is in the skin. Is that still true, do they still say that?** In fruits and vegetables? That’s true. **So when you peel potatoes, you’re actually throwing away a lot of the…?** Right. Fiber, at least.

**“Which type of green salad pictured has the most nutrition? Iceberg lettuce or kale?” Um, I’d say that it’s the kale. I’ve heard that iceberg lettuce is more filler, I don’t know if that’s true or not.** Are you familiar with kale? Do you… **No, I buy iceberg.** Ok. Have you seen kale at the grocery store? **I’ve seen it. But we, uh, kale is more expensive than the iceberg, and I kind of feel about salads the same way I do sweets. I’m kind of a binge eater, when I go on a salad, I will eat salads for, you know, two three days in a row, and then I think, ah, I’ve had enough salads. But I think it is the kale.** Ok. What if that option, instead of kale, was red leaf lettuce? Or green leaf? What would you choose then? **I think, probably, I think, um, the red leaf lettuce.** Ok, would that be more familiar to people to or do you think a lot of people would be familiar with kale? **I think more people would be familiar with leaf lettuce, because I know that, uh, I used to grow…** Spinach? **Spinach, yes. I used to be able to grow leaf lettuce. …** Well, the previous question had spinach as an option and we changed it to kale. **Oh, really?** The Popeye thing.

**Ok, uh, “Which section of the food label pictured below provides the best information about sugar content?” Um, well, I mean we’ve got the front of a box, and then we got the nutritional facts, so obviously, the nutritional panel should show, um, even though the one says ‘no sugar added,’ it doesn’t, it doesn’t say how much sugar’s there naturally.** Ok. Do you read labels now, since you’re the… **No, I don’t.** Ok. **If you read the labels, it, um, increases the amount of time you spend in the grocery store.** That’s for sure. How often, though, do you look at the front labels and make a judgment? **Oh, I do look at the front labels and typically what I’m looking for is, you know, no sodium added or low fat content or something like that. Like, um, we do eat Mexican-type meals a lot. So we eat, um, refried beans plus my granddaughter will sit there and just loves refried beans. So we usually try to buy the, you know, low fat content or, not no fat, but low fat.** Ok. Do you ever, when you see it has the low fat, do you then flip it over to read the label? **Make sure it’s…** Yeah, or just go and buy that? **No, I don’t do that. Uh, ‘cause usually we buy, what is it, Old El Paso, and they sell both. So I probably ought to take the two labels and compare, just to see how much lower it is in fat. You know, ‘cause you can say low fat and one of them has a hundred grams and the other is only like eighty. Well, it’s low compared to the other one, but it’s not really low fat. Low fat would be like twenty-five, maybe. So, I guess you should compare against, you know…** But you know that by, you know, looking at the nutrition label it gives you more information than the front label. **Yes, yes it does.** Ok.

**Ok, “If calories are equal, which food provides the best nutrition?” And we got blueberries or berry juice. And then, obviously it’s the fresh berries.** Ok.

**Ok, “Which section on a food label below provides the best information for choosing whole grain food?” Uh, it would be basically your ingredients list.** Ok. It’s interesting to me that you chose both, more information than the front of the package even though you said that you usually just look at the front of the package. **Uh, yeah. Well, when I, I’m afraid I don’t go there and think which is healthier. I mean, I know that the fruits are healthy, I know that the fresh vegetables are healthy, that you lose, even in the canning process, you lose some nutrients and stuff like that. Uh, I think you lose nutrients even in the frozen process. But you know, the only way to really get the most out of your fruits and vegetables is by fresh.** Ok. **Uh, I don’t think you lose as much, maybe, in the freezing of them as you do in the canning of them. And then, uh, I’m not sure what actually sitting in the freezer and sitting in a can does as far as like, you know, you look at your cans a lot of them say, you know, it doesn’t expire, best if used by like two years from now. So is it gonna taste the same today as it is in two years? Or how much nutritional value is there today and is it gonna be there in two years? Does it lose… Frozen food, usually, if you don’t use it quick enough it’s gonna get freezer burn, so then it’s, it doesn’t taste good, it’s, you know, dried out, and so basically if you open it up and it’s freezer burnt, you just throw it away and say, ‘ah, that was a waste of money.’ But…**

So for this section, Consumer Skills, how would you rate it as far as… **I think it’s, I think it’s pretty good.** Pretty… **One or two.** One or two? **Yeah, I think so.** Did the pictures help? **I think they do.** Anything that was unfamiliar that was listed? **No, I mean, I don’t know the, I think if somebody doesn’t do the shopping, they may not know much about the kale.** Ok. **But I think they would know about the spinach.** Ok, what about that Naked juice that’s listed for the berry juice? Is that a brand you’re familiar with? **No, I’m not really familiar with that brand.** Ok. But you were able to answer the question regardless of what the brand was? **I think so, yeah.** Ok. Great. What did you think overall of the, of this assessment, do you think it could be pretty useful? **I think it could, I think, you know, uh, you guys are trying to create a tool to help people, you know, if they’ll use it. Um, and it would help them a lot more if they would understand, you know. You know, I think probably, maybe some way to specify between the different cholesterols, ‘cause, you know, I think a lot of people are of the mind that all cholesterol is bad for you. I think a lot of people think that way. Which is not really true. So, um, and then, one of them that confused me most was, you know, that sodium affects your blood pressure, but…** And the blood sodium? **Yeah, you know, I’d think well obviously if you reduced your sodium you’re gonna reduce your blood sodium. That seems obvious, now whether it is or not, I don’t know. But what you’re looking for is reducing the blood pressure.** Right. And then the fruit juice one, that sounds like that one trips a lot of people up. **Yeah, I was confused too because, uh, shoot, I don’t know, I mean, how much do you lose by going to fruit juice over fruit?**

***Interview 4***

So, go ahead and get started with that first section, read us the directions, and then read us the questions out loud as well as the answers, and kind of your thought process as to why you’re picking the answer that you’re, that you’re choosing.

**Ok. “Calories in foods like olive oil and butter are a source of…” Um, you’ve got Vitamin E, carbohydrate, protein, fat. I’m gonna go with fat.** Ok. **Because Dr. ___ told me about those. I read his book.** Ok.

**Um, “The blank found in fresh-squeezed orange juice is a type of carbohydrate.” That’s sugar. Or, “the sugar found in…” wait, wait, wait. “The sugar found in fresh-squeezed orange juice is a type of carbohydrate.” Yeah.** Ok, sugar. Ok.

**“A good source of blank is found in foods like eggs, chicken, and fish.” Um, protein.**

**“Which group of foods provides the most protein?” (mumbles) not applesauce. (mumbles) “Peanut butter, olive oil, salad dressing.” “The most protein…” I’m wrestling between B and C.** Ok, just walk us through that then. **Because, well I know that broccoli provides a lot of protein. I never really associated banana and applesauce with protein. In fact, applesauce is more sugar than… So I’m gonna have to go with “pork chop, eggs, and cheese.”** Ok, good.

**“Which group of foods provides the most carbohydrate? Oatmeal, potato, milk; egg, peanut butter, cheese; peanuts, bacon, vegetable oil; chicken, sausage, fish.” Um, for carbohydrate, well, um, it’s not D. And peanuts, bacon, and vegetable oil are more fat than anything else, so it’s not C.** Ok, good. **So I’m wrestling with… well, actually I think it’s just A. Not even wrestling with B.** Ok. So just thinking through each of the options, you’re, yeah, you’re doing great with thinking out loud there. Process of elimination. So it’s giving us good information. **Good. Um, although I will tell you that if I would have taken this about a year ago or two years ago it would, I probably would have flunked it.** Ok, totally different answers? **Totally different answers.** Yeah. From reading the book that you read? **I read that book and I’ve read The China Study, and I’ve watched the movie, and I’m reading another one right now, I think it’s Dr. Ferman, I can’t remember the name. So I guess what I’m saying is I’ve had, over the course of the years I’ve had to do my own research. ‘Cause when I was first told that I was diabetic, they said it’s not curable. And I refused to believe that I’m gonna live life like this, and so that’s where I started doing research.** Good for you.

**“Which group of foods provides the most fat? Rice, corn tortillas, saltine crackers; Carrots, avocado, yogurt; mayonnaise, margarine, almonds.” You’re throwing me a loop because you added avocado in C, and yogurt. But I’m gonna go with D.** So by process of elimination? **Yes.** Ok. **I just know the three foods in D are fat.** Ok, together. **Yeah.** Ok.

**“Which breakfast is highest in carbohydrate? Eight ounce orange juice, two slices of toast with strawberry jam; eight ounce orange juice, two scrambled eggs; eight ounce reduced fat milk, two sausage patty; eight ounces reduced-fat milk, two slices of bacon.” I’d have to say the first one, A.** Ok. What makes you say that one over the others? Is there some food in there or what, what made the difference? **Well it’s the juice is sugar, the toast, you know, bread of course turns to sugar really fast, and strawberry naturally is sugar as well.** Ok. Ok.

**“If your doctor asks you to eat more protein, which food is the best choice to increase the protein and healthy fat in your diet?” Eat more protein… best choice to increase protein and healthy fat… Well, I know it’s not bread or cheese. Um, and sausage is bad fat. So, salmon.** Ok. What do you think of when you read “healthy fat?” **Um…** Is that pretty easy to understand overall, as far as what we mean by “healthy fat?” **No, but…** It didn’t make sense? **Well, it did to me, because, well of course reading Dr. ____’s book, there is no “good” fat.** Ok, ok. **There’s just different levels of fat. And so, when you see “healthy fat,” actually it’s the, uh, the one that takes longer to break down.** Ok, good. **But your question is right on. It’s hard, it doesn’t really tell you what a healthy fat is.** Ok.

**“If your doctor asks you to eat less fat, which food would you eat less often?” Well, it’d have to be, it’d have to be salad dressing.** Ok. **And on the bread, it depends on the type of bread, I guess.** Ok, so do you… What kinds of bread would make it harder to decide? **Harder to… no…** If we had, what kind of bread listed there would you have thought, ‘Oh, I might choose that one?’ Over salad dressing. **Oh, white bread. Or, you know, something that has a high yeast content, I guess. But still, salad, salad dressing would still win out.** Ok, even if it specified white bread, you would still lean more towards the… **Right.** Ok.

**“Olive oil is more healthful than margarine because… it’s natural; it’s lower in fat; lower in calories; more healthful types of fat.” Well, olive oil is natural and margarine isn’t natural. Um, it is lower in fat, depending on how much you use. And it’s lower in calories, therefore… And it has more healthful… So I need a “E, all of the above.”** Oh, ok. **But I guess I’d say it’s more healthful types of fat.** Ok, why would you choose that one over the other ones? **It’s most correct. ‘Cause it kinda encompasses the stuff above.** Ok.

Are there any foods in this, in these questions that we’ve asked that are unfamiliar foods? **Not so far.** Ok. Now, you, you actually flew through that fairly quickly! We didn’t have a lot of questions for you because you seemed really familiar with, with what you were doing and, um, great process of elimination. If we were to ask you on a scale of one to five how you would rank the, um, the ease of that section, with one being “that was pretty easy” to five being “it was pretty difficult,” how would you, how would you rank that and why? **If I was responding for myself, um, I’d say probably a two, or two-and-a-half. Yeah, relatively easy.** Ok. **But if I’m speaking for, before I’d read the books…** Yeah, so let’s think about a year or two ago. **I’d say it’s probably a five.** A five, ok. And why would you have rated that a five? Just lack of knowledge about…? **That’s exactly right, like, you know, as you’re growing up through school and what your parents teach you and what you learn on your own, nobody really talks to you about nutrition. Um, and I’ve seen several dietitians throughout my life, you know. It made, they gave me the, the standard (mumbles) that said, you know, the plate, the hierarchy of foods, stuff like that. And, and it’s wrong. And they have adapted. And so I guess that’s the biggest problem is that we are still taught and we’re inbred to believe that everything that the FDA has given us is cut in stone, that they’ve done all the research. And what I’m, what I’m glad to see and I’ve noticed it in commercials (mumbles), is they’re starting to really question, you know, what we do with vegetables and stuff like that. But, that’s why I say a five, just because I was never educated to that.** Ok, ok. **So I would say that there’s gotta be a precursor to this. And that’s what you’re doing is you’re putting together that brochure or book or pamphlet or whatever that is gonna help educate people.**

**“We all have different nutrition needs. Sometimes we eat foods in the right amounts and sometimes we choose smaller or larger portions than might be best to achieve a healthy diet. For each food pictured, choose what you think is the right portion size. The portion amounts given in the question are also shown in the pictures.”**

**“Pictured is a glass that contains eight ounces of milk. This is… more than one portion, less than one portion, about right for one portion.” Well, I’m gonna assume there’s no milk in the cereal yet, ‘cause I don’t see any. So I will say it’s about the right portion.** Ok. **But when you add the milk…**

**“Pictured below is one five-ounce chicken breast. This is… more than one portion, less than one portion, about right for one portion.” That’s about right.** So what are you using to decide on that? **The size of my hand.** Ok. **That is something I learned that I really value.** Was that for specific foods, or is that for just about anything, a rule of thumb? **Meats.** Meats? Ok. **Yeah, although I don’t eat meats anymore. I used to get the biggest.** That’s quite a switch then. **Yeah. I was upward of three hundred pounds. Since I’ve been… about one or two pounds a month I’ve been losing. Still got a ways to go.** Good for you! This is the hardest time of year, too, isn’t it? **Not anymore. I don’t have any of the cravings that I used to.**

**“Pictured below is one cup of rice. This is… more than one portion, less than one portion, about right for one portion.” You know, one cup is a hard thing to, for me to visualize. I have to measure every time I (mumbles) a cup. But I think this is more than one portion.** Ok. So the picture is what you were looking at? **The picture’s what I’m looking at. In the back of my mind when I didn’t see this is, I was thinking is that one cup before cooking or after.** Ok. Ok, so would it have helped in there if we had written “Pictured at left is one cup of cooked rice?” **Yes.** Ok. Then, when we’re asking, too, just a quick question, when we’re asking “portion,” what does that mean to you? **Um, what you would put on a plate at any given setting.** Ok. **‘Cause what did you say up top?** In the instructions? **Yeah.** Um, portions. **It’s hard for me to interpret that any other way.** Ok. Good.

**“Pictured below is one cup of strawberries. This is… more than one portion, less than one portion, about right for one portion.” I’m gonna say it’s, it looks like it’s more than one portion, just because of its bulkiness.** Ok. So the size of the strawberries more than anything? **Yeah, it’s, I mean it’s like if you were going to put them in a Vitamix, well that would be less than one portion, but, so it’s, it’s a cup of…**

**“Pictured below are two cups of pasta noodles. This is… more than one portion, less than one portion, about right for one portion.” I’m wrestling now.** Between the answers? Going back to the rice? **Right, well I’m wrestling with the question. … … … … … I’m gonna say it’s more than one portion.** Ok. Is that another instance there, like with the rice where we… it would have been helpful to put ‘cooked’ in there? Or… **Uh, for clarity’s sake, yes, but, but the difference is rice gets so puffy. Pasta puffs up too, but not to the extent of the rice.** Ok. **But, yeah, for clarity’s sake.** Ok.

**Because when I got to the noodles I just realized that you’re asking me, on the left you’re asking me “pictured below are two cups of noodles.” So it’s just making a statement that that’s two cups of pasta noodles.** Right.  **And on the right, you’re saying, in my opinion, is that more than a portion or less than a portion or about right for a portion. And I guess on the other questions I just assumed that the question was saying that it’s a portion and you’re asking me to judge the picture to determine if it’s a portion.** Oh. **So…** So you’re… say that again? **So, for example, um, let’s go to rice.** Ok. **It says, “Pictured below is one cup of rice.” So that’s a fact. “This is…” and then… I was comparing the picture, I get so literal, I was comparing the picture to the statement that it’s a cup of rice.** Right. **And so I was saying—**Do they match? **Yes.** Not ‘is one cup right?’ **My opinion that a portion should be less or more.** Ok. So what do you think would help to make that more clear, because you’re right, that’s not what we’re asking. Right. Because we state up here, um, that the portion amounts given in the question are also shown in the picture. So, let’s assume somebody skipped over that or didn’t understand that… **But above that you say “Sometimes we choose smaller or larger portions than are…” oh, no, “We all have different nutrition needs. Sometimes we eat food in the right amounts and sometimes we choose smaller or larger portions than might be best to achieve a healthy diet.” Maybe instead of “We choose,” “sometimes we consume smaller or larger portions,” instead of “choose.”** Ok. **Because a lot of times, you don’t even make a choice, you just consume, whether it’s a restaurant or especially when you’re at home you make a pot of oatmeal and eat the whole pot. Not saying I did that this morning.** Is there another way that, you know, that we could… do you think that maybe those opening instructions were missing the boat, maybe missing the point of what we’re asking afterwards? Or… Is it the way the question’s presented? **I think… from what I gather, you’re trying to get me to confirm the fact, right?** I’m trying to get, what we are interested in is ‘is an eight-ounce glass of milk, is that the right portion to eat or to drink, or should a person drink more or less than that?’ **But not, ‘is that picture an eight-ounce glass?’** Correct, that’s not what we’re asking. **So, I guess the clarity would come in… “Pictured is a glass that contains eight ounces of milk.** Should it be “Is this…?” Or… **‘Is this…’** ‘Does this represent…?’ **‘Does this represent a portion?’** Ok. **Or…**  Could we do, ‘is this equal to more than one portion?’ Would that help? We can’t use the word ‘represent.’ It’s too hard of a word. Yeah. **Ok.** ‘Is this considered…? Is this…?’ **Well, I think all you’d have to add is, ‘for each food pictured, choose what you think is the right portion size for you.’** Ok. **Or what you… I’m trying to personalize the portion.** Ok, individualize it to you? **Right.** Ok. **Right. ‘Cause that’s not what I’m getting, not what I got at first.** Ok. **So I don’t know if that’s in the instructions or part of the question or how that would play out. But to personalize, individualize this.** Ok. So, it seems like there’s, um, I don’t know, tell me if I’m wrong on this, is there just, is there kind of a disconnect, or is there something missing between the instructions right there at the top and how we’re asking the questions that could be confusing? **“We all have different nutrition needs.” Ok. “Sometimes we eat foods in the right amounts and sometimes we choose smaller or larger portions than might be best to achieve a healthy diet.” That is a very long sentence that I actually stumbled on the first time I read it. Um, so if you broke it up and said, “Sometimes we eat food in the right amounts,” and then the second one is…** “Sometimes we choose portions that are smaller…” **“Choose” is the word that I think I’m kinda…** You like ‘eat.’  **Having problems with.** Ok. You like, “Sometimes we eat” or “Sometimes…” you used consume—**Right—**“Sometimes we consume smaller or larger portions than might be best to achieve a healthy diet.” Leave out the second, or the third sentence. **Yeah.** Ok. **I think that’s really, the, the “choose” is probably what’s…** Ok. **Um, so then, ok, so then it establishes that nothing is equal. … “For each food pictured, choose what you think is the right portion size.” For your circumstances.** Ok. **Because you’ve already established in the very first sentence that we all have different circumstances. So if you say, “Choose what you think is the right portion size…” ‘Cause when I’m looking at this, I’m thinking the general populous. And I don’t have a personalized view for your circumstances.** Ok. Ok, so you’re thinking, um, that this is possibly, this is asking two different things. There’s the general population with the portion sizes, but this is specifying according to individual needs? **Right.** Ok. **You know, for example, um, my circumstances--** So if we were to ask, if I were to ask you what would your nine-year-old grandchild, would the portion for her—or him?—her, would the recommended portion for her be different from what a portion would be for you? **Oh, absolutely. Yeah.** Ok. What about between you and your wife? Would the portion size be different? **I think it is.** Ok. **I mean, we just eat different portion sizes to start, but…** Ok, ok. So when you think of, do you think of the word ‘portion’ or ‘serving’ different? So, what, what this sounds to me is you’re saying how much I would serve myself, how much I would portion onto my plate. **Right.**  Not how much, necessarily, is recommended as a portion. **That’s a good point, ‘cause when I think of ‘portion,’ I think of the medical or scientific community has defined what a portion is. But when I think of ‘serving,’ it’s what I take.** So, we are wanting the portion. That’s what we are wanting people to evaluate. **Ok.** So, are we conveying that? Are we, are we describing that in the question, or are you confused about which we’re wanting? **When I… all the questions that I answered for, I took from what I’ve learned from the medical and health community.** Good, that’s what we want. **Ok.** Ok, great. But is that first sentence up there in the instructions, “we all have different nutrition needs,” do you think that’s confusing, maybe? Because then we’re specifying to individual needs? Or is it ok? **Well, I actually, I actually question why you have that or the second sentence in there, because what you’re really looking for is for each food pictured, choose what you think is the right portion size.** So you think that we could just completely maybe even eliminate those first two questions and just bring in the instructions, “for each food pictured below, choose what you think is the correct portion size.” **Right.** Ok. Ok, and that would have made it more understandable where you weren’t going back and second guessing, is this the medical portion or is this individualized to me? I love that, because I didn’t put those sentences there, other people told me to put them there. So, it just confirms what I thought! Ok, great, ok, that’s great feedback. **It was difficult.**

**I’m thinking that’s less than a portion.** Ok. **It’s two-thirds cup.** Ok.

**Half a cup… I’m gonna say it’s more.** Ok.

**That looks to be about five ounces. I’m gonna say more.** So, you’re answering that you think it looks like it’s more than three ounces? **Yes.** Not that three ounces is more than a portion? **No. You’re correct.** Ok, so the question is asking, ‘is three ounces of salmon, is that more than one portion, is that less than one portion, or is that about right for a portion?’ **Oh, you didn’t ask that. You said ‘pictured below is…’ what you’re asking now is, take out the picture below, ‘is three ounces of salmon…’** So, is it the way that the question is structured that, it says ‘pictured below,’ so that made you think that you’re supposed to answer whether the picture is right or not? **Right. I told you I’m very literal.** No, that’s helpful because, um, you’re saying that you think that looks like five ounces, not three ounces. **Right.** Is that how you’ve thought of every question as well? **Yes.** So you’ve read the sentence then you’re looking at the picture and you’re comparing to the measurement in the sentence to what it looks like on the plate. Ok. How, how would you… So let’s go to the salmon, how would you re-write that question? **How she said it. Um…**  Even with the picture there? ‘Is three ounces of salmon, as pictured below…?’ **Or, no, ‘is three ounces of salmon more than one portion, less than one portion or right?’ You don’t have to tell me that there’s a picture. I see that there’s a picture. And basically what you’re doing on every question is you’re telling me, ‘there’s a picture there.’ So if you just had, ‘is three ounces of salmon more than one portion, less than one portion, about right,’ I’m with you.** Ok. But there, to have it in there is confusing because it makes you think you’re judging the picture. **Right, because I’m trying to, I feel that I’m trying to help you communicate. That’s why I’m looking at the question and comparing the pictures, and so my approach is totally different.**

**Now, see, here you’re gonna have to use the photos. … Five ounces.** Ok. So these are a little bit easier to figure out. **Yeah.** Ok. **Yeah, this format is, is better.** So, asking a person to choose a portion size, the, ok, how we’re asking the question? **Right.** Ok. **‘Cause this question, you asked it totally different. You said, ‘choose the right portion.’** Right. Ok.

**And again, you do that again here. A portion of rice… I’m gonna say a half a cup. Is there any reason that you don’t introduce a plate?** As in, the plate method? **Yeah.** Because we’re not educating people through this, we’re trying to figure out what people already know, which would be a step before educating. So, um, that would be an educational approach, um, but we want, we’re interested in knowing how familiar people are before they were… yeah.

**That is an awkward question.** Ok. How could we … that? **‘Which portion below…’ take out ‘below because we’re on question (mumbles). ‘Which portion of peanut butter is equal to the portion for one serving…’ ‘Which portion of peanut butter below is equal to one portion…’ um, ‘to the portion for one serving on the label at the right?’**

Ok, so I just want to ask quickly for that, for that section, that took us a little bit. Um, but you brought, gave us some good feedback, so how it was originally presented to you, if you again use that scale of one, ‘that was pretty easy’ to five, ‘that was pretty difficult,’ thinking back to how we started out, how would you rate that section? **Well, I’d say as far as the knowledge that you’re asking for it was a one.** Ok. **But as far as the communication in terms of interpretation and the understanding of the questions, I’d say it was probably a four.** Ok. So how we’re asking the questions and how we’re presenting those instructions, um… **Right. Those last few questions were perfect. I knew exactly what you’re trying to get to.** Ok. Great.

(Numeracy)

**Ok, is that five hundred.**

**Why do you say ‘cups’ instead of servings? Serving size is one cup. (reads question again) One.** Ok. It’s irritating to you that I ask about cups instead of servings? **Well, I couldn’t remember if, how many cups were in a serving. So, I had to go see…** That’s what we want you to do.

**Now I have to do math! Fifty-five… Fifty-three grams.** Ok.

**Sixty-two.**

**Well, so this is based on what I know.** Ok. Based on what you know, so, the label didn’t provide the information? **I, I didn’t get a sense of… Oh, there it is, dietary fiber, let’s see.**

**Basically two.**

**Six grams.**

**Ten.**

**So, fourteen.**

**(mumbling) percent intake… eighteen.**

**How’d I do, boss?** Great! How do you feel about math? **I have, you know, I’m so foreign to it. I don’t use it that much anymore, since I retired.** And how about reading food labels? Do you do that very often? **I do.** You do, ok. **Every time I shop. Well, I used to… now I know what I use.** So you don’t have to read it anymore? Ok. **But I, I think it’s, I think it’s good that they’re starting to get more clarity in the labels.** I just want to ask about… how difficult do you think this section was, on a scale of one to five, in understanding what was asked? **I’d say it was a one.** Ok, ok. And that’s, it sounds like you’re pretty familiar with reading food labels, so, yeah, some of that math, doing it in your head. **Well and I didn’t see the dietary fiber in there the first time, so I had to…**  Search for it a little bit. Ok. How about visualizing the label on the iPad? Was that ok? Was it hard to use it or…? **I had to keep going back and forth, and so that makes it hard to use. And I see what you did, you tried to drop a label, but maybe on a larger iPad it would work, but not on the small one.** Ok. Ok, well, we’re at an hour, so we probably need to stop here.

***Interview 5***

(Begin at food groups)

Apple: **Um, I would say fruits.**

Milk: **Um, dairy.**

Noodles: **Uh, I think it depends.** Ok, tell us what you’re thinking. **I would say grains, but it would depend on the noodles. And then on top of that, they said that noodles consist of sugar, so it could be between those two.** So you’re thinking between grains and added sugars? **Well, no… also ‘cause it’s considered, you can gain a lot of weight behind it, too, so I would say fats and oils and added sugar.** Ok. **Yeah, but if I had to pick one, I would say added sugar.** You would pick added sugar. Ok. Now, um, so you said it depends on the type of noodle. So, so what would you think of, you know, it would need to be specified as for you to maybe, you know, to choose, to look at that list and say, ‘I know it’s definitely grain’ or ‘I know it’s an added sugar?’ **It would have to have, um, ‘whole wheat’ on the package or, um, it would have to say it in the ingredients, you know, or it would have to be organic, something like that. ‘Cause I know the majority of organic foods, you know, they try not to have added sugars and stuff.** Ok, so it’s a little bit confusing that it’s just, that it just states ‘noodles’ because you can open into so many, quite a few food groups. **Yeah, ‘cause I know Ramen noodles is considered sugar, it’s a high-sugar grain. I know that they said that women should not, um, take part in that because, uh, it’s linked to, I think, diabetes and added sugars and… When you think about diabetes, that’s the one thing that... your body’s insulin and your blood sugar going up.** Ok. Good, good. That is, you’re doing exactly perfectly what we want you to do. So just keep doing along what you’re doing.

Orange juice: **It would be two, two different ones. Because, um, it depends, once again, on if it’s fresh-squeezed or if it’s like, uh, Sunny D. ‘Cause Sunny D, from what I’ve been told is not, um, actual orange juice. It’s like sugars and it’s not really even considered—even though I consider it real orange juice—so… but, uh, yeah. I would pick added sugars.** If it’s Sunny D or if if’s fresh-squeezed orange juice? **If it’s fresh-squeezed, I would pick fruits. If it’s Sunny D I would say added sugars.** Ok. So you need kind of a description for this one? ‘Which food group does fresh-squeezed orange juice…’ **I think if it said ‘fresh-squeezed’ I would say fruits.** Ok. What if it said ‘frozen?’ ‘Frozen concentrate?’ **Fruits and vegetables, because it’s actually, you’ll see the pulp in it. You can’t, you can’t, uh, what do you call that, uh, mock that. You can’t like, you know, like an adjective, like a flavor, you know, an artificial flavor, you can’t artificially add…** You can’t make it artificially? **Yeah.** Ok. Um, if it said, ‘one hundred percent orange juice?’ **I still would, if it’s not freezer, from fresh—** So if it said a hundred percent orange juice, that would make you choose fruit or added sugar? **Um, if it was on a Sunny D bottle and it said that?** If, just if you read this question, ‘Which food group does 100% orange juice belong to?’ **Oh, um, fruit.** Fruit? Ok. Ok, good, perfect.

Cheese: **Um, that still also could be a toss-up because, um, from what I understand Kraft is not one hundred percent, you know, a lot of these cheeses, a lot of these cheeses that they use, they’re not, they’re like, uh, things added to it.** You’re thinking like the Singles, the Kraft Singles? Ok. The American? American cheese? **Yeah.** Ok. **Like soft cheddar and stuff like that. Like you go to a deli, I’m guessing that that is, um, one hundred percent, so, I would say… I would say dairy, you know, but, um, if you put it in front of me and it wasn’t, like, in a block… um, if they had like three different descriptions like ‘shredded,’ Kraft, and the block, I would put the block in dairy and I would put the other ones in fats and oils and added sugar.** Fats and oils and added sugars? For the… So for the shredded and like the Kraft American sliced cheese, you’d put those in either the fats and oils or the added sugars. **Mmhmm. ‘Cause you can always shred a block yourself. (mumbles) I think that’s (?). The little block that you use?** The shredder? Yeah! Um, if it said ‘Which food group does cheddar cheese belong to?’ **If it’s a block, I would say dairy.** So it has to be a block or it needs to be shredded for you to think of…  **Even shredded, I would still…** Not shredded? **I’m not sure about shredded because it can still be stuff added to it.** Ok. **Yeah, you know, in order to be packaged and, um…** So with block, do you always think of the, the rectangular-shaped cheese that you can shred, or could you see where maybe some people would think of the block as like that Velveeta, the Mexican cheese? What would you… **Even a block of Velveeta, that can be stuff added to it, so, um, I would think like a, like if you go to Hy-Vee and you see, uh, where it still has the, the um, what is that, the shell around it, you know those kind of cheeses, I would consider those more fresh, you know, like not pre-packaged.** Ok, so you would classify those as…? **Fats and oils.** Fats and oils? Ok. **I mean, cause some cheeses can still be considered fats and oils, so… I guess I would put it in dairy, I don’t know, it’s kinda hard, because…** So depending on the type, you could see it as one of those three options? Dairy, those last three, dairy, fats and oils, or added sugars. Depending on what type we possibly specify. **Mmhmm, ‘cause I know that, like, to make like a certain… I don’t know, ‘cause macaroni and cheese, that’s considered a fat and oil too, so it’s like, it takes you to another…** So you think if it was specified, like Heather said, cheddar or mozzarella, it would still need to specify whether it was maybe blocked or shredded or sliced or… What if it said, like, ‘string cheese?’ ‘Mozzarella string cheese?’ **Probably dairy.** Or ‘fresh mozzarella cheese?’ **Yeah, if it said fresh, yeah.** You would consider that dairy? You would be clearer? **Mmhmm.** Ok. Ok, let’s go ahead and move on to the next one.

Rice: **I would say grains.** Ok. **My doctor would say added sugar. ‘Cause they just put me on a insulin-regulating pen and she, that’s one of the things that she told me that I have to cut out is rice.** Ok. **So I think, when you, when she does that and insulin, you would think sugars.** Is that regardless what type of rice, like say if it said ‘white’ or ‘brown’ rice in there. **She didn’t say (mumbles).** Any rice. Ok. **Um, but I think if it was someone else, if it said ‘brown rice,’ you would think grains.** Ok. **‘White rice,’ added sugar.** Ok. Ok, good.

Tortillas: **Um, it’s made with corn, so… oh, that’s hard. Uh, I would say grains.** Ok. Kinda tell us why you think grains there, just your thought process there. **‘Cause you have to add flour, I think, I’m guessing, to make tortillas. So, that you have to add flour—** And you said that it was made of corn, were you think of a corn tortilla, is that what came to mind? **Mmhmm.** Ok. Would it matter if it said corn tortilla or flour tortilla? **If it was wheat? I don’t think that’d make a difference.** It doesn’t matter? **Um…** Would you still assign it to grains? **Yeah.** Ok. **‘Cause it’s the only way, I think, to see that.** Ok. Process of elimination there, isn’t it? **Yeah.** Ok, good.

Chicken: **I would say protein.** Ok. **But also it depends on how you cook it.** Ok. So if we said baked chicken what would you say? **Protein.** Ok. Fried chicken? **Fats and oils.** What if we just said raw, um, ‘What food group does a piece of raw chicken, uncooked raw chicken…?’ **Proteins.** Ok.

Pork chop: **Um, I’ve been told that it’s considered ‘the other white meat’ and that it’s in the chicken department, so I would say protein.** Ok. Is that kind of like the chicken though, it depends on how it was cooked, you would… **Mmhmm. Because I noticed that, my mother, um, ‘cause we have high blood pressure in our family, me and my cousin have tried to opt for us—me, my mother, and him—to eat ours baked. And the kids, because they’re growing, we allow theirs to be, um, if they want to try the baked one we allow them to, but, um, for now, um, and, um, I guess the baked is supposed to be more healthier for you.** What if it said grilled? **Um, I think that would be a plus on the protein part.** Ok. **Like a Forman grill or something like that, yeah.** Ok, good.

Carrots: **Um, it’s a vegetable, but it also depends if you cook it or not because you, from what I understand, when you cook it, it loses what it’s supposed to have. Um, and it’s also a sugar.** Ok, so if we said raw carrots? **Raw carrots, it would be a vegetable, but also a sugar, ‘cause from what I understand it, it, um, has a lot of sugar in it, it has a natural sugar. Well, not it’s not… Yeah, it would be a vegetable ‘cause you, ‘cause this said ‘added sugar,’ so, yeah.** Ok. What if we said cooked carrots? **Um, I guess still vegetable.** Vegetable? Ok. So do you think it needs ‘raw’ or ‘cooked’ in front of it or you would assign it a vegetable either way? **Either way, ‘cause it, ‘cause it’s not an added sugar, ‘cause it’s natural.** Ok.

Butter: **Fats and oils.** That one was an easy one. **Well, it can be dairy too ‘cause they said, um, when you churn it, like if you did it, I guess they said churn is totally different from the way we eat it, like on a farm. I’ve never had it that way, so, um, it could be a diary and it also could be a fat and oil, it depends on how…** Do you think of dairy because it’s made on a dairy or you get it from the dairy case at the grocery store, or what makes you, what makes dairy come to mind? **‘Cause I, um, I thought it was made with milk. That’s what I thought.** Ok. **But that’s the one thing that comes to mind.** If it was churned, if it said churned on there? **Mmhmm.** Or just regardless? **If it said churned, home-churned, something like that, that’s one that I would think of.** You would think dairy? Ok. **Yeah. Because a lot of things say butter, one hundred percent butter and it’s not, ‘cause… yeah…** Ok.

Fruit punch: **Added sugar.** Ok. And when we say ‘fruit punch,’ what does that mean to you? **Uh…** Is there a certain brand that comes to mind or…? Something that you’re visualizing? Yeah. **Kool-aid.** Ok! **I don’t drink it, but that’s the first thing, they say ‘fruit punch,’ that’s the first thing…** Ok.

Spinach: **Um, vegetables.**

Bananas: **Um, it has a natural sugar in it, but it’s not added, so I would say fruits and vegetables. ‘Cause I was told that, like, when you about to do a blood sugar test, if you eat a banana, uh, it will elevate your blood sugar. So it would give a false diabetic reading.**

Salad dressing: **Uh, I was told that they use oils … Fats and oils.**

Lemonade: **If it’s homemade, I would say fruits.** Ok. **If it’s bought from the store in a jug, I would say added sugar. Well, you gotta add sugar to it at home, so… But I think, out of the store in a jug that it’s more artificial sweeteners put into it than, you know, you would at home.** So as it is written right now, which one would you assign it to? **Uh, fruits.** You would assign it to fruit? **Mmhmm.** So when you read ‘lemonade’ you think automatically ‘fresh-squeezed lemonade.’ **Yeah, ‘cause you can make it yourself or… but if you bought it from the store, if they say ‘store-bought lemonade—‘** Then you would automatically assign it to added sugars then? **Mmhmm.** Ok. Would you say the same thing, then, about the orange juice, too? Homemade versus store-bought? **Mmhmm.** What about fresh-made at a restaurant? **Still, ‘cause I can’t see them.** You think it’s added sugars? **Mmhmm.** Ok. **If I can’t see the person making it, it’s, it’s basically, probably, you know, they could easily go get it out of a refrigerator and just pour it.**

Ok, so, overall, on a scale of one to five, with one being ‘that was pretty easy for me,’ to five being ‘that was pretty difficult,’ how would you rate that section that you just completed and why? **Um, it was, it was on the easy side, um, because it’s like my ideals on it, my spin on it and how I view, you know, um, and I think of… Um, I think it depends on the person.** Ok. **Because, me, I have a controlling problem, uh, where some people don’t have a controlling problem, they just go with the wind, you know, they would, you know, probably just say, you know, ‘oh, the dairy group,’ you know.** So you focus on the details, you mean? **Yeah.** Ok. The thought process. **Yeah.** You really did good on though process throughout that. Um, were there any foods that were unfamiliar or were there any questions that you thought, ‘you know, this maybe doesn’t belong here?’ **Um, no.** Ok. So the foods are pretty familiar and the questions, none of them seemed out of place or confusing? **No.** Ok, pretty straightforward? Good!

(Consumer skills)

Let me ask you this before you get started, before you read the directions: are you the primary person in your house that shops for foods? **Mmhmm.** Ok, ok, and are you the primary person that also prepares the foods? **Mmhmm.** Ok, great, great! **I do not trust my sons wash my dishes. If they wash my dishes, I will go back behind them and wash them myself, I don’t trust them. I don’t even trust my mother to do them. … … … … …** So go ahead and just read us the directions and just go ahead exactly like you’ve been doing, you’ve been doing perfectly.

One: **Uh, I would say C.** Ok.

Two: **Uh, I would say raisins.** Raisins, ok. Why would you say the raisins? **‘Cause the, they say that those are made from, I mean, they’re not, like, I guess they’re supposed to be like an imitation, um, dehydrated food, but they look like raisins, but they’re different colors. So that would make me think that that’s something that was made and just put into the colors so you would think that’s what it’s supposed to be, like, the yellow is supposed to be a pear, the red is supposed to be an apple, you know, orange is supposed to be an orange, and you can’t dehydrate those to look like these would.** Ok, so it’s misleading? **Yes. For a kid, they would be like, ‘oh yeah!’ but for an adult, no.** Ok.

3: **Um, I would say they’re equal, ‘cause they’re both beef.** Ok. **I’m not a steak person.** Ok, so these are not foods that you typically prepare? **Yeah, like a patio steak, um, I have made a T-bone steak before for, like, Father’s Day for my cousin, but not on the regular, I don’t.** Ok. **I’m more of a chicken person.** Ok.

4: **I would say milk.** Ok. And do you purchase both of these beverages? **Um, well my son is, uh, allergic to milk, but he’s not allergic where he can’t have, like, a little bit. And, um, if I, being that he plays basketball, I would give him the milk before I give him the juice. Well, no, because him playing that would, um, make his allergies run through his body quicker, so… if he had no milk allergies, I would say milk.**

**5: The frozen.** Ok. You chose that pretty quickly. **Um, because I did a nutrition class (?) and I learned that, uh, that canned has more sodium ‘cause it’s been canned and it’s been sitting longer, and you have to rinse it like twice, and uh then you have to steam it in water and rinse it again…** Quite the process, huh? **Yeah. So you’re better off just doing frozen, you know.** Sounds like they would taste like nothing after that. **Yeah. So you might as well just go with the frozen ones that have no flavor to it to begin with and just add your own flavors.**

**6: Um, I would say the frozen red.** Ok. And why would you pick the frozen red? **I was told that red-skinned potatoes are better for you, you know, you put them with roast, pot roast, um, for some reason. ‘Cause I’ve never seen nobody use the second picture with roast. And, uh, they said that the skin is good for you, but I think it depends on the potato that you use on top of that.** Ok. And what comes to mind when you’re reading the question, ‘best nutrition,’ so ‘which food provides the best nutrition?’ So, what does that make you think of when you’re looking at the two of them and comparing? **Uh, comparing on, I think, how you would prepare them and what the best use for them is. With the second picture you would think, uh, you would think either fries or you would think mashed potatoes.** Ok. **The red ones you would think pot roast, um, brisket, stuff like that.** Ok, so you have images in your, of your mind… **Of how to prepare them.** Ok, how these different potatoes are prepared. Ok.

7: **I’ve never had kale, but, um, I’ve been told that, uh, you know, fry it in peanut oil and it tastes like a chip, it’s supposed to be nutritious for a kid. Um, so, I would say the kale. I’m used to the Iceberg, ‘cause that’s what I grew up with, but I’ve seen that kale is being used in everything now—nutrition drinks, they fry them, you can bake them.** But kale is unfamiliar for you? **Yeah, it’s unfamiliar, but I’ve seen a lot of it.** You haven’t eaten it yourself but you’ve seen a lot about it? **Mmhmm.** Ok. **It looks expensive too.** Have you seen kale at the grocery store? **Um, the new Walmart they put in, but in, um, Argentine, Kansas, I believe I’ve seen some there. Um, well, they have a lot of, um, different stuff than the other Walmart’s do. Um, like, I guess the further you come this way, you see organic stuff more. Um, I think it depends on where you live at and what part of the city you live in.** Do you think something like spinach as an alternative to kale would be a better alternative in there, or do you think it matters? **Yeah, I think if you compare kale and spinach, it would be, like, equal. ‘Cause I’ve, um, thought of, um, ‘cause my son he participates in the Harvesters Food Bank think and they gave these little containers with fresh spinach in it and I didn’t get around to, I was gonna cook it like you do greens, and, uh, I used turkey bacon in there so that I wouldn’t have to add salt to it. Um, turkey bacon basically adds your own flavor to, like, if you’re making greens for, um, Thanksgiving and, um, they expired before I got a chance to do that, so, yeah. But I was told that fresh spinach is better and it’s, like, I guess it’s the same nutrition as kale, but it’s less expensive.**

**8: The Nutrition Facts panel. ‘Cause my sons, they would go for the second one. They be like, ‘Mama it says no sugar!’ Yeah, ok.** Do you read that Nutrition Facts panel, do you look at it? **It depends on what it is. Sometimes I do. But then it also depends on the price too. I try to improvise a lot of things.**

**9: I’ve tried Naked before and it tastes like it’s fresh. So I would say they’re equal.** Ok, so you’re familiar with that brand. **Yes. I went to Rite (?) up on Metcalf and it was a Hy-Vee right next door to us, so I got to try it one day and, yeah.** And it tasted like fresh-squeezed? **I didn’t get that far. I had to end up giving it away.** Oh! You didn’t like it? **I guess I picked the wrong one that day or something. I think I picked, like, strawberry-banana or something like that. I should have picked something that was, like, kiwi-strawberry or something like that. But the girl that was in my class she was a “health freak” and she swore by them and she downed it like it was nothing. So, yeah, I would say they’re equal.**

**10: The ingredients list.** Ok. **‘Cause they can say it’s whole grain, but then when you read it and it has all this other added stuff to it…** So in both questions, eight and ten, it asks ‘provides the best information.’ What’s that mean to you, ‘best information?’ **Um, more detail.** Ok. **‘Cause when it says ‘whole grain,’ it’s just, the list should be shorter. If it’s artificial, it’s gonna have more ingredients to it. Now, it depends on, like if it has, I mean, vitamins in it, B-six, B-twelve, like that, then I would, the more that’s there then I would go with that. But if it said it’s soy and corn syrup and fructose and all that, I’m not gonna buy that, even if it said ‘whole wheat.’ That means it’s, it’s a lot.** Ok. So, for ‘best information,’ that’s true and more detailed? **Mmhmm, yeah.** Do you ever look at the ingredients list? Do you have time? **Um, it depends on what it is. Like, um…** Like say if you did see a package that said ‘whole wheat’ on it. Would you flip it over and look at the ingredients list? **Mmhmm. Like, if I’m buying muffins, whole wheat muffins or my nephew likes bagels. Stuff like that I would, because, um, for myself because now I’m older and my doctor’s telling me certain things I can’t have. I would look at it. But for my nephew, he pretty much eats everything and anything. You know, and whether it’s nutritious or not he, I mean, he loves fruits and vegetables, I mean, broccoli, everything.**

On that, so for that section, again, let’s go back to that scale of one to five, one is “that was pretty easy,” to five “that was pretty difficult.” Um, how would you rate that section? **Probably a three.** Ok, a three. So that was the one, you felt that one was a little bit more difficult than, um, the food groups section? **Um, it was more in depth.** More in depth? **Yeah.** Any foods that were unfamiliar to you? Um, I know we talked a little bit about the kale, you’re familiar with it, just had never had it before. **There’s this show, uh, me and my younger son watch, uh, Master Chef and they had the junior one and one of the kids was cooking kale. And he was frying it and it made me, like, ‘ok, you can really, if a kid is cooking this,’ you know.** Can’t be too bad? **Yeah! So it’s… yeah.**

**1: Um, ‘cause I was told that it’s a vitamin E. But butter is considered fat, too. Uh, I would say fat.** Ok. Why did you decide on fat instead of vitamin E? **Because of butter. ‘Cause if you’re cooking these things together, it’s the butter is going to (?) the olive oil.** If it just said ‘olive oil,’ what would you have chosen? **Uh, vitamin E.** Vitamin E? So when you read that question, um, you’re thinking they’re, um, if you’re cooking in both of those together, both of those items are being used together? **Mmhmm. I would think the butter would overpower…** Ok.

2: **Uh, I would say sugar.** Ok. And why did you pick sugar in that list? **Um, ‘cause it said ‘carb.’** Ok. So when you look at the rest of the items on the list, were you just eliminating them? **Mmhmm.** Ok. Because they didn’t make you think of carbohydrate? **Yeah, the calcium…** Ok. **Or protein.** Ok.

3: **Uh, protein.** Ok. **‘Cause when you hear about chicken and fish you see protein, and so eggs would have to go along with that.** Ok. If it had just said eggs what would you have picked? **Um, probably carbs.** Carbs?

4: **Um, protein, protein. I would say C.** Ok. Why did you choose C out of all of the others? **Um, the pork chop and the egg.** Ok. Were there others you were considering? **Uh, I was thinking about the banana, applesauce, and broccoli.** Ok. For the protein? Ok. So what you made you choose, so, the pork chop and the egg, made you choose that one over maybe the choice B. **Mmhmm. Because the egg is considered, in the class, uh, chicken, and the pork chop was supposed to be considered, uh, another form of chicken.** Ok. **So… and they said chicken is supposed to be protein.** Ok.

5: **Um, carbs, carbs. I would say B.** B? Ok, what made you, just kind of tell us your thought process as to why you eliminated the other answers and ended up with that choice. **Because of the sugar.** Because of the sugars… Ok. Because that choice had more sugar? **I would, I would, yeah. Because the peanut butter, they’re gonna add some kind of sugar to it. The cheese, sugar.** Ok. **And the egg, um, it’s gonna have some somewhere in there, I think it would.** Ok.

6: **Uh, D.** Ok. And again, just tell us your thought process, why you chose that one over the others. **Margarine, saturated fat. Almonds, I was told, are a healthy fat, um, and they also, like if you’re constipated, I didn’t know if they were considered a laxative.** Ok, good.

7: **I would say A.** Ok. Tell us why. **‘Cause the toast alone, that’s, that’s carbs. And the jam, strawberry jam is gonna have artificial sugar in there.** Ok. **And then the orange juice has a lot of sugar in it too.** Ok. **Yeah.**

**8: Salmon.** That one you arrived to pretty quickly. **Yeah, um, I, uh, I tried, uh, the real salmon before. It’s, it’s actually really good. Um, um, and it’s supposed to have omega-threes in it. And, um, there’s another thing, it’s a fat, it’s another fat that’s in there I think. But it’s supposed to be more healthy for you. And it’s a fish.** So when you read ‘healthy fats,’ that’s something that makes sense to you? **Mmhmm.** Ok. And you thought of the omega-threes? **Mmhmm. My mother, she, um, I buy her omega-three tablets from, um, Dollar General, and she takes those, um, along with her blood pressure medicines.** Ok, good.

9: **I would say black beans. ‘Cause everything else is basically what my doctor’s trying to get me to stop eating.** Ok. **‘Cause regular salad dressing on top of that is a lot of fat and sugar.** So you would say, you would choose black beans over the rest of them? **Mmhmm. Oh, you’re, wait a minute!** Yeah, I think you might have misread it a little bit. That’s ok! **Uh, I would say salad dressing.** Ok, salad… ok, is there a way that we worded that that was confusing? **No, I just…** Ok, ok, just making sure that’s not a way, that, some words that we maybe need to… **Yeah.** Ok, but you would choose salad dressing. **Yeah. Yeah, cause it, it has a lot of sugar and fat, so yeah.**

**10: D. It’s a healthy fat, but if you cook that with butter, like earlier, it is no point in even putting the olive oil in there. Unless you just like the flavor of olive oil.** Ok, good.

***Interview 6***

[after first explanation of nutrient and energy dense] Ok, stop right there. Do you have any children? **Yes.** Ok. How would you rephrase that information if you were wanting your, to teach your child about what you just read? What would you say to them in your own words? **Um, that’s hard. All of that information?** Yeah, you know, just kind of paraphrase it in your own words, what you would say to them, basically. You know, in two or three sentences. **Ok. Um, well I would probably say that eating a healthy diet helps, um, helps you be more healthy. And, um, let’s see, I’m trying to…** How old are your kids? **Two, no, three, nine, and twelve. Ten.** Ok. So if you were saying it to your twelve-year-old. **Oh, ok, I was trying to teach my three-year-old.** That’s ok! So let’s say your twelve-year-old. **Ok, my twelve-year-old. That’s easier. Ok. So, yeah, eating a healthy diet can improve, um, well, help prevent diabetes and high blood pressure, and it also helps you maintain your weight.** Ok. Good. What would your twelve-year-old think of when—it’s a girl?—what would she think of when she heard those words, ‘healthy diet’ together. **Um, she’d probably wonder what a healthy diet is. Or what it consists of.** Ok, so… ok. And so, that second paragraph, then, about nutrient-dense foods, is that a new term? Or is that a familiar term? **Probably new. For her? Or for me?** For you. **Not for me, but it would be for her.** Alright. And so, the phrase ‘healthy diet,’ does that make you think of something good, negative? Is that a positive phrase or a negative phrase? **I’d say positive.** Ok. Ok, and so you don’t think your twelve-year-old daughter, too, she would want to know what a healthy diet is but what, maybe associate that with something negative or… ok. **Negative, maybe in the sense that you have to change what you’re eating in order to have a healthy diet.** Ok. Do you think that would be her response, like, ‘oh, I don’t want to be on a healthy diet.’ **Um…** Didn’t come to mind when you were thinking about it? **No.** Ok, that’s all we were wanting to know. Ok, you’re doing great, go ahead and read on. [continues reading; stop after nutrient-dense foods] So how would you describe a nutrient-dense food to your oldest daughter? **I would just say fruits and vegetables.** Ok. **I don’t think she’d know what plant foods are.** Ok. **And I honestly don’t know that I know what, well, like lettuce and stuff like that, I guess. But ‘plant foods’ is maybe kind of…** An odd term? **An odd term, yeah.** What would you put in there in place of ‘plant foods?’ **Well…** We’re talking about grains and, um, not just fruits and vegetables, but things that are made from grains, too. **Yeah. Maybe just ‘whole-grain foods?’** Ok. [continues reading; stop after energy-dense foods but before refined grains, sodium, saturated fat, etc.] Ok, um, so, there was some tripping up on that paragraph. Was there some things maybe that are worded, um, confusing, or I guess maybe how would you explain that paragraph, how would you paraphrase that, that paragraph to your daughter, maybe? How would you explain ‘energy-dense’ to her? **I would say, I guess, um, I would say that the foods, like the chips, sodas, and things like that, are high in calories but low in healthful nutrients.** Ok. If you were explaining it to her? **Yeah.** Ok. **That’s what kind of confused me. I expected it to, if it’s high in calories “and” I thought it would be something that goes, that’s good instead of bad.** Oh, low in healthful nutrients? **Yeah.** Ok. So you had said, ‘but low in healthful nutrients;’ you think that would be better? **My thinking, yeah.** Ok. **The way I think.** Ok. And that would help to clarify? **Yeah.** Ok. Is ‘energy-dense’ an unfamiliar term? **Yeah.** Ok. So, how do you, now that you’ve read that, what does energy-dense mean to you? **Um, um, well like a, I’d say high fat.** Ok. Ok, go ahead. [continues reading; stops after refined grains] Ok, any problems with that one, or do you think, how would you explain that to your daughter? **So, um, there are grains that lack the healthy part of the grain. Well, what’s the healthy part of the grain?** You’re wanting to know more? **Yeah.** Ok. Ok, so you think that information would be helpful in there? **Mmhmm.** So if we put, ‘known as the bran?’ Would that make it too complicated, or… **Known as the brand?** Bran. **Oh, bran! Ok.** Or, ‘these foods are made from grains that lack the bran, the healthy part of the grain.’ **Yeah.** Ok. And you don’t think that would be too much information in there that people might want to know that? **I think it would help explain or help understand why refined grains are not as good as… because people think, ‘well yeah, grains, that’s what I need.’** Right. Ok. That grains are necessary? Ok. **And then, maybe, um, bran, if you put ‘bran’ in there, maybe people aren’t gonna know what bran is.** Right. So you think that could be confusing? **Mmhmm. Though maybe if you put that in there, maybe you would have to also elaborate on what bran is.** Which would be too much information at that point, wouldn’t it? **Probably.** Ok. **So maybe not…** So maybe not put ‘bran?’ This is a dilemma we definitely struggle with. **Is there any foods that… hmm…** Is there a different way that maybe we could phrase especially that first sentence? **Yeah, maybe that’s all it needs.** Ok, so what would you maybe suggest? Is the word ‘grain’ in there maybe too much to make it confusing? Or… what… do you have any, it’s ok if you don’t have a suggestion. Is that what’s…? **These foods maybe, you could just say they don’t contain the healthy part of the grain?** Ok. **That is essential and why that makes a difference? Maybe?** These foods do not contain the healthy part of the grain. **Or you could just say, ‘these foods lack the healthy part of the grain.’** Ok. **And that would make sense. Refined grains, they take out that part and just leave the crap.** Ok, ok. **Which makes, that’s why it makes them, why too many can lead to weight gain. That would make more sense, I guess.** Ok. So do you think it should say, ‘these foods lack bran, a healthy part of the grain?’ Or should it be, ‘these foods lack a healthy part of the grain,’ and that would be enough for people? **Yeah, I think that would fit.** Without ‘bran?’ **Yeah, without ‘bran,’ because then they’re gonna think, ‘well what’s the bran?’** Yeah. Right. Ok. Ok, good, thank you. [continues reading; stops after sodium] Ok, when you think of shelf-stable, is that a term you’re familiar with? **Um, I’ve never heard it phrased like that, but I understand what it means.** Ok, so— What are some examples of foods that are shelf-stable? **Um, like canned goods.** Ok. **Or, yeah, canned goods.** Ok, how else would you maybe phrase that? Um, or how else have you heard it phrased that would make it more familiar? It’s ok if you can’t… **What is the word?** Ok, it’s just that it’s self-explanatory? **Yeah, I, I understand it.** Ok, great. [continues reading; stops after added sugar] Ok, how would you, so reading through all of that, if we just let you read through all of it without interrupting you, would you say that, on a scale of one to five, with one being ‘that was pretty easy’ to reading it through and I learned some things, to five being, ‘it tripped me up and I don’t know, I didn’t learn very much,’ what would you, how would you rate that on that scale of one to five? **Um, I would say probably a two.** Ok. **It was pretty easy to understand.** Pretty easy to understand? Ok, good.

1: **Foods such as whole grains should be included often in a healthy diet.** Ok. That was a pretty quick answer? You didn’t have to debate too much? Ok. **Not from the choices there.**

2: **Raw carrot sticks? Just by elimination.** Ok. So why did you choose raw carrot sticks? **Um, well, I remember energy-dense foods is, uh, oh, wait, no! Oops.** That’s ok. **I was thinking of… where was that one that said, uh, I was thinking, um, plant foods. Nutrient-dense foods are plant foods.** Ok. **So the energy-dense food would be ice cream.** Ok. Those terms kind of trip you up a little bit, the energy-dense versus the nutrient-dense? **Yeah. Well, energy-dense foods you would think is good because it gives you energy.** The word energy is in it? So that’s kind of confusing? **Yeah. I would think of ‘energy’ as good.** We had someone else say that too. ‘Energy’ seems like it would be considered positive. **Yeah. Like a food that would give you energy.** Ok.

3: **Um, an orange.** Ok.

4: **Cholesterol.** Ok.

5: **Broccoli is nutrient-dense.** Ok.

6: **Animal-based.**

**7: Ok, nutrient-dense. Probably C, three ounce chicken, one cup steamed green beans, low-fat milk.** And why did you choose that one? **Um, I’m thinking nutrient-dense is the best for you and what you should have more often. And vegetables, definitely one, and skinless chicken, because it’s skinless, and low-fat milk.** Ok. **I’d say that one’s the better one.** Ok.

8: **Now, from what I’ve read, I’d say blood pressure. But I could choose more than one on that one. Because cholesterol has to do with the heart too.** Ok.

9: **I’d say definitely chocolate pudding.**

**10: Um, energy-dense, that’s the bad one. So, probably lemonade.** Ok. Why would you choose that over something like coffee or tea that have caffeine? **Um, because energy-dense is like sugars.** Ok, good. **Instead of… diet soda doesn’t have any sugar, neither does black coffee or unsweetened tea, so lemonade, there it is.** Ok. And when you think of black coffee, what comes to mind? **Nothing in it. Just coffee.** Ok.

11: **Uh, canned soup, canned tomato soup.**

Ok, great, you’re doing everything perfectly, just like we asked you to. Um, I want to ask you about that section, let’s go back to that scale of one to five, with one ‘this was pretty easy,’ to five ‘this was kinda hard.’ Um, how would you rate this section overall? **Um, probably about a three.** Three? So right there in the middle? **Yeah.** Ok, so was it because there were certain questions that tripped you up or were confusing or some that were maybe listed that were confusing? **Um, the questions, more on the questions than the…** Ok, any of those questions that you’re thinking about, are there any that you can point us to that were more… **Uh, the energy, the energy-dense. Yeah. Because somebody just doing this on their own without having any feedback, the would probably miss all the energy-dense food questions. I guess if they were able to go back up there, then they would probably…** So you think that it would, it’s important to be able to answer the questions that you have the text to read beforehand? **Yeah. I think if people were not able to go back, they would probably likely not remember that energy-dense is bad.** Ok. Good. Great.

1: **Fat.** Ok.

2: So what are you thinking as you look over those? **Well, I guess I don’t know. ‘Cause I don’t know what this answer is, um, so fresh-squeezed orange juice… sugar?** Ok, and what made you pick that one? **Um, because, thinking about fresh-squeezed orange juice, the first thing that comes to the mind is you get sugar from juice.** Ok. **I guess.**

**3: I would choose protein.** Ok.

4: **Um, I would say ‘pork chop, egg, cheese.’** Ok.

5: **Carbohydrate… Um… probably… ‘oatmeal, potato, milk.’** Ok, and what made you choose that one over the other selections? **I think of carbohydrate as, like, breads and, um, when I think of a carb I think of that.** So was there something in the other answers or that answer that made it hard to choose? **Um, no not really.** Oh, ok. **Now that I look at it.** Just taking time to read through? Processing it? **Yeah, processing it, yeah.** Good.

6: **Um, I’d say ‘mayonnaise, margarine, and almonds.’**

**7: I would say ‘eight ounce orange juice, two slices of toast with strawberry jam.’ Again, carb—bread.**

**8: I’d say salmon.** So the ‘healthy fat,’ does that make sense? **Yeah.** Ok. **It’s easy because salmon is just known as a healthy fish.** Ok. **A healthy choice. I don’t know. Yeah.** Ok.

9: **I would say regular salad dressing.** Ok. **It’s more fat.**

**10: Um, I would say it has more healthful types of fat.** Ok. What, tell us, kind of explain to us why you chose that one over the other choices. **Um, well, because I think, I’m thinking olive oil is not good for you by all means, um, but it is better for you than regular old margarine. Um…** A better choice? **Yeah, a better choice. If you had to cook with one, olive oil is the better choice.**

Alright, so for that section, let’s go back to that scale of one to five. How would you rate that section? One, pretty easy; five, that was pretty difficult? **I’d say about a two.** About a two? So that means easier? Any of the questions that were more, any more difficult or confusing? Any choices that didn’t make sense, maybe? No? **I’ve never really run across the word ‘healthful.’** The word ‘healthful?’ Ok. **In asking which is more healthy.** Ok. So you like ‘healthy’ better than ‘healthful?’ Sometimes it’s just simple things. Ok, alright.

So, when you read those instructions, what are you thinking you’re going to do? **Um, look at a picture and choose what’s best.** Ok.

1: **I would say about right for one portion.** Ok. How much did you look at the picture for that, did you look at the picture at all for that one? **I mean I glanced at it but I don’t need the picture.** Ok. The amount is enough for you to be able to answer? **Yeah.** Ok.

2: **It’s more than one portion.** Ok. Again, did you look at the picture or did you look at the amount? **(?) but again, I didn’t need to look at it to figure out… just because I already know.** Ok. So the text is enough information? **Yeah.**

**3: It’s about right. Well… yeah, about right for one portion.** Ok.

4: **About right.**

**5: Um, I think that’s more than one portion.** And those questions that talk about the pasta noodles and the rice, um, would you automatically assume that it’s cooked, or do you think that’s something that should be added in there? When you read that, did you just consider the text-- **I did not consider if it was cooked or uncooked.** Did you look at the picture along with it when you were reading the text? **Yeah.** Ok, and so by looking at, glancing at the picture, can you tell? **Yeah. Well, I mean you can tell that the rice is cooked.** Ok. **Not necessarily the pasta, I guess. If you want (mumbles). I mean it looks like it’s cooked, but… Overanalyzing, I guess.** Right. We’re forcing you to overanalyze it, so… Would that be something that should be added or do you think when people read these questions they’re gonna automatically assume, you know…? **Um, probably automatically assume. But it would make, um, it would make a difference if you put in there ‘cooked’ or ‘not.’** It would make it clearer **It would, um, it may change the people’s answers.** Ok.

6: **Ah, black beans. Um, I think that’s about right for one portion.** Ok.

7: **It’s about right for one portion, I’d say.** Ok.

8: **It’s about right for one portion.**

**9: It should be three ounces.** Ok.

10: **I’d say one half cup.**

**11: Two table spoons in the Nutrition Facts. So, B, two tablespoons.**

So for those questions, did you mostly rely on the, the text overall but you just briefly glanced at the, at the pictures? **Mmhmm.** Um, how much did the pictures help you? **I didn’t really need to use them unless I had to look at the pictures for the question.** When you think of the word ‘portion,’ what does that mean to you? **Um, the size of, or the amount of food that’s on your plate.** The amount of food that’s on your plate? If we use the word ‘serving,’ would it make any difference in how you understand the description? **No, I don’t think it would.** No? Ok. Ok, so for that section, let’s use that scale again of one to five. How would you rate it? **Uh, a one.** One? So that one was pretty easy for you? **Yeah.** Have you had, like, a lot of nutrition education on portion sizes? **Yeah.** Ok.

1: **If you eat the whole container, you would eat five hundred calories.** Ok. **Adding, there’s two, two serving sizes, one serving size per … two-fifty.** Ok.

2: **Fewer than five hundred milligrams of sodium… Sodium is 470, so one cup.**

**3: Sixty-five total… one cup… sodium, right? Hold on, fat. Total fat. (whispering) Fifty-three grams.** Ok. And how did you decide? **So, sixty-five grams per day, and one cup of macaroni, there’s twelve grams of fat in the macaroni, one cup. So, sixty-five minus twelve gives you what you have left.** Ok.

4: **Two cups… carbohydrate… um, thirty-one grams carbohydrate total, so that is sixty-two grams.** Ok.

5: **I’d say no. ‘Cause there’s zero dietary fiber in macaroni and cheese.** Ok.

6: **Saturated fat, seven grams. Saturated fat is three grams over here… So you could have two cups. Because three plus three is six.** Ok. Good. We’ve got you doing some math today! **Good thing it’s easy math!**

**7: Oh, one half cup, ok. One half cup would be six grams.** Ok.

8: **I’d choose ten.** Ok.

9: **Forty-five grams of carbs per meal. (whispering) So, carbohydrate, there’s thirty-one grams. Forty-five minus thirty-one is, uh, fourteen. Fourteen grams.** Ok.

10: **(whispering) Oh, eighteen percent.** Ok. Eighteen because…? **Um, ‘cause the total fat is eighteen percent in the macaroni and cheese.** Ok, for one serving? **Yes.**

Ok, so let’s use that same scale, one to five. **Um, that was pretty easy, about a one.** Pretty easy? Aside from the, the math that we had you do? About a one? Ok. **Yeah.** So, are you familiar with reading food labels? **Mmhmm.** Do you read them very often when you go shopping? **Um, not as often as I should, but…** When you have time? **Yeah.** Ok. Any, um, particular questions there that were confusing or didn’t seem to make sense? **Um, not confusing, just had to read more than once the last couple questions, just because of the complexity.** Yeah. And then referring back to the food label? **Mmhmm.** Ok.

***Interview 7***

Portion sizes

Ok, stop right there. Can you just kind of tell us what you think those directions mean before you go ahead and go on to the questions? What are we asking to do there? **Uh, you mean, like, the strategy of the question?** Yeah. **Well, you want me to, you want to see if I understand what portions are.** Ok, exactly. And what do ‘portions’ mean to you? When we say the word ‘portion,’ what does that mean? **Uh, how much. Amount.** Ok. Great, thank you. Go ahead.

1: **Well, I would say that it, uh, it’s about right, about one portion.** Ok, and when you answered that, did you look at both the picture and the text or did you rely more on one than the other? **Um…** Or, or hard to say right now? **Well, I guess it’s hard to say, I mean, I guess I used the picture and the words at the same time.** Ok. Well, just let us know, you know, as you go on if you, if you think that you’re looking more at the, if you’re just thinking about the text more or the picture more. **I’m just thinking about my own experience, I mean, I usually don’t have more than eight ounces of milk at one time when I have it.** Ok.

2: **I guess it’s, it’s probably about right, but it’s not, I would like more.** Ok! We have a lot of people say that. **Uh, it looks pretty skimpy in the picture. Yeah. But I would bet, I would guess that’s about the right portion.** Ok. Great.

3: **I think it’s less than one portion.** Ok.

4: **I would say it’s about right.**

**5: I would say less than one portion.** Ok. When we, when you see the, the, when we ask about, especially about the rice and the pasta, do you automatically assume that we’re talking about cooked pasta? **Yeah. And I don’t know, I can’t help keeping my feeling, my own, like if I went to an Italian restaurant and that’s all I had, I would feel, it would seem skimpy to me.** Ok. **Not that I’m that big an eater, but, um, I guess it’s the notion of defining things in cups. That, that, ‘cause I don’t think that way when I dish things up.** Do you use measuring cups? **Cups seem small to me. The idea of a cup.** Ok. Do you use measuring cups yourself? **Uh, only when I’m making something; not for food.** Following a recipe? **Not to serve food.** Ok.

6: **This is less than one portion to me.** Ok. **It makes me think of, you know that (?) that used to be on the Today show? They used to have nutritionists come in when he was on and he would always, you know, have fun with her, but she would be, ‘this is what you really should eat.’ And they were talking about Mexican food, which I love—I’m from southern California—and she said, ‘now, for instance, when you go to a Mexican restaurant, they’re gonna put that big basket of chips there, now just have two or three of those chips,’ you know, and then have some water. And he looked at her and he started laughing and he said, ‘I have two or three baskets when I go eat Mexican!’ That’s running through my mind as I read through this. It’s like, yeah, half a cup of beans, are you kidding?** I think that’s common for a lot of people. **Yeah.**

**7: I’d say less.** Ok. **Yeah. I mean a half a cup is about that much.**

**8: Now you’re talking about weights here. I don’t, um, I would guess, since I don’t really eat salmon, I guess it’s about right.** Ok. So you feel like you’re guessing on that one? **Yeah.** Ok. Um, and so… **Size is harder for me than, I can picture a cup, but the size… weight is a…** It’s harder to picture? **Mmhmm. Yeah.** So when, did you look at the picture at all to help you? Thinking that that doesn’t help? **Yeah, uh, well, I guess. I mean, pictures are relative, you know. The rice makes it look smaller.** So you’re kind of looking at the whole plate itself in comparison with all the foods, not just the food mentioned alone? **Yeah.** Ok. **And the parsley there is kind of a, I mean, what does that do? You’re not going to eat that. Maybe trying to fill up the plate, just presentation.** That’s right, presentation. Exactly.

9: **I would say, uh, five ounces. And I have kind of a sense for that from Quarter-Pounder and things like that, so…**

**10: Of those, I’d say C, one cup.** Ok.

11: **Well, it says two tablespoons, is that what you want me to know?** Mmhmm. **Yeah, two tablespoons.** Ok.

Now, um, real quick before we move on to the next section. Think about those instructions that we asked you at the very beginning, the directions that you read. Do they fit what we just asked you to do? **Mmhmm, I think so.** Ok, now for this section, if you were to think of a scale of one to five, with one being ‘that was pretty easy,’ to five being ‘that was kind of difficult,’ how would you rate overall how you felt about it. **I would say one.** A one? So pretty easy to do? **Yeah.** Were there any questions in there that didn’t seem to make sense or any foods that we asked about that were unfamiliar to you? **No.** Ok.

**1: Uh, amount per serving, two-hundred fifty calories. So I’d eat, have, uh, five hundred calories.** Ok. **But I, why should I have to do math, you know what I mean? It should be right there.**

**2: How many… uh, just one cup. Yeah.**

**3: So you’ve got that… well, twelve from sixty-five, right? Which is what? Uh, two from five is… fifty-three.** Yep. **Ok.**

**4: Uh, sixty-two.**

**5: No.**

**6: Ah, just… two. Yeah, ok.**

**7: Um, six, right? No, wait, yeah, six.**

**8: Ten.**

**9: Nine? No…** So what are you thinking as you’re answering that question? **Well I’m figuring, if you were advised to eat forty-five grams, and there’s a total of, uh, thirty-one, then I have the difference between forty-five and thirty-one.** Ok. **So what am I doing here… fourteen.** Ok. **Well, I mean, there aren’t that many people who are gonna stand in the grocery store and do all the math.** And actually read the label? **(?) easier kind of a thing to read. I think what it would have to do is say this bottle has this much (mumbles).**

**10: Uh, I gotta do math here. I was an English teacher. Well, I’d eat… total fat in… oh, wait, that’s a… I forgot the cup size. (reads question again) Well, I don’t know, how do you figure that percentage? That would be…** So, what would be the calculation, then? **Twelve grams of… sixty… twenty percent, I don’t…** There you go! **Yeah.** You’re the only person so far to get that right! **Oh!** Gold star for you! Really! Even from an English teacher. Yes! **Well it was a guess.** That was great!

Ok, so for that section, well, that last one was a tough one, but overall, how would you rate that section on that scale of one to five, with one being ‘that was pretty easy’ to five being ‘that was, that was difficult?’ **I would say I’d give this a three.** A three? Is that just because of the, some of the calculations you had to do in your head? **Yeah, in your head, yeah, yeah.** Ok. Any of those questions that didn’t seem to fit in with what we were asking you to do, or make sense? **No. No, I can see the strategy in all of them.** Ok. **I know I, you know, I’ve been on carb diets. You chose something that doesn’t have fiber, but, you know, you deduct the grams of fiber from the carbs, you know, to figure out the true carbohydrate.** The net carbs? **Yes. That would make this even more complicated, if you would have something…** A lot of fiber? **Yeah. Cause you’d have to calculate that into it.** Yeah, you’re right.

**So you want me to say where ‘apple’ goes?** Yeah, just tell us with each food which category you would put it in. **Well, apple would be fruits.** Ok.

**Milk would be dairy.**

**Noodles would be grains.**

**Orange juice would be in the fruits, I presume.** Ok.

**Cheese, dairy.**

**Rice, grain.**

**Tortilla, grain.**

**Chicken, protein.**

**Pork chop, protein.**

**Carrots, vegetables.**

**Butter, dairy.**

**Lemonade, I guess I don’t know, I’d put that in added sugars.** So, just a quick question for you, what made you put orange juice into the fruit category but lemonade into the added sugars category? **Because, ‘cause when you make lemonade you add sugar. We have orange trees in California, you just squeeze the orange. You don’t add sugar.** Ok, good.

**Spinach, vegetables.**

**Banana, fruit.**

**Regular salad dressing, uh, fats and oils.**

**Fruit punch, added sugars.** Ok, and what comes to mind when you see ‘fruit punch?’ **Um…** Is there a brand, or… **Well, I don’t drink punch, but when I was a kid I drank Hawaiian Punch.** Ok. **Yeah.** So you think of the red drink? The red sugary drink? **Yeah, mmhmm. And I, when I think of punch, I think of sugar.**

How was that section on that scale of one to five? **I’m very confident on this one, it’s a one, yeah.** Ok!

1: **Well, you’ve answered that, haven’t you?** So that’s one of the options; you have a, b, or c. **Ok (mumbles). I, I would say C.** Ok. They’re equal in nutrition? **Yeah.** Ok, and why would you say that? **Well, applesauce without sugar in it is just like a pureed apple, isn’t it?** Ok. **Yeah, so that’s my impression.** Ok.

2: **Well, I guess C again.** Ok. And what made you select that one? **Well I, just if they’ve added nothing to the fruit… Of course I don’t know what they, I don’t know the kind of fruit that’s in the Mott’s thing; maybe raisins are more nutritious by themselves. It’s not like apple and apple like up above.** Is that a brand that’s unfamiliar to you or is that a food that’s unfamiliar? **The Mott’s?** Mmhmm. **No. I’ve never had them, but I’ve eaten lots of raisins.**

**3: I’d say B, just by looking at it.** Ok. What about the appearance makes you say B? **Uh, well, B, I can see the fat in it. Is that what you’re asking?** Mmhmm. **Yeah. Which makes it taste a lot better. If you’ve done any barbequing you know that.**

**4: Well, what, aren’t you just answering that? Is this a statement of truth?** It’s an answer option. So you can choose either a, b, or c. **One or both? I got it.** Yeah, one or the other, or both of them are the same. **I would say the apple juice has more because it has sugar in it. Even though it’s natural sugar, right?** So is that stumbling you for, um, option C to say that both of them are equal? Is that confusing? **Yeah, when I look at, you know, when I look at that one it’s almost like it’s answered, it’s saying that they’re equal.** Because the others aren’t statements? **Yeah.** Ok. **Yeah, that’s kind of tripped me up a couple of times, just by, it’s just the way my mind works.**

**5: I’m getting this now. I would say frozen green beans.** Would it help if the answer said, ‘canned green beans are lowest in sodium content,’ ‘frozen green beans are lowest in sodium content,’ or ‘c, canned green beans and frozen green beans are equal in sodium content?’ Is it because it’s, those are just the item and this is a statement? Do you see what I’m asking? **Mmhmm.** Should it be, ‘canned green beans and frozen green beans are equal?’ Is there a better way we could state those answers? **Um, I don’t know, maybe ‘C, both have the same,’ maybe just a more direct statement, rather than saying ‘equal in sodium content.’ (reads question again) ‘Canned green beans; frozen green beans; both have the same.’** So something a little bit more— **Little more succinct.** Ok.

**6: I would say C.** Ok, and why would you select that one? **Well, ‘cause I don’t think freezing it changes the nutrition necessarily.** Ok.

7: **(mumbles) say kale. I know that green is better.** Are you familiar with kale? **Yes.** Ok.

8: **Well, obviously the Nutrition Fact Panel, ‘cause that’s where the data is.** Ok. Do you look at the Nutrition Facts Panel much as you’re, are you the primary shopper at your house? **Mmhmm. Uh, I look mainly for calories and carbohydrates.** Ok. **Like, I’m looking at turkeys now and I’m checking how much sodium is in it.** Ok, good. **But I don’t examine the entire label for everything. If it’s what I’m looking for, (mumbles)…** You’re mostly just looking for sodium right now?

**9: Um, I would say A.** Ok. Why would you say that? **Uh, I think because, uh, those, those juices, I was about children shouldn’t be drinking so much of that juice because there’s, you know, hidden sugars in it. And a lot of different kinds of fruit have more sugar than others. And they just engineer berry here. You know what you’re getting with the blueberries.** Are you familiar with that brand that we have there, the berry juice, the Naked brand, is that something that’s familiar? No? Ok.

10: **Well, in this case I guess I would say the, the package, because that, the label is just full of all kinds of words I don’t even understand. I guess in there I see, finally, there’s ‘whole grain wheat flour’ mentioned. With the soy and sugar and all of that.** Ok, ok, but you would look more at the… **So the label is obviously succinct, it says ‘five grams of whole grain.’** Ok. So the package label more summarizes what you’re… **Yeah. I think there’s just a lot more to wade through on the bottom.** Ok.

So as far as that section, on that scale of one to five—one was easy, five was difficult—how would you rate that section? **I’d rate it about a three.** Three? And why did you give it a three? **Well, because of kind of all the, the C question sometimes doesn’t seem to parallel (?)…** Yeah, let me show you, I was able to pull—I can’t pull it up on the iPad for some reason, but I can get the electronic version on my laptop—So most of the people will be taking it online. **Oh, ok.** So I’m curious, this is how it’s normally presented. Does that make it more apparent that we’re asking you to choose between one of those three options? **Yeah, what about a third picture that somehow puts the two in the same frame? So each would have its picture. Maybe that’s kind of, too, it throws your eye off. It does on this in particular. ‘Cause you have this, this, or words.** Do you, on, (?) I see. Yeah. So, in the hard copy form, it would be good under C to have both of the pictures? **Yeah.** Here, do you think that is needed? **Yeah, I, I really do, I mean it would fit my way of looking at it, because it would, each would have a statement and a picture.** Ok. Do you think that third option is easy to miss if, if people…? **Yes.** Ok, if there’s not that picture. So people might just look at—**Yeah, cause the first time I did it it was confusing, cause, ‘wait a minute, is this the answer?’** Ok. **I mean, I wasn’t in sync, I guess till I find out into it (?).** Ok. Just to make it more uniform. **Yeah. Yeah, I think so.** Ok. Ok, that’s good feedback. Um, any of the questions or any of the foods not make sense or doesn’t seem familiar, um, any, any issues with that or…? No? **No, I didn’t, I didn’t recognize a couple of the brands, but—**The brand name? **I, I don’t shop for those items, so…** Ok. **Like a fruit juice.** Ok, yeah.

**(stops after “energy-dense”) ‘Energy-dense’ makes, seems like a positive thing to me. But you’re equating that with, I figured out with sugar, but, what’s wrong with energy?** So these are, this is an unfamiliar term to you, ‘energy-dense’ and ‘nutrient-dense?’ **Yeah. If someone said, ‘those are energy-dense foods,’ I’d probably say those are good for me. ‘Cause they provide energy.** Ok. **But you’re, you’re relegating them here in this sentence to sugar, you know, kind of that.** But you figured that out from the description. **Well, when I thought about it, yeah. ‘Cause it seemed, didn’t seem (?).** Surprised you? **Yeah.** Ok. **(keeps reading; stops after energy-dense paragraph) Well, that explains it.** Ok, so… **So now it explains what that word means.** Ok, so that helps. When you first got to that ‘energy-dense,’ it tripped you up a little bit. **Yeah.** It’s unfamiliar. Is ‘nutrient-dense,’ is that also an unfamiliar term for you? Have you heard of that? **Well, I mean I’ve never used the word ‘dense’ when I thought about nutrients. I know what that means when I read it. I mean, a healthy diet is high in nutrients, in nutritious foods. ‘Dense’ means…** High in. So is… **Packed in there.** Is there a different way that you would phrase that second paragraph so that, maybe, it was more clear? Or, um, is it, does it just kind of take reading along to understand it? **Well, I mean, a simpler way would be, “A healthy diet has lots of nutrients.”** Ok. **You know, the word ‘dense,’ I don’t know if you need to…** Then what about that, then let’s take that, the second sentence. **“A healthy diet also is low…” Um, “A healthy diet is also low…” But, see, ‘energy,’ it just seems in my mind as a positive thing. And the way it’s used here, it’s a negative, it’s construed as a negative, or too much… “A healthy diet is also low in refined grains and added sugars.”** Ok. **I don’t know.** Ok. **Unless you’re gonna use ‘energy’ all the way down through… “While these foods can provide energy, too much energy can lead to weight gain.” I don’t know if you need to put ‘energy-dense’ in there at all.** Ok. Would, maybe, ‘calories’ make more sense? Or, um, is there another term that maybe—**“A healthy diet is also low in refined grains and added sugars. While these foods provide energy, too much energy…” I think it’s just as clear to me if it didn’t have ‘energy-dense’ at all.** Ok. **(continues reading; stops after nutrient-dense paragraph) Again, I don’t know if you need the word ‘dense’ at all.** Ok. **(mumbles) make sense of it. “In order to follow a healthy diet, eat foods with more nutrients. Fruits and vegetables are examples of high-nutrient foods.” Instead of the word, the word ‘dense’ throws me off.** Ok. **(continues reading; stops in whole grains) Alright. “Whole grains are full of nutrients and may improve weight control and decrease…” Or “are high in nutrients.”** Ok. **That’s simpler to me. “Whole grains are high in nutrients and may improve weight control and decrease disease risk.” (continues reading; stops after proteins) “…are also high in nutrients, when prepared with few added…”** So, since you’re an English teacher, I’m just, I just want to take a minute and explain. Part of what this section is intending to do is to see if people can understand unfamiliar terminology from the description. **Mmhmm.** So, it’s kind of an intention to have ‘nutrient-dense’ and ‘energy-dense.’ Um, but what I want to know is, can people who can read well, can they figure out, could they define that later, after reading this? **Oh, I’m sure, yeah, yeah.** Ok. ‘Cause I just, yeah, I felt like you might be someone who would understand that… **Oh, I understand it, yeah.** Ok, so it makes it clear. **I’m just, I just… style, it sounds a bit jargon-ish.** Yes. **And… Because most people don’t go to the store and think of density.** Right. **So that has a lot of, you know, ‘that kale is more nutritious.’ Not, ‘it’s more nutrient-dense.’** Right. **(continues reading; stops after the energy-dense explanatory paragraph) I think most of us know that. (continues reading; stops after sodium)** Is ‘shelf-stable,’ is that something that’s familiar, a familiar term for you? **No.** No? Ok. Would you use something different in place of that? **Uh, well you, by that you mean, um…** What do you think of when you think of ‘shelf-stable’ foods? **Well, things that are on your counter and you use them when you want to, when you need to use them. Yeah.** Is there anything, is that pretty self-explanatory, or would you use… **Well, I mean, you have to think about it, you know. I had to think about that phrase; I don’t think salt is shelf-stable.** Not a familiar phrase? **Yeah.** How would, is there another word that you would use that you think more people would understand? ‘Cause that’s not one that we’re intending to, for people to have to figure out. **Well, of course, you don’t want to add a lot of words either and make it too long.** Right. In the previous version we used ‘processed.’ But then it was—**Used what?** ‘Processed foods.’ **Oh. That’s pretty minimal, though, isn’t it? (re-reads sentence)** So we’re talking about canned soup, boxed mixes, um, convenience foods. **Hmm… ‘packaged?’** ‘Packaged food?’ Ok. That’s a good suggestion. Yeah, I like that one. **(mumbles) Yeah, I have to think about that one to figure out what it means. I mean, I can visualize the salt shaker sitting there, but I don’t visualize, you know, a box of something.** Ok. **(mumbles).** Yes. **(finishes reading)** So, overall with that section, once you got past the, you know, the ‘dense’ part, um, do you think somebody, you know, on a scale of one to five, would be able to learn something from that? **Yeah! I’d say a four. Just with those little (?), you know, I’d say a four, yeah.** Ok. **A lot of it, I mean, most people, I think, watch Dr. Oz once a year. And I’ve seen some of his stuff, you know, we know we’re not supposed to drink a lot of coke with the sugar in it.** Right. So with some of the terminologies… just some of the terminology might be new for people, but they could learn from, based on what has been described. **Yeah, I think so.** Ok.

1: **Whole grains, C.**

**2: Ice cream.**

3: **An orange.**

**4: Cholesterol.** Ok. **I was thinking about carbohydrate or cholesterol.**

5: **Nutrient-dense.**

**6: Animal-based.**

**7: C.** Ok. And why did you pick C? **Um, the ‘skinless’ jumps right out at me, and the steamed rice and the vegetables.** Ok, good. Ok. **And the low-fat milk, which they all have that.**

**8: Blood pressure.**

**9: Chocolate pudding.**

**10: Lemonade.** Ok.

11: **Canned tomato soup.**

Great. Good. So on that section, one, easy, to five, difficult, what would you rate it? **A one.** One? Ok. **Oh, wait a minute, have I been doing the wrong…** No, you’re fine. **One is not difficult. Ok.** You’re fine. **Good.** Any questions that didn’t seem to fit in there or didn’t make sense or, or…? Ok, you flew through it very quickly! **The canned soup one I had to, well, because I, cause it’s always amazing to look on those cans. I am sensitive to sodium, and I mean, it’s amazing to see how much sodium is in that processed stuff!**

***Interview 8***

Macronutrients:

1: **Those are a source of fat.** Ok.

**2: I have never heard of that one.** Of folate? Ok, that’s an unfamiliar term for you? **Yeah.** Ok. **A, sugar.**

**3: That would be, uh, B, protein.** Ok.

4: **I don’t really register things really well.** That’s ok. Take your time. … **Uh, C, pork chop, egg, and cheese would provide more protein.** Ok. Was that one fairly easy for you to arrive to, or were there…? **Yeah, yeah. Of course, I mean, I’m, … I’m probably reasonably knowledgeable about this stuff because at one time I was a body builder and, back in my youth, and wanted to be a sports doctor.** Oh, ok! Cool! **So, I have some knowledge.**

**5: Uh, it would be D, mayonnaise, margarine, and almonds.** … For that question, was that one fairly easy for you to arrive at that answer, or were there any others that were… a selection…? **Uh, well, the… no, I mean the corn, the corn tortillas would be kind of fatty.** Ok. **But other than that, it would be pretty obviously D.** Ok, with the selections that are provided there? **Yeah.** Ok.

**6: Carbohydrates, that would be A.** Ok.

7: **Probably A, salmon.** Ok, is that, um, ‘healthy fat,’ is that a familiar term for you? **Yeah, I can’t remember the, what it’s called. I think it starts with an H, but I’m not sure.** Ok. And so when you saw salmon, why did you choose that over the other ones? **Uh, because sausage is really fatty and, let’s see, cheese is fatty, and bread is a carbohydrate.** Ok. A pretty obvious choice for you? Ok, good.

8: **Uh, probably B would be the most likely answer.** Ok. **Uh, the other one could be bread.** Ok, could be bread? What made you choose the regular salad dressing over bread? **Ah, ‘cause it’s, it’s common knowledge that that’s (?).** Ok. **Got a lot of calories, bad calories in it. I love it anyways, but…** Tastes better, doesn’t it? **Yeah.** Ok.

9: **Uh, D.** Ok. What made you select that, that answer? **Uh, again, I can’t remember the, the whatchacallit fat.** Ok. **But, uh, it’s like fish, it’s kinda like the salmon, the stuff that’s in fish tablets, I think, that same type of fat.** Ok. **I think. … But I can’t remember that, what was that fat called?** Are you thinking of the omegas? **Well it could, I thought it was, it seemed to have started with an H. That’s what I want to say, is that it starts with an H.** Usually we just call them ‘polyunsaturated fats.’ **Yeah, that’s, that’s it. See that’s the problem with my, my memory.** I think they’re very complicated words with the fats. Yeah.

Ok, so for that section overall—‘cause you went through that actually very quickly—um, how, if I gave you a scale of one to five, so one, ‘that’s pretty easy,’ to five, ‘that was pretty difficult,’ how would you rate that section? **One to get easy, you’re saying?** One was easy, five was difficult. **Well, for me it was one.** One. And why would you rate it as a one? **Well, probably because I do have some knowledge of this stuff.** Ok. **Uh, some of the people that I know, they’re, uh, … not quite, they’re not as knowledgeable or they might, not as quick to understand this stuff.** Right. **So they might have a problem with some of it.** Ok. **And mostly because they don’t know the differences between fats and sugars, carbohydrates and stuff like that.** Right, yeah. Ok. Um, other than folate—I know you mentioned folate was unfamiliar to you—was there any other unfamiliar—**What is it?** It’s a B-vitamin. **A B-vitamin?** Mmhmm. So it’s one of the—**Like a B-12?** It is very similar to B-12, yes. **And, what? I’m sorry.** No, that’s ok, were there any other unfamiliar terms or foods in that section? **Uh, visually, the ‘which breakfast is highest in carbohydrate’ question, visually, that is a nightmare for me to read.** Ok, why is that? Too much information, or…? **Uh, it, well, for someone with, uh, with ADHD and/or dyslexia, it is just difficult to read.** Ok. Is there a way we could format it differently using the same kind of content? Or…? **I, me personally, I would get rid of the ‘eight ounces’ and just say ‘orange juice.’** Ok. So the numbers combined with words…? **Yeah, it’s kind of confusing, kind of. Like I say, visually, it messes with you.** Ok. **It makes it, it just makes it more difficult to read.** What about reading on a tablet? Does it matter? **Well, I mean, no, I don’t think it would make any difference whether it was on a tablet or on paper. It’s just…** The numbers combined with the text? **Yeah. Well, you’ve got ‘A,’ you’ve got the letter A there. And then you got 8 ounces immediately after it, which I know is common in, you know, school questions and stuff like that, multiple choice questions, but it, visually for somebody with learning disabilities, it sucks.** Ok. That’s good feedback. Let us know, you know, as we go through this, if there’s any others you can see as potential problems for people that have learning disabilities. ‘Cause it’s more often than not anymore that somebody’s got some type of learning disabilities. …

Portion sizes

Ok, stop. So this is where I actually really want your feedback. So, you have a learning disability; what, tell me about that paragraph, those instructions there. Tell me what you think it’s asking you to do. **Well it, it sounds like it’s just asking me to decide what is the healthiest portion. Uh, it’s a little wordy.** Ok. Is there anything that you think we could take out, or is it, could it be worded differently? **Well, I’m… that kind of stumbles… besides algebra was, I, I could not do, just failed miserably in, in English, in English language, so, or what it takes to write it, so I couldn’t tell you how to write it.** Ok, that’s fine. If we, um, took out the first two sentences and just went to ‘for each picture?’ Do you think that gives you adequate amount of instructions to do what you think need to be done? **(reads) Well, yeah, I mean, as far as, uh, answering the questions, that would be adequate. If you’re still wanting someone to learn, then we kind of need to leave that other stuff in. But it just seems (?) work, to me.** Ok. And then, um, what do you think of ‘portion?’ What does that mean to you? **Size.** Size, ok.

1: **Well, it, that is the correct portion.** Ok. **Supposedly. I, I don’t think so…** Do you think it should be more? **Well, I usually drink between sixteen and, or more, twenty ounces of milk every morning.** Ok. When you answered that question, did you use, did you rely more on the text, did you look at the picture, or was it about the same? **It’s just more, well, no, the picture, I don’t know, it might confuse somebody that there’s cereal and an apple in it, you know.** Too much going on? **Well, I mean, ‘cause you’re asking about the milk. I’m, I’m thinking of, not me, but some of the folks I know.** Ok. **I didn’t even look at the picture.** You didn’t even look at the picture to answer the question? Ok.

2: **Well, for me it’s not even close to being the right… I’d guess B. It’s probably C.** About right for one portion? Ok. **Yeah, it’s probably what you should eat.** Ok. Not necessarily what you would eat. **I would starve to death, but it, it, C would probably be about right.** Ok. **I suppose if that includes the potatoes, they look like potatoes, and, uh, I don’t know what you’re supposed to do with that green thing. Throw it in the trash, that’s what I’d do. …** Again, did you rely more on the text, did you use the text to answer the question? **No, I had to look at the picture on that one because I have no idea what five ounces of chicken even is or…** So it’s easier to visualize a cup versus weight? **Well, I mean, as far as, like the milk you mean? Well, that’s just because everything that, instruction on anything you drink says, you know, eight ounces a portion.** Ok. **So that’s kind of drilled into your head, for, you know, the last forty-five years or so.** Ok. **But as far as the chicken, yeah, I have no idea what the right portion is. Uh, a pound of hamburger, to me, is one portion.** Ok, gotcha, ok. **So…**

**3: I can’t believe that’s one cup of rice, but it would probably be C.** Ok. **It looks like more rice than just a cup.** For that one, did you automatically assume the rice, that it was cooked? **Uh, yeah. I think so, yeah.** Ok. **You are right, it would be a lot more rice than a cup if it wasn’t cooked.** Ok. **Because it gets puffy. One cup of uncooked rice would be much closer to [my] portion. But I guess C would be the answer.** Ok.

4: **I eat a bag at a time. Uh, so that, B, that’s less than one portion.** Ok. **It may be about right but it’s…** Doesn’t seem right? **It doesn’t seem like enough.** Ok.

5: **Two cups of pasta noodles… cooked… (?) two cups.** Ok. … **That would be reasonable, if you went to a restaurant. C.** Ok. Do you think like for the rice and pasta that we should put in there that it’s cooked cups, or do you think that people automatically just assume cooked? **It, it might be, uh, it might be better just to say ‘this is one cup of cooked rice’ or ‘cooked noodles’ so they know. ‘Cause again, noodles would, I don’t know, there’d probably be a lot more noodles if you, if they weren’t cooked.** Ok. **I don’t know how you get that many noodles into one cup though. A cup’s not very big. I mean… experience.** Ok. **How the heck did you get that many noodles in…**

**6: Half a cup of black beans, that would be (mumbles) it would probably be plenty for me. That’s one of those foods you gotta more hide.** It’s gotta be mixed in with some other good stuff? **Yeah.** So are you answering it based on what you typically eat or what you think a person should eat? **Yeah, I think, I don’t know any other way to answer it.** Ok. So you’re answering it how you would judge it, based on yourself? **Well, I mean, knowing what, what you guys want people to know is the right portion and based on what I eat, I, you know, it depends, I mean it, if I just, if you were to before the test tell everybody, ‘answer to what you feel is the right portion, not what we expect you to answer.’** Ok. **See what I’m saying?** Yes. So what you would eat versus what is standard. **Right. Well, I mean, cause you’re trying to teach people proper nutrition, right?** Right. **So if you asked me to answer, answer, you know, what you would eat.** Portion yourself. Ok. **Yeah, and then you, cause then you can come back and if you’re gonna discuss the test with them—I don’t know if you are or not, but—if you were to discuss the answers with them you could be able to tell them, well the answer, this is what you, and that’s, this would be the proper portion, so what you’re eating may be, you know, need to cut it back a little bit.** Ok. **Or something like that. I don’t know, I don’t know. …** Ok, so, do me a favor and just go back to those instructions real quick and read through those again. **(reads)** Ok, so what do you think, especially with those last two sentences, what are, what are…? **Well, it, uh, that makes more sense now. It’s… doesn’t seem as wordy.** Ok, right, but the… Cause you’ve already done a couple of them? **Cause you think of the, ‘cause now you know what you’re… think.** Ok. Ok. So if it’s, so ‘for each food pictured, choose what you think is the right portion size,’ should we add something in there so that it’s um, not necessarily… **Well, yeah, maybe just what you said. ‘For each picture, choose what you believe is the correct portion size.’** For you? Or… should we add that? **Well, yeah. It would, what, yeah.** Ok. **‘What you would eat,’ or… I don’t know how to word it.** That’s ok. Yeah, the wording on this one is tricky. **But to get it so that… ‘cause if you’re wanting to know what they eat or just what they believe you want to know…** Ok. **‘Cause they’re not really answering it honestly if they’re just answering for what you want to know. ‘Cause especially with, again, I’m not trying to be mean, and it’s not you, this is to the people at the center. Uh, quite a few of those folks are way, way, way overweight. Really, really heavy. So they’re gonna look at those and they’re gonna be like, ‘I, I…’** It’s too small? **Yeah.** Ok. That’s not enough? Ok. **So, uh, yeah, probably need to ask what you would eat. How much, if you believe it’s too little for you, or, or not as much as you would eat. I don’t know if you’re asking those questions. Is this just a test you take and you don’t see the person again?** Um, so, what, in the future we would like to use it so that we can see, um, where do people need more information? Where do they need more help? **Right, I mean, are you gonna talk to them after the test, are you gonna…?** In this study, no. But the idea for using the instrument in the future, yes. **Well then if you’re not gonna talk to them you definitely need to ask them what they’re eating, how much they’re eating.** We are doing that too. Yeah. So this is more asking what their knowledge is. **What their knowledge is.** Of what is a correct portion. **Well then, then you need to word it in that way.** Ok. So right now the way it’s worded you think it’s asking what you would eat. **Well, no, to me it’s not really asking either one.** Ok. It’s unclear. It’s very unclear then. **Yeah.** The instructions are unclear. It’s unclear whether we’re asking you to tell us how much you would eat compared to what’s on the plate versus how much you should eat. **It does say ‘choose what you think is the right portion size,’ but, but…** According to what? According to you? According to standards? **Right, right.** Ok. So maybe a little bit more specific as to knowledge, you know, or knowledge or personal. **Yeah, what you know as, what you know personally as the correct… If that’s what you’re really wanting to find out.** Ok. Ok, good. Good. Ok, that’s good feedback, thank you for that.

**7: Well, again, I hate carrots, uh, but, yeah, I’d say that, if I was answering it as to what is the correct portion, uh, that’s probably C.** Ok. **I think most of them are shown as about the right portion, to be honest with you. Not, there’s no, uh, trickery in it. You know, like you get in school with questions, you know, multiple choice questions, there’s usually a trick.** Yes. We’re not trying to trick you.

8: **Uh, yeah, again, the correct answer is most likely C. And I, that just bugs me about, it’s not really, cause most people are not, are gonna say ‘gosh, you know I’m gonna starve to death on that.’ That’s what they’re gonna be thinking when they see that.** Ok, when they see these pictures. Ok. **I would have trouble eating this diet. I mean, and I don’t eat much. But I would have trouble eating this diet if I ate regularly like I should, or could afford to eat regularly like I should.**

**9: I know you probably want three, but…** Why would you say that we would want three? **Well, because it’s the smallest. I mean, it’s just, that’s what most diets recommend is low, low calories. And, uh, although you’d be losing quite a bit of protein by eating that one…** So you think people would be most likely to guess three even if they didn’t know the answer? **Correct.** Ok. **You know, unless, like, they’re answering it for what they think, what they like or what they want. For me, eight ounces would be a much better option because you get a lot more protein. I will eat protein a lot more than I do other stuff. If I could afford a protein diet, that’s what I would…** Would generally consume? **Yeah. I think it’s much healthier for you.**

**10: Well, I think we’ve determined that one cup is, is probably the right portion by the earlier answer.** Ok, so relying on what was asked earlier. **Mmhmm. Um, a quarter of a cup would not, that’d barely keep a small child alive for very long, so…** Ok. So in this question, you think it’s pretty obvious that the smallest one isn’t the right answer. **Right. I mean, you couldn’t survive on that. I, I know that people in Vietnam and other third-world countries like that eat a lot more rice than that little (?) right there. But that’s just my knowledge of that.**

**11: That is a little confusing. I mean I understand what it’s asking, but it’s a little bit confusing.** Is it too wordy or…? **Um…** The way it’s asking the question? **Yeah… I’m trying to think. (whispers) Maybe if you just, ‘which of the following…’ (mumbles)** That’s ok. As it is now, it’s a bit… **Well, see, yeah, it’s a little, it’s a tad confusing when you first read it.** Ok. **And, you know…** Has to take a few more times to really be able to process what it’s asking? **Well, yeah. When I first saw it, it was like ‘huh?’** Ok. **So, (reads answers again) And a serving size is… really hard to read, oh there it is, two tablespoons. So, if, ok… So you’re just asking us to match that? Ok. So it would be B.** Ok. **I think somebody might look at that and say, ‘why did you ask that?’** Too obvious? Or… **Yeah.** Do you look at, do you read food labels? **Oh yeah.** Yeah? Ok. **Not all the time, I mean, but I do read them. Depends on what it is.** Ok. So you said some people would look at that question, ‘why’d you ask me that,’ because it’s… **Well, it just, I don’t know.** Does it seem to fit in there, or…? **No, it… because the answer is right there, I guess.** Ok. It’s just a matter of looking… Do you think it’s a trick, or…? **No, it just, it… I don’t know. You gotta remember, I see things so screwed up.** No, this is fine! **The way I process, it’s just kinda, I don’t know.** So is it one of those—**It’s too obvious.** It’s just too obvious. **Yeah.** Ok. So even somebody who might not be familiar with reading food labels you think could still even match up? **Yeah. It’s just a matter of being able to see it, because it’s really hard to read.** Ok.

So, for that section overall, um, I think we need to, I think we need to work on the instructions, but if we, if we were to revise those instructions a little bit and ask instead of, you know, ‘choose what you think is the correct portion,’ you know, according to standard. **Specify what is the correct portion, as opposed to what you would eat.** Ok. **Now, you might even say that as, ‘what is the correct portion as opposed to what you would normally eat?’** Ok, that’s good. That’s really good for us to know. So, if we put that in the instructions, um, it made it, you know, easier for that section to answer, based on just overall, based on that section, how would you rate the difficulty of that section with one being—**Well, it’s not hard. I would actually mix these up, not put, make every one of them C for example.** Ok… ok, so… **Cause it, it kinda comes across, makes you, ‘this is a waste of time.’ You know, I mean you kinda, if, if they’re all the same, cause you’re trying to make them think. You might even word those differently or something like that, might actually put in some, something to trip them a little bit. Not, you know, not bad or hard or anything like that, but to make them think about it. Instead of being able to guess, say ‘ok, C.’** C all the way through? **Yeah. I mean, I always used to love it when teachers put the answer in D every time…** Ok.

Nutrition facts label

1: **Let’s see… two servings… so that would be five hundred calories, so you’ve got (reads answer selections). So, it would be B.** Ok. **That’s what I mean by that last section is don’t all obviously C. It’s probably good for, to make them think.**

**2: Fewer than five hundred milligrams of sodium… (mumbles) cups… serving… so it would be one cup. So that’d be A, one cup.** Ok. **That one’s fine, it’s, um, I mean, it’s confusing but that’s, it’s not, not in a way that it’s bad, it’s just…** You have to read it again? Back and forth? **Yeah, you have to think, you have to really think about it. Which is not bad. That’s not a bad thing.**

**3: (mumbling) total fat… is twelve grams…one cup…ok. So, it’s sixty-five minus twelve… fifty three.** Ok. **C.**

**4: (mumbling) two… total carbohydrates… That’d be sixty-two, C.** Ok.

5: **Yeah, I think macaroni’s got fiber… Pretty sure that’s a fiber food, so yes.** Ok, so you were doing that based on your knowledge background? **Well, I’m trying to remember, yeah, I just can’t remember if macaroni is a fiber… There’s what I couldn’t think of is what it’s made out of. It’s made usually from wheat, so that should be a fiber.** Ok. **I don’t know if everybody would know that it’s made from wheat though. So, most people actually probably wouldn’t know that it’s a fiber. And, and if you’re trying to… Yeah, most people probably wouldn’t, I mean, hearing some of the answers that people give to questions like this, you know, just on the street questions, most people don’t have a clue. So they wouldn’t know that it’s, what it’s made from. But it’s not a, not a bad question or anything like that, I’m just saying they wouldn’t know that it’s made from wheat or some kind of grain.** Ok.

6: **So, saturated… ok, saturated fat per meal, seven grams per meal… you can eat B, two cups.** Ok. **Uh, when you first see that question, it, you think you already did that.** Ok. **But there’s nothing wrong with the question, it just, you’re like…** I saw that before, or… **Yeah, didn’t I already do that question?**

**7: (mumbling) So that’d be six grams, D.** Ok.

8: **(mumbling) five grams… so that’d be B, ten.**

**9: Forty-five grams… is thirty-one… forty-five grams, oh, duh. So it’d be fourteen, so C, fourteen.**

**10: (mumbling) Five?** Ok. **No, there is no five percent. It would be… one fifth, so… (mumbling) one fifth of sixty grams… percent… twenty percent, C.** Good for you! Good! **Or, D, rather.** Now, we’ll tell you that there’s only been two people that have gotten that one correct. So, you and the gentleman just before you. So, see, you’re smarter than you think. Yeah! It was good for us to hear how you processed through that one. No, that’s a tough question, it involves more math. **Yeah, it is.** Or higher level math. **Well, yeah, more math than I’ve probably used in at least ten years, anyway.**

Ok, so, let me just ask you one more follow-up question for that section. On that scale of one to five, from pretty easy to five being difficult… **That’s five.** That was a five? And was that mostly because of the math? **The math, yeah.** Ok. **Cause I don’t think that most of the folks that I know—and I keep using the Elizabeth Layton Center as an example, cause I think those are probably an example of the people you’re targeting anyway—they would have a harder time with the math.** Ok. **Much harder; they might quit.** Ok. Well, you did a great job.

***Interview 9 and 10*** [Interviewed together]

(after ‘achieve a healthy weight) Ok, let’s stop there. Um, do you have grandchildren? **Yes.** Ok, so how would you explain, in your own words, those few sentences that you just read to your grandchildren? … **Oh, I couldn’t. …** So how about, maybe, one of your friends, then? How would you explain it to a friend? **Eating a healthy diet is a key factor in your life because staying healthy may help you maintain a quality of life. A healthy diet could prevent chronic disease and improve the quality of life.** Ok, now tell me about the phrase ‘healthy diet.’ What comes to mind when you hear ‘healthy diet?’ **(after nutrient- and energy-dense definitions) Define ‘dense.’** Um, is that a familiar term to you, ‘dense?’ **Well, that’s less. Um, dense fog or the density of water, so is it less than normal?** ‘Dense,’ you said ‘dense fog,’ you’re thinking of fog? What does it mean for fog to be dense? **Can’t see.** You can’t see, so what’s happening? **Fog has density and … you can’t see as far.** So the air is full, you can’t see it. That’s what a dense fog means. So, let’s kind of read ahead and see if what’s written here helps you to understand what those terms mean or not. So, can you read the next couple of sentences? **(stops after next paragraph)** Ok, so, after you’ve read that, those couple of sentences, how would you explain the term ‘nutrient-dense?’ Or give some examples of foods that are nutrient-dense? **Ok, um, (?) I guess are nutrient-dense because they provide many vitamins, minerals, and other… (?)** That’s good! … … … Um, so what word would you use instead of ‘nutrient-dense?’ **Elevated.** Elevated? **Yeah. Because ‘dense’ means low, to me it does.** Ok. **‘Elevated’ is high.** Ok. Ok. Um, well, that’s helpful. Let’s go on to the next few sentences. **(reads starting at ‘eat less’ section; stops after first paragraph)** Ok, so, when you read those couple of sentences, what comes to mind about the word ‘energy-dense?’ Does that help to understand ‘energy-dense,’ or are you still confused? **Oh, I understand what it means, but it seems to me that the word is used in the wrong context.** Ok. **When I think about… to me it would be ‘nutrient-elevated. (?)** Ok, it’s confusing to you because the word ‘dense’ sounds like saying the opposite of what you think we mean? **Yes. … … …** Ok, so after we’ve read through that section, I want to think about a scale of one to five, one being ‘this was pretty easy to read and understand’ and five being ‘this was really challenging to understand.’ Where would you, I’m going to ask each of you to rate it. So, do you want to give us your rating first? One is easy, five is hard. **I would say about a three.** Three, so in the middle? **Yes.** Ok. Not too hard, but not too easy? **Right.** Ok.

(after instructions) So I’m gonna stop you there, you kind of stumbled a little bit; is it worded funny to you? **I had already a, my mind processes something that I’ve already heard, and in here, uh, ‘nutritional information you already know’ and I was assuming they already have heard.** … Ok, now tell me about the phrase ‘healthy diet.’ What comes to mind when you hear ‘healthy diet?’  **Eating the proper foods.** Ok, it doesn’t seem like a negative term? Ok. **(Continues reading from “a healthy diet is high in nutrient-dense foods)** Ok, so are there any unfamiliar terms, ok, what are the…?  **‘Nutrition-dense foods,’ you know, that’s just a blank in my mind.** Ok. **Because the word ‘dense,’ you think little or very low. I don’t know, it changes the thought.** Ok. It changes what you think of when you think of nutrition? **Mmhmm.** Ok. **Because you used the word ‘dense,’ for me.** Ok, what about ‘energy-dense?’ **Oh, ok, that was the other one. … (after lean proteins paragraph)** Ok, do you want to tell us a little bit about what ‘nutrient-dense’ means? How you would explain it to a friend? **That ‘dense’ is throwing me off.** The word ‘dense’ is hard? **Mmhmm.** Ok. **I mean you don’t read it that way, and maybe that’s the problem. Whenever you read about nutrients, it’s generally by itself, and then this surrounding, it’s added, and so it’s throwing you off, well, throwing me off, I should say.** Ok. So, since that’s a new term, from what you’ve just read, how would you try to describe it to somebody else? **Um, I could describe the whole grain for those who didn’t know whole grains. I’d be able to tell them that whole grains are, uh, well, examples of whole grains would be wheat, rice, oat, and barley. And, you know, I’d look at this and say whole-grain foods are nutrient dense, but I don’t think me sharing that with someone else, I don’t think it’d make any sense.** That’s not the word you would use to describe it? **No.** Ok. Um, so what word would you use instead of ‘nutrient-dense?’ … **That word ‘dense,’ it seems, it takes away from what you’re really trying to say.** … **(reads from ‘refined grains,’)** Is that an unfamiliar term, ‘shelf-stable?’ **Mmhmm. (continues reading through sodium section)** Ok, so you paused at ‘shelf-stable.’ What kind, what kind of food comes to mind when we say the shelf-stable foods? **I guess foods that won’t, uh, yeah, foods that won’t spoil, that can be stored on the shelf.** Ok. Is there a different way that you would say that? **Well I guess, I guess my question would be why did you have to put ‘stable’ in it? Was there some kind of benefit, whoever is writing this, to say that word. To me, you want me to visualize the shelf and then think about what would go on the shelf. But I wouldn’t tell, I mean, when I talk to somebody I wouldn’t think to say ‘stable foods.’** Ok. Just ‘shelf foods?’ **Mmhmm.** Ok. **Makes me think of life of the foods when you say ‘shelf,’ then when you throw ‘stable’ in, I’m thinking (?).** Ok. **It’s a different way of thinking. That’s what it is.** So, um, you described foods that wouldn’t spoil. Can you name some products that you’re visualizing? **I think about canned foods, um, which I would put on the shelf. Um, or something like salt and pepper, um, spices should stay on the shelf. That’s what I think of. Um, oil… I could think of a lot of different things.** Ok, great, great. Um, do you want to just finish up the last couple of sentences for us then? **Ok. (finishes reading)** Ok, so now that we’ve gone through that, um, this last section was on energy-dense foods. Can you describe what you’re thinking of when we use the term ‘energy-dense foods?’ **I think I can understand energy-dense foods better just by the breakdown here. When we talked about, I thought about myself, um, when we talked about saturated fats and cholesterol and the examples of blood, uh, cholesterol in the blood, refined grains. Uh, I can understand the ‘dense’ because you talk about white bread and saltine crackers. The energy-dense when you talk about ‘such as chips, soda, fruit juice, and desserts.’ I mean, I can understand that ‘dense’ but I can’t understand the other one.** Ok. **It’s deeper here than in the other part.** So the examples in this section are really helpful for you to understand what ‘energy-dense’ is, but there aren’t enough examples in the ‘nutrient-dense?’ **Yes, right, right.** Ok. **Cause these are all things I shouldn’t have.** Ok, so after we’ve read through that section, I want to think about a scale of one to five, one being ‘this was pretty easy to read and understand’ and five being ‘this was really challenging to understand.’ Where would you, I’m going to ask each of you to rate it. … **Same [three] for me.** Same for you? Ok.

1: **C, I would say.** You would say C, whole grains? **Yes.** Ok, and how did you come to that answer? **Because the rest is fattening.** Ok. **(?), red meat, all that’s fatty food.** Is the term ‘whole grains’ and ‘refined grains,’ are those terms familiar to you? **Yes.** Ok, so when you answered this question, did you answer it from knowledge you had before you read the text? **Yeah.** Ok.

1: **Yes, the same thing [answer C]. I kinda knew some from over the years, and that just seemed like the natural one because the rest of them seem like they would make you gain weight.**

**2: I’d say A.** Ok, and why would you choose A? **Because I seem like I must eat a lot of energy-dense food, from what I read earlier. I didn’t realize that’s what it was, but that’s why I chose ice cream.** Ok, so it’s because of what you read before?

3: **Nutrient-dense would be A, soda. No, because I keep thinking that ‘dense’ is low.** Ok. **Probably French fries, because the rest are high.** So because you think that ‘nutrient-dense’ means that it’s low in nutrition, you would choose French fries. **Yeah.** Ok. If ‘nutrient-dense’ means that it’s high in nutrition, which would you choose? **High in nutrition?** Mmhmm. **Or high in calories. That would be regular soda.** You’d choose regular soda, that would be highest in nutrition? **That’s not right. An orange.** An orange, ok.

3: **I’d pick the orange.** Ok.

4: **Um, I’m not sure. I don’t know.** Ok. Do you want to read through the question again and see if that helps? **So if it’s low in saturated fat, it wouldn’t have high cholesterol. It could be carbohydrate… I don’t know.** Ok, so that one’s kind of a puzzle? Ok. So you would just skip this one if you were asked…? **Mmhmm.** Ok. **If I had time to look at it later I could go back, but…** You’d need more time to think about it? **Mmhmm.** Ok. **I think I throw myself off when I see saturated fat and lowered saturated fat. And so when I look at the choices I have I draw a blank.** And you’re thinking if it’s already low in saturated fat, cholesterol couldn’t be the answer because it’d be low in cholesterol too? **Right. It’d (?) cholesterol.** Ok. Ok, so you’re kind of thinking maybe it’s a trick question? Or… **I just don’t think I know enough.** Ok. … … … If you go back to the text, is that what you’re saying you would do, go back to the text to find the answer? **Right. And the text wouldn’t be written the way the question is written, the text would be…** And you are allowed to back and read it, so if you want to scroll back you can. **Well, it does say cholesterol. This is the one: ‘saturated fat and cholesterol are nutrients…’ not that part. ‘These nutrients may increase the body’s level of cholesterol in the blood.’ So when you read the question… ‘saturated fat, sodium…’ I guess it’s cholesterol.** Ok. **It’s got cholesterol in there but I didn’t read it that way.**

4: **I would think calcium.** You would think calcium? **Yes.** Ok. Is there something about the way the question or the sentence…? **No, it’s understandable. I just don’t understand…** Ok. **(?)**

5: **It would have to be energy, I guess energy.** You would choose energy? **Mmhmm.** Ok. **(mumbles) broccoli.** How did you choose that over the other answers? **I didn’t consider the answers. When I saw the word ‘dense,’ I’ve only read two or three different ‘dense,’ and I didn’t see any other options.** Ok. Ok.

6: **Animal, yeah.**

**6: A, animal.** Ok, so you chose that and didn’t even have to look at the other three.

7: **I couldn’t even answer that because I wouldn’t know how to tally that up.** Ok. So what is it looking for? **Well it’s looking for nutrient-dense, but I can’t tell from… I guess if I take my time, but, uh…** Would you normally just skip it? **I would.** Ok. **Because, to me, it’s asking me to plan a meal and I don’t know enough about low-fat or nutrient-dense to understand without a lot of thinking and going back and reading and figuring it out.** Ok. And so, are there any answers that you would rule out? Just out of looking at them, they can’t be right? **Um, I would say B.** You would get rid of B? **Mmhmm.** Ok, why would you get rid of B? **Meat sauce, the garlic bread… it has low-fat milk, but I would think the meat sauce and the butter and stuff on the garlic bread.** Ok. Any of the other options you can rule out? **Um… (mumbles)… well, you can have a half a cup of rice… uh… hamburger, potato chips… I don’t know.** That one’s really stumping you, huh? **Yeah. The twenty potato chips, I don’t… a handful of potato chips or twenty potato chips is, uh…** Not sure if they’re nutrient-dense or energy-dense? **Yeah.** Ok. **But I know in nutrition, you know, they have a way of balancing these meals that you eat, but I don’t know how to do it, so this is difficult.** Ok, it’s pretty hard? Ok.

7: Anything you want to add on that question? Do you have an answer? **No, not specifically. Uh, I would say D.** D? Ok.

8: **Uh, I would say A.** You would say blood glucose? **Yes.** Ok. And why did you choose blood glucose over the other options? **Uh, because it would lower the blood glucose, glucose (mumbles) I’m not really sure.** Not sure, but that’s the one that you would guess? **Yes.**

**8: Blood pressure. My doctor’s always talking about me lowering my sodium intake because of my blood pressure.**

**9: Added sugar… I would say chocolate pudding, D.** Ok. How did you decide on that one? **Well, I didn’t think milk had added sugar. I’m thinking of what they already have, does this mean if I added some sugar, that’s what it’s saying?** Mmhmm. **I was thinking of chocolate, there’s already sugar in it, so I didn’t look at it if I took that product and added some more sugar.** No, um, if you want to go back to the reading, there’s a little bit of information on added sugars. At the very bottom. **(rereads section) Uh, I’d still say chocolate pudding.** Ok.

10: **I would say unsweetened tea.** Ok. And why did you choose unsweetened tea? **Because diet soda, lemonade, black coffee… to me, unsweetened tea would be about the lowest energy-dense beverage.** Mmhmm. So you’d go with unsweetened tea.

10: **Um, I would say black coffee.** Ok. Why would you choose black coffee? **Just from different people who drink black coffee always say that their doctors say that that was good, to drink black coffee with nothing in it.** Ok. **That’s all. I don’t know.** Ok. So when you see ‘energy-dense’ you’re thinking this is something that you should eat. **Mmhmm.** This is something that’s good. **Mmhmm.** Ok.

11: **I would say A.** Ok. **And it is because anything in a can uses preservatives, and they would be high in sodium.**

**11: Yeah, that’s the same thing [A], cause when I go to the grocery store and I’m looking at the soup cans or I’m making some other foods and I think, ‘oh God, I didn’t know they had so much sodium in it!’**

Ok, so those questions, we can go back to our scale of one to five, one being very easy and five being very difficult. How would you rate those questions in that section? **Um, it was pretty hard, I would say a four.** A four? Ok. … … So, for ‘nutrient-dense,’ that’s something that’s really high in vitamins, minerals, but not very high in calories. Is there another term that you think would be clearer? … … **Something is elevated. Well, I would say.**

**Um, the questions were, they were good but I had to think on quite a few of them. So I would give it a three.** Ok. And we still feel the ‘energy-dense’ and ‘nutrient-dense’ are terms that you’re having a hard time with? **Yeah.** So, for ‘nutrient-dense,’ that’s something that’s really high in vitamins, minerals, but not very high in calories. Is there another term that you think would be clearer? **I’m sure it is, but I can’t think of…** Don’t have a suggestion? **No.** Ok. But it’s the word ‘dense,’ not ‘nutrient’ or ‘energy,’ but it’s the ‘dense’ part that’s hard. **Mmhmm.** Ok. So like—**It’s the way I’m thinking. Yeah. You have to think a little bit harder. And maybe that’s good. But for what you’ve read and you see on TV or the news or something, um, they don’t use ‘dense,’ they use ‘energy’ or they use ‘nutrition.’** Calories? **Yeah, and so you’re just used to that. … …** So ‘nutrient-rich’ or ‘nutrient-poor?’ Are those more familiar? **Well, I guess, but in this setting, rich and poor, I don’t think people would think that.** That wouldn’t be better? Ok. Ok.

Portion sizes

Ok, so for that section, if you were—you don’t have great-grandchildren, right? No, just one grandchild?—um, if you were to, who’s somebody else you might be helping with their homework or something like that? **A granddaughter in Michigan.** Ok, so let’s think about her, not the one at Pembroke Hills, she’s too advanced! **Yes!** Ok, so let’s say that you were instructing your other granddaughter on this exercise. How would you explain what you’re asked to do there to her? **(reads first question)** So what you think we’re asking you to do in that question? **We’re talking about this glass of milk that is eight ounces. And so you’re asking me is eight ounces the right portion for the total amount of food here or not.** Ok.

1: **Um, so if you’re pouring the milk in the cereal, cause the cereal is dry, correct?** Mmhmm. **Ok, um, I think it’s the right portion.** Ok. So you’re interpreting that question as ‘in the context of all the food that’s there, is the amount of milk right to balance the cereal and the apple?’ **Yeah, that’s what I’m looking at.** Ok. Ok. Um, let’s pretend that we are just starting, the couple of sentences that start the instructions aren’t there. Can you go back up to the instructions a little bit, and start with the word ‘for.’ **Ok. (reads).** Ok. Is that still the same, still doing the exercises the same? **Yeah.** Ok, so those first two sentences could be there or not be there, it would still be the same to you? **Right.** Ok.

2: **(mumbles) B.** You’d say this is less than one portion? **I would say so.** Ok. And why’s that? What are you looking at to make that choice? **I look at it being high in nutrition.** Ok. The chicken, that’s something that you’d want to eat a larger portion of? **No. … *So are you saying that that’s the right amount?* Yes, I would say so.** So you would choose C, about right for one portion? **Right.** Ok.

2: **Um, yeah, I think this would be C too.** Ok. And when you decide your answer are you looking at the picture or are you looking at the amount, five ounces, in the question? How are you choosing? **I’m looking at the picture, and the picture makes me wonder the size of the meat, um, it looks like the right portion, and then I’m saying to myself the mashed potatoes look like one serving, and of course a glass of water wouldn’t hurt.** Ok, so you think everything together, everything’s about right? **Yeah.** And you said, you told him to ignore the parentheses of five, that was hard for you too? It would be easier if it was just the number, or just the word? **I think the word would be fine. Since you’re using words here, you stumble, I stumbled on five…** Ok, so you preferred the word of the numbers. Ok, same for the answer where it has more than one portion? Just get rid of that [numerical] one? **Right.**

**3: I would say it’s more than one portion, A.** Ok, and why would you choose A, more than one portion? **Uh, because a cup of rice is too much food at one setting. A half a cup is what I’ve been told.** And so in this case you’re looking at the amount that’s described, not just the picture? Or are you looking at the picture too? **Yeah. I’m looking at the picture too, but I’m looking at the amount.** Ok, and what you already knew? **Mmhmm.** Ok.

4: **I would say it’s C.** About right? **Yes.** Ok. And why did you choose about right? **About half a bowl.** Half of a bowl is about right for strawberries? **For a portion, yes.** Ok. How does that compare to how much strawberries you normally eat at a time? **Less.** It’s less? Ok, so you weren’t thinking about how much you normally eat but whether that’s the right portion or not. **Yeah.**

**5: I would say, um… I’m torn between more and about right. If that’s all you’re eating, with a drink, I would say it’s about right for one person.** For one person? **Mmhmm, that’s what it says—for one portion, I’m sorry.** Ok, so if there was other food on the plate, um, other things…? **Then it would be A, to me.** You think it would be more than one portion? But because that’s the only thing on the plate, you’re thinking that’s about right. **That’s what I’m thinking, mmhmm.**

**6: I don’t know about black beans because I don’t eat them.** So black beans are unfamiliar? Do you eat any kind of beans? Ok. **Less, I would think so. I don’t know (mumbles) black beans.**

6: **It looks more than one portion.** It looks like more than one portion to you? **Yeah, if it’s going to go in that little thing there.** So here, again, you’re looking at the picture and not the amount in the question? **Right.** Ok.

7: **Um, so we’re just focusing on the carrots, I would say about right for one portion.** Ok. Any… you didn’t really hesitate on that one, so… ok, and again, did you look at the picture or the amount? **Um, I looked at the picture, I looked at the sandwich that was there, and then I remembered it was uncooked, and I thought that was good for uncooked.** Ok. Ok.

8: **That’s about right.**

**8: Same thing [about right].** Ok. So, you’re looking at the picture? **Mmhmm.** But you didn’t think, the rice, you thought that was too much. **Yeah, I thought that was too much.** Ok. So sometimes you’re using what you know about portions and sometimes you’re just using the picture. **Right.** Ok. Depends on how familiar you are with the food itself? **Mmhmm.** Ok.

9: **Uh, I’m going to take a guess cause I don’t know. I would say five ounces.** Ok. And why did you choose five ounces? You said it was a guess, but what made you use that one instead of the other two? **Well, I’m not sure of the weight, but I just thought three ounces, once it was cooked, would be nothing.** Ok. **Five ounces would be enough for a good sandwich.**

**9: I would say three ounces.** Three ounces? Ok, and why would you choose that? **It looks like the right portion.** Looking at the picture it’s about right? **Yes.**

**10: I think this one.** The middle one [half cup]? **Yeah.** Ok. And why’d you choose that one? **Because rice is my favorite (?), it’s about a half cup.** Ok, so you chose the amount, not the picture? **The amount.** Ok.

10: **Same thing [half cup].** Same answer? **Mmhmm.** Ok.

11: **Ok, where does it say serving… Serving, two tablespoons? Is that what that says? Ok, two tablespoons, so it would be B.** Ok. **I would have selected B anyway.** Even without the label? **Mmhmm, just looking at the picture.** Ok.

***Interview 11***

Portion sizes

Ok, stop right there. Does that first paragraph, those instructions, do they seem clear to you? Um, do you know what you’ll be doing next based on what those instructions imply? **It was clear, um, ‘the right amounts as advised,’ I mean, I don’t really know if anybody advises us about what the right amounts are. Um, I mean you see the little plates that have… but it’s not really something that we, we don’t really buy it. We buy something we like, it’s not necessarily… I mean, there’s only two in our family, so I’ll buy a container that’ll last us for days because there’s only two of us to eat it so… so that’s, you know, I think that’s a little, what the right amounts are, I don’t think we really know.** Ok. But the instructions themselves, you think you have an idea of what you do from here? **Yeah.** Ok. Alright, go ahead.

1: **Oh, I’d say it’s probably right, the right amount.** Ok. When you answered that, did you look at the picture? **Yes.** Did you look at the text, did you look at both about equally? **I looked at the picture because I didn’t, um, I really don’t know what size my glasses at home are. So I wanted to see about what size, when I pour a glass of milk, what size I usually do. And I usually pour it all the way to the top.** Ok. When you read the word ‘portion,’ what does that mean to you? **Um, nothing really.** Ok. **‘Cause it’s just what I want.** Ok. So when you answered that question, you, you based your answer off of what? **Um, more the visual.** Ok. **Yeah.**

**2: Oh, well that’s, that’s definitely one portion.** That’s one portion? **Mmhmm.** Ok. And again with that one, did you look at the, the picture again, um, did you look at the text or what did you rely on? **Um, I looked at the picture.** Looked at the picture to answer the question. **Yeah, to see if that’s the size I usually, ‘cause I usually (mumbles) chicken, my husband eats two and I eat one. And that’s about the size.** That’s about the size that you would consume? **Yeah.** Ok. Ok.

3: **One cup? Uh, that’s more than a cup.** So you think the picture looks like more than one…? **Mmhmm.** Ok. … um, how would you choose the answer to that question? **I thought the, well, if that’s a cup, the picture looks like it’s more to me.** Ok. And a cup… so you’re judging by the picture, that one cup would be more than one portion? **Yeah.** Ok. What about if you just had the information that it was one cup of rice, if you didn’t have the picture? **Well, if they told me that I’m supposed to eat one cup of rice, I probably would measure it out and serve that amount. Um, I never really know what size to, to eat of anything, so that picture, to me, looks like a lot of rice.** Ok, so it’s helpful to have the picture there to decide? **Yeah.** Ok.

4: **I don’t really measure it, so that’s about right.** You’d say that… **That’s about three strawberries cut up. Three or four.** So, with the rice, going back to that previous question…

5: **Oh, that’s about right.** That’s about right? **Mmhmm.** Ok, and can you kind of tell us why you…? **‘Cause that’s about what I serve.** That’s about what you serve. So you’re, ok, so when you answer these questions you’re sort of thinking more about what you serve your husband and yourself. **Mmhmm, yeah.** Ok. What you would eat. **Yes.** What you would eat. And you said that’s because you haven’t, is that because you, um, don’t, haven’t been told what portion sizes are or you aren’t familiar with where to get that information or…? **Just never really seen…** Never really thought about that? **Yeah, I mean, when you buy a product, it says, you know, ‘serving size, X amount.’ But I don’t know anybody that actually measures it out, and so they, if you… What I did is I bought the right size plates so that when I put a portion on there, the bigger the plate the bigger the size, so I scaled down. That helped a lot with the, because I figured we were probably eating more than what we should.** Ok. … With the, um, pasta and the rice question, did you automatically assume that it was cooked? **Yes.** Ok. **Yes.**

**6: That’s a lot of beans. I think it’s more than one portion.** Ok. Do you eat beans? **Mmhmm.** Ok.

7: **A lot of carrots. And it’s a half a cup? Ok. Hmm, is it more than one portion? Yeah, I’d probably think it’s more than one portion.** Ok. So more than what you would serve your husband or yourself? **Yeah.**

**8: Hmm. I think it’s less than one portion.** Less than one portion? **Mmhmm.** Ok. **When we eat salmon, we eat more than that.** Ok. It’s less than what you would serve your husband or yourself. **Mmhmm. But if it’s a portion, if that’s what three ounces, I don’t weigh it, so…** So again are you looking at the picture? **Yeah. You know, to tell you the truth, I think it’s probably right.** Ok. **It’s just that there’s so much rice on the plate.** Ok, so the rice distracts from the salmon? **Yeah, it does, ‘cause it kind of takes it out of proportion.** Ok. But you, so in just looking at it again, based on if the rice was taken out, you would say that would be about right for one—**Yeah, if I stop looking at the rice, because it looks like an awful lot of rice.** Ok, but that’s based on kind of what you would serve your husband or what you’d serve yourself? **Mmhmm.** Ok. And just looking at it, eyeballing it? **Yeah.** Ok.

9: **I think the five-ounce.** Ok. **Cause it fits the bun.** And again, is that what you think you should eat or what you would eat? **Um, that’s probably what I would eat.** That’s what you would eat. If we asked you, you know, what do you think you should eat, how would you answer that question? **Probably the smaller—not eat it at all. … I probably would tell you, cause I don’t eat a lot of read, I mean we eat red meat, but not a lot. Hamburgers are one of the ones that we kind of cut out.** Ok.

10: **Well there I’d say the one cup.** Ok. **And I don’t know if that’s cooked rice.** Ok, so for there, it would probably help, do you think it would help if all the questions we specified cooked? **Yes.** Ok. **One cup of uncooked rice (mumbles) I think we do two cups, but I don’t think I’d eat a whole cup.** Ok. What do you serve yourself? Do you know? If you were to… **Just a spoonful.** Just a spoonful? **Mmhmm.**

**11: I can’t tell.** Cause it’s unclear? **Mmhmm.** Ok. The picture is unclear or the text is unclear? **It says the serving size is two tablespoons on there.** Ok, on the food label? **Mmhmm. But how do you measure what you put on the bread? I mean that’s, once you get two tablespoons and put it on the bread and that’s (?) if you dip the knife. So it’s hard to, I think that’s where the things on, when you buy stuff it’s kind of confusing because like that you would use a butter knife to spread it, but you wouldn’t necessarily know what two tablespoons are.** Ok. **I think that’s where (mumbles)** It’s confusing? **Yeah. Just because we don’t, you know, we put as much as we want on the bread. We know we’re going to use one slice of bread, so we coat it, and, and, uh, the other thing is that the picture doesn’t tell you if it’s crunchy or smooth. That’s a big difference because if it’s crunchy you have to put more on your bread.** Ok. **Smooth it kind of smooths over, put it on toast real quick, where the crunchy doesn’t work that way. And it doesn’t tell you what kind of peanut butter it is.** So that one, you’d consider that kind of a trick question? **Yeah. I think you need more before you can actually answer it, you need a little bit more information.** So the more information that you need is is this smooth or crunchy peanut butter, and what other information would you need? What visual would help there? If we’re talking about… **Um, I think it’s confusing because you use a knife to spread it with, not a tablespoon. That’s the confused part for everybody.** So, um, are you thinking that it would be better to have a visual of peanut butter on a knife than peanut butter on the bread? **Um, yeah, I think putting it on the bread you, it’s confusing. I think if you add, like, if you had peanut butter and you said ‘this is one tablespoon’ and had a big glob, then it would be, because spreading it I think is confusing because you don’t know, don’t know how much it is actually on that bread.** Ok. **I mean, you say three tablespoons, but three tablespoons and two tablespoons look the same to me.** Can you say that last part again? **The three tablespoons and the two tablespoons pictures. Now the one tablespoon, you can kind of see the bread, but the other two look pretty much the same.** Ok.

Ok, so overall for that section, say we have a scale of one to five, with one being ‘that was pretty easy, that section was pretty easy,’ to five being ‘that was really difficult.’ How would you rate that section overall? **Probably a two.** A two, ok. And why would you rate it a two? **Um, because I was confused on a couple of the questions, I had to really study and I really looked at the pictures a lot.** Ok. So, the last question was confusing, what other one would you say was…? **The rice.** The rice, the one where you couldn’t tell if it was cooked or uncooked? **Yeah.** Ok. Um, any foods in there that were unfamiliar? Any foods that you suggest should be there? **A vegetable.** A vegetable, ok. **Because when we fix a plate, we usually, asparagus, it’s, I was thinking of that with the salmon, what I cook with it. So a vegetable on the plate.** Oh, on the plates? **Mmhmm, just for, cause that’s normally what we (mumbles).** Ok. Were the pictures helpful? Ok. But would you say in some of the pictures that other foods may be distracting to you, like you said something about salmon and the rice, the rice was a bit distracting. **Yeah.** So the, if it was just the salmon on the plate, would that have made it clearer to you or, um, any feedback on that, or how that would change your answer, if it would change your answer? **Um, I think it was good to have something on the plate.** Ok. **Because then you get a comparison.** Oh, ok, I see. **But I think that the rice because it was way, you know, it was spread out, it was distracting.** Ok. That makes it trickier? **Yeah. Um, and, you know, usually, I mean, I don’t spread my rice out like that, I put a little ball or glob together and when we eat is when we break it up, so maybe that’s, but see that’s my personal…** Ok, so one last question. When you also read the instructions, you, um, you took that to mean as, you know, the rest of the questions we would be asking you that this is what you would eat at home when you’re sitting down to dinner? **Mmhmm.** Ok, what if we wanted to ask you, you know, what do you think you should eat? What do you think is a healthy amount or what you should eat? How would you phrase that? **Well, I think a lot of people would admit that they don’t eat healthy.** Ok. **And that they don’t really know portion sizes.** Ok. **So I think asking them, you know, (mumbles) people to say I really don’t want to mess with this because I don’t normally do it.** Ok. **So it’s, you’d have to actually, um, I think, getting kids in school first, like we did recycling. I mean, getting, they’re the ones that taught us how to recycle, so my boys would come home and they’d see me throw a can and they’d go get it, so you know. So I think that, teach them in the early age what a portion size is, then it would be easier than me at my age, you know.** So you think if people don’t, aren’t familiar with recommended portion sizes they’re just gonna compare to how much they usually serve themselves? **Right. Yeah, I think that’s what I was, yeah, I think that, cause it’s hard for me to you know, um, you know when I go out because different restaurants have different portion sizes, so what really is the right portion size? So, really our, we really weren’t educated on that or, told to eat our vegetables or, you know, but not necessarily how much.**

**Consumer skills**

Are you the primary shopper in your house? **Yeah. …**

**1: I would think the apple because it’s fresh.** Ok.

2: **I wouldn’t (mumbles) unless it’s fruit snacks.** Go ahead and tell us out loud what you’re thinking. **Raisins.** Raisins? **Yeah. Um, those look like candy, those look like gummy bears.** Ok. **And so, I would eat them, like, if I was going to eat candy.** Ok. **But if I wanted a boost in energy and a good, it would be the raisins that I would eat.** Ok. Have you seen fruit snacks in a grocery store or purchased them yourself before? **Mmhmm.** Ok. So you’re familiar with fruit snacks? **Mmhmm.** Ok. **I have grandkids that I take care of once a week, so we have to have a lot of those (mumbles).** Ok.

3: **If it’s lower, it would probably be the beef sirloin.** Ok. **(?) the other one looks fatty.** Ok. And so you’re judging by the appearance of the pictures?

4: **More calories, um, probably the MinuteMaid.** Ok, why did you select the MinuteMaid? **Because I saw fat-free milk.** Ok. **So I thought that that would probably be more nutritious, more better for you.** Do you, um, purchase both juice and milk yourself? **Uh, apple juice, yes, but not for me, the only thing I drink is orange juice. It’s the grandkids that drink the apple juice.** Ok.

5: **Oh, frozen.** Ok, tell us why you think the frozen. **Because it doesn’t have the sodium preservative in that one.** Ok. **In the can. When I started getting high blood pressure, that’s one of the things they told me to start watching. It kinda can get back out of control if you’re using a can that has sodium in it.** Ok. Good.

6: **The fresh potatoes.**

**7: Uh, it’s the kale. I know that.** Are you familiar with kale? **Yes.** Do you eat kale at your house? **Um, yes, what happened was I was diagnosed three years ago with breast cancer. And one of my daughter-in-laws, she made all these foods that were supposed to be (?). And I got, kale was one of the ones, I’d never had it growing up, and then I just, I started buying it after that. I do like it.** Good!

8: **I can’t see that one. (mumbles) I have trouble reading the nutritional facts because they make it so small.** So the print is small on the packages? **Mmhmm, so if they say ‘no sugar added’ on the front, you tend to, I think I tend to buy that instead, because I think that that, then I can read the other thing later, but that if I’m in a hurry and I’m grabbing stuff—**It stands out to you? **Yes.** Ok. So, when you see in the question, ‘best nutrition,’ or, I’m sorry, ‘best information,’ what, what do you think that means, ‘best information?’ **Um, more noticeable. More, kind of, it’s flagged for you. I’ve read an article that said that’s not necessarily true, but…** So you see ‘best information’ as being most accessible or easiest to find. **Right.** Ok.

9: **The blueberries. I don’t know what that, the juice, I don’t drink that stuff. (?) It would be the fresh blueberries.** Ok. Are you familiar with that brand at all? **(?)** If it was just a generic juice, would that make a difference in your answer? No, ok. You’d still pick the fresh. **Mmhmm.**

**10: Oh, the, the big one.** The ‘whole grain’ statement? **Mmhmm.** Is that kind of how you answered before, it’s eye-catching? **Yeah, it stands out. Yeah.** So if we asked, um, ‘what is most reliable,’ or ‘most trusted,’ would that change your answer on any of them, or would you stick with the same answer? **Well I think, I think I’d still stick with the answer and the reason why is because that would mean that I would have to look at every, all the breads and read that little bit of type. And where here, if I’m grabbing a loaf of bread real quick if it says ‘whole grain’ I’ll pick that one over, and the white one may be just as good, but I, it’s just harder to figure that out. And since, if, if you don’t know the brands, you don’t, you know, you’re really gonna go for what’s seen first.** Ok. **Labels are very confusing.**

For that section, let’s go back to that one to five scale. Um, one is very easy; five is very difficult. How would you rate that section? **Um, it was more difficult, I thought it was like a three.** A three? And why would you rate it that way? **Because it was, um, it made me question myself on the processes that I use for deciding, especially on the labels things. It was a no-brainer on the fresh fruits and vegetables and things like that, but on the labels, you know, (mumbles) maybe I should start reading those labels. So that’s a purpose to get people to start paying attention, I think it… It was, it’s a good section.** Ok. Does your husband ever read any of the labels or…? **Him, no.** No.

First section.

**(reads until end of second paragraph) Ok, and again, ‘energy-dense foods is kind of confusing.** Ok, why is it confusing? **I would, ‘a healthy diet is also low,’ I would just, I think of sugar. I mean…(?)** So those are terms that are unfamiliar, ‘energy-dense’ and ‘nutrient-dense?’ **Yeah. I mean I eat, I buy energy bars, but I don’t think that that, I’m buying it for that reason, I just buy them because sometimes I skip eating and they’re better for me than anything else. (?)** So you think that ‘energy’ is something positive? **Uh, yes. (reads next sentence) I didn’t know that. Hmm, that’s interesting.** So, um, going back just to that last section that you read, even though you were unfamiliar with those terms, did what followed that, did that help explain things better to you? Did you learn something from that that you could go home and tell your husband? Or how would you tell your husband about this, what you just read? **Uh, I’d tell him that… I don’t know if I would tell him anything, to tell you the truth. I’d have to, I’d want to, it questions, again, myself and what I thought was good for me. Um, ‘a healthy diet is also low in energy-dense foods.’ And I don’t really know what that means. I know the sugars and, gives you, you know, a spurt of energy, but it’s not good for you. But I don’t know what other foods would (?) for that.** Ok. **So that’s, that’s where I’m getting confused.** So when you stopped there, you’re kind of wondering, you need more information? **Yes, yes. Examples, maybe, um, what you consider an energy-dense food, besides a piece of chocolate or a chocolate bar. I mean, a little bit, is an orange an energy-dense because it’s got natural sugars in it, I don’t know, pineapple…? What do you, what else should fall into that category? I’ve never heard that category before.** Ok. When you think of the word ‘dense’ what do you think about? **Um, less.** Less? Ok, so that section was a little bit confusing. **Yeah.** Ok. **(continues reading, just next sentence) Again, ‘nutrient-dense’ is not a familiar term. I would, you know, ‘eat more fruits and vegetables are examples of nutrient-dense foods.’ Again, I don’t think I’ve ever heard that phrase before (mumbles). (continues reading from ‘plant foods’ to ‘decrease disease risk’)** Did that help explain ‘nutrient-dense’ a little bit more though? That term? **Yeah, a little bit more, but it’s still confusing, what that is.** Still confused. **Mmhmm, cause it says ‘plant foods,’ so are you talking about tomatoes, what, you know, most of our vegetables are plant foods, so why are they, you know, what does that mean…?** So, um, ‘plant foods’ is an unfamiliar term? **Right.** Ok. What would you use in place of that? **Um, you said earlier, fruits and vegetables are examples. Ok, I would just say that, cause you’re repeating yourself there, (?) just make that and just say, ‘because they provide more vitamins and minerals.’ Not ‘plant foods are nutrient-dense’ again. You already told us.** ‘Fruits and vegetables are examples because they provide many vitamins and minerals.’ **Mmhmm. To me, when I read the first paragraph I understood it, and then it repeated itself, but then it didn’t give me the fruits and vegetables again.** So, introducing ‘plant foods’ makes it more confusing? **Yeah.** Ok. **(reads ‘whole grains’ section) Ok. I think what came to mind right away when I read that was the, you know how they’re modifying the rice and wheat and… and so I thought, well, how do I what I’m eating here? If its entire…** If it is a whole grain, for sure? **Yeah.** So that could be misleading to some people? **Right. Cause I know my daughter-in-law, um, she watches her daughter’s weight a lot and she told me, one day I made, um, macaroni and cheese for her and she told me next time I needed to buy a certain pasta. And I really don’t know why it would be better for her because it was, you know, either it’s not good for you or it is good for you, so how could changing the whole grain to a…** So even if it says ‘whole grain’ that could still be misleading? **Mmhmm.** Ok. Is there a better way that we could specify that, that, you know, or what do you think we should say there so that it’s not misleading? **Um, I don’t know if you would want to change that. Because it’s very factual. You know, I think what it does is it makes people then question.** What they’re eating? **What they’re actually (?).** Oh, I see what you’re saying! Ok. **Cause, um, once, you know, once I looked at it I thought, well, you know, I maybe need to start looking at that. Not just because my daughter told me to. (reads ‘lean protein’ section) I knew that.** Ok. Does that section, you know, since ‘nutrient-dense’ was an unfamiliar term, but does that help explain it to you a little bit, or did you learn something from that section by reading that, or…? **I just didn’t know that they were classified as ‘nutrient-dense.’** Ok. So if your grandchild came home and… how old is the oldest? **Uh, the oldest is sixteen.** Ok. **Sixteen, nine, seven, three, and two.** So maybe a little younger. So let’s say the seven- or nine-year-old came home with homework and they were supposed to define ‘nutrient-dense.’ How would you explain to them what ‘nutrient-dense’ means after you’ve read through that? **Um, I’d tell her that, um, that the chicken she eats, you know, the, I don’t think she’d know the difference between the lean meats. She knows about the vegetables, you know, the peas and the dry beans, the fish, I think that would be, I’d have to show her.** Give her examples? **Yeah. Or if I was, you know, what we do is we talk about, after school you come home and what’s your healthy choice for a snack? And of course you want marshmallows, of course you wouldn’t want carrots. But I think that just, they’re learning that now in school, so it’s healthy. But before, you know, I never bought fruits because, for my boys because they were (?) I bought them because that’s what they wanted to eat.** Ok. So if they said, if she said but why are these healthy foods? **That would be difficult. Because, um, or I don’t like that, or I don’t want that, why can’t I have this?** Mmhmm, it is hard. **It is indeed. Because you can’t, you know, what do you substitute for that that she would like just as well? They get tired of eating yogurts, you know, I’ll cut up apples and stuff, but they get tired of that. They want to see what they see on TV. (reads ‘eat less energy-dense foods’ through ‘increase disease risk’)** So for you does that help explain ‘energy-dense foods’ more? **Mmhmm.** Ok. **Yeah, as you go through this, the more, and I think it’s the repeated-ness of the words, it’s helping to get more clarification on that. So I think that helps because you’re, it’s constantly being used.** Good. **(reads ‘refined grains’ through ‘too many can lead to weight gain’) Now what is ‘too many?’ Um…** So you think that needs to be defined in there, what does ‘too much’ mean, or ‘too many?’ Or are you asking that for your own knowledge? **Yeah. Well, no, I think they’d want to know how much ‘too much’ is. I think (?), you talk about, you know, the size or whatever, but is, if you eat saltine crackers, if you eat four crackers with a bowl of soup, is that too many?** So, what if it said, ‘refined grains are low in healthful nutrients and could lead to weight gain?’ Does that help take out that questioning of…? **Well, yeah, but you’d want to give an example of what…** So you’re wanting an example? **I want, yeah, because, uh, refined grains, I didn’t know white bread and saltines fell into that, I should have, but I didn’t. But I think it helps to know how many, like, one, more than one slice of bread or more than six crackers? You know, I mean, because I think people are gonna still eat it, but what is actually too much? (continues reading ‘saturated fat’ through ‘shelf-stable foods’) What is shelf-stable foods?** So is that an unfamiliar term for you? **Mmhmm.** What do you think it means? **Shelf-stable… something that doesn’t, that I can buy that I don’t have to watch the date.** Ok, yep. **Five years later I’ll find it and I can cook it.** So what kinds of foods, then, give me some examples of foods that come to mind. **Macaroni and cheese. Um, rice I think is, trying to think what I have in my cupboard, um…** So you’re thinking of foods that are in your cupboard? **Cake mix, yeah, stuff that I…** Ok. Mmhmm. **(continues reading ‘taking in less sodium’ to end of paragraph) And I know that taking in less sodium is (mumbles) pre-packaged foods, preserved, some of them are really high in sodium.** So, um, the ‘shelf-stable’ term, would you use a different term to describe what you just described to us? **Um…** You said ‘pre-packaged.’ **Yeah. I think it would be more like ‘pre-packaged,’ um, something that doesn’t, like, you know, um, I was thinking of tuna, I mean, is that…** Canned goods? **Yeah, something like that would, cause you want to, I think people would want to know what are shelf-stable foods. To see if they had them, if they would use, that’s the only thing.** Ok. **(continues reading ‘added sugars’ to end)** So, over, so the beginning of that you were unfamiliar with what ‘nutrient-dense’ and ‘energy-dense’ meant, but by the end, did that help explain those terms more? **Yes.** Ok. So you could provide examples to, maybe, your granddaughter, what energy-dense foods are and nutrient-dense foods are. **Yeah.** Ok. **And why she shouldn’t eat the cookies and drink the soda-pop.** Ok. And also the refined grains, it sounded like that was a familiar term to you, but weren’t exactly sure which foods were refined? **Well, my husband is a microbiologist, and so he’s talked about all this, cause he did research for (?) chemicals and they had a farm out there and so they did a lot of the testing back there when he worked for them. And so he used to always tell me about it and so now I’m always asking him, well how do I know if it’s been modified or not. And, you know, especially now that they’re talking about meats that they’re gonna start adding some… well how do you know, because we just now are at the point where we’re starting to understand the difference of what’s good for us to eat, the white bread or the whole grain, you know, why you shouldn’t get that white bread. But if you’re modifying it, is that the grain you’re modifying? The one that was… it’s very confusing because of the scientific, and we know why they started modifying because, you know, we had a shortage of food and (mumbles) in the country (mumbles) in other countries and that’s why we did it. But now we’re, we’re actually consuming that stuff now, so… (mumbles) they talk about the, you know, the vegetables and whether they’re organic or not. And then, what, I was in the middle of all my cancer treatment and on the news that not all organic food was actually being organic, you know, and I thought, ‘well how do you know?’ You know, so again, you know, this thing it’s very confusing to people because of that.** Mmhmm, you’re right. It absolutely is. I totally agree. I do too.

1: **(?) whole grains, now.**

**2: I don’t… energy-dense…** What would you guess out of those? Tell us why. What kind of process, how you eliminated answers. **Uh, I think it, probably the ice cream. Because of the sugar content.** Ok. So were you thinking back to the reading? **Yeah, I was trying to remember what…** Those terms meant? **Mmhmm.** I can’t tell, did you go back to the reading to look? **No. Should I?** Well, you can. That’s an option. **Is that the right answer? Is ice cream energy-dense?** Yes, that’s the correct answer. **Ok. Because it takes away. ‘Energy-dense’ is actually negative, uh…** Yes, even though it seems like—**It is. Because it’s kind of like the two words together kind of make you think, ok energy is always, seems good. But when you say ‘dense’ behind it, then I thought that’s why it’s actually (?).** Good.

3: **I’m getting confused now.** Can you tell us why you’re getting confused? **Well, again it’s the ‘nutrient’ is a positive, it’s good, but the ‘dense’ makes it a negative. So, you would, soda would be the, French fries would be too.** So, are you thinking, because you said before ‘dense’ means low, right? **Mmhmm, so ‘dense’ would be not enough nutrients in it. But it says, ‘should be recommended most often,’ so I don’t, I didn’t think they should be recommended at all. They’re ‘nutrient-dense,’ which gives you no benefit at all. Right?** Is that what you think? **That’s right, yeah.** Ok. Go back to, maybe, what you read before. **(mumbles, reading) Oh, ok.** Did that help? But you’re getting tripped up on the ‘dense?’ **Mmhmm, on the ‘dense.’ Because the question before, if they were energy-dense, but I just found out earlier that some of the energy bars that I was probably eating were really not good for me. So I guess it’s educating myself on what this is.** So is it a little bit hard to do because you’re learning as you’re reading? **Right. And then, um, like, I didn’t know that too much energy isn’t actually healthy. That high blood pressure (?) gain weight. But with your nutrient-dense… yeah, that’s very confusing. You know, putting them together like that, it’s very.** So after, so, reading what you just read, how would you go back to answer that question? **Ok, so, I would do an orange then.** Ok.

4: Tell us what you’re thinking. **Um, I’m looking at the words over here and I’m thinking (mumbles) what some of these are.** Ok. Tell us which ones—**Niacin? I don’t know what a niacin is. I know what calcium is, I know what cholesterol is. But I didn’t know you could eat cholesterol. I thought it was what you got. So I would then pick carbohydrate, because it sounds like something that’s been made up.** Cholesterol sounds like something that’s been made up? **No, cholesterol is, I thought that was the end result.** The blood tests. **Yes.** Oh, ok. Not something that is included… **Yeah, for saturated fat, sodium, and (mumbles).** Ok.

5: **It’s good for you… nutrient-dense.** Ok. Based on what you read before? **Mmhmm.**

**6: Animal-based.**

**7: (mumbling). I have no idea. (reads answers again) (mumbles) I have no idea.** Ok. **On which is the best choice of all of these. And if nutrient-dense (?).** Is it, is there just too much information provided there for you to select from? Or what is it that’s difficult to make a…? **It’s, there’s too much, um, and I think it’s a personal choice of, I mean, I think if you told me that, you didn’t tell me before which, if I had a plate of food, which one was nutrient-dense. So I didn’t know which was the lowest of all three of them. So now there’s four choices here and I have nothing to refer it back to to tell me, cause I thought up here nutrient-dense was fruits and vegetables. You’ve got meats in there. And so, how does that fall into all that? I guess that was the confusing part.** So now when you’re thinking nutrient-dense you’re mainly thinking about fruits and vegetables only? **Mmhmm.** Ok. **The only one here is the green beans, it has steamed green beans. So I guess that would be probably the best of all the choices.** Ok, that third choice? **When you start knocking out the chips, the garlic bread.** Ok. **The lowfat milk is good for you, but, you know, you’d have to go down and start checking out which one has the least of all the bad things.** Ok. So with that third choice, it was just based on that, the inclusion of that vegetable there? **Mmhmm.** And you think this question just takes too much time to answer? **Mmhmm. But it could be that if you, when you showed nutrient-dense foods, that you showed a picture or something of what a nutrient plate would look like. Maybe that was all, cause then I would have something to refer to.** Ok. **Cause here I just went through the list and said, ok, chips are bad, so forget that one; so kid of—**Process of elimination? **Mmhmm.**

**8: Blood pressure.** Ok. You arrived at that one really quickly. **Yeah.** Just something that you know?

9: **Chocolate pudding.** Ok.

10: **(mumbles) I don’t know.** What are you thinking there? **Well, I thought right away the soda.** The diet soda? **Mmhmm. But then, I don’t know the diff, which is, is worse for you, diet soda or lemonade. And I never drink black coffee or unsweetened tea.** So you’re trying to eliminate ones that you know wouldn’t be correct? **Yeah. Yeah, and I still wouldn’t know which one of those four would be energy-dense beverage.** When you think of lemonade, what do you think of? **Um, sugar.** Sugar. Ok. When you think of diet soda, what do you think of? **(mumbles) that chemical that they put in it.** Ok, what about black coffee? **Black coffee, uh, well see when I got, when I was told that I had high blood pressure they said I had to stop drinking coffee. So black coffee is not good for me, cause see the caffeine in it.** Ok. **Though it kept me going through the day, it was bad. And unsweetened tea, um, black tea…** You’re thinking of the caffeine itself as the bad ingredient? **Yeah. Yeah. I don’t know if it’s energy-dense or not. I don’t know if any of those are energy-dense.** So if you were taking this would you just skip the question, or would you try to figure out an answer? **Probably. I would probably skip it.** Ok. **It’s very, you know, cause this is the first time I think you brought in another beverage (mumbles) this whole thing, so maybe if earlier we had referred to beverages in there, (mumbles) because I didn’t see it before, it’s like, ok, which one (mumbles) and I would just be picking and choosing what I don’t like.** Ok. Ok, preference, so that by then you’d be going by preference? **Mmhmm.** Preference. Ok.

11: **Oh, it’s gotta be this, the canned tomato soup.** Ok, why did you pick that one? **Because it’s canned.** Ok. **It’s tomato. And I know all the other ones are good for me.**

Ok, so for that section, again on that scale of one to five, one that was pretty easy to five that was pretty difficult. What, how would you rate that section? And why? **Um, I, I questioned a lot, so I’d probably do it like three again because it was a lot of questions (?). And then, um, I had to go back, you know, um—**To read from the—**Yeah, I don’t have to go back to, and again as I think it’s those terms are, are new to me, and so to get me familiar with those terms I think, first of all, has to happen in the (?). Once we’re familiar with it, then I can move through it a lot quicker.** So you would need a little bit more time spent working through the terms and maybe talking with somebody to, hearing about it more or something? **It’s, you know, I went to my doctor let’s say a month and a half ago, and she’s never used any of these terms.** Mmhmm. **She’s talked to me, you know, she’s never, and so it’s, again, all new and I think that’s, that’s one of the things that we tend, as humans, don’t like is something we don’t know. And so we want to go back to what we feel comfortable with.** So again, even in the magazines where you have, you get your nutrition info, you’ve never seen anything about energy-dense or nutrient-dense? Really, really unfamiliar terms. **Yeah.** Ok. It is for a lot of people. A lot of people. **Yeah. So maybe if you can get them to start writing about it.** Writing about it or people teaching about it more? **Mmhmm.** Yeah, absolutely.

***Interview 12***

Energy sources in food:

1: **What if it’s more than one? I think it’s fat and carbohydrates (mumbles). Probably fat.** Ok. **If I was gonna choose one.** You would choose fat? But it’s between fat and carbohydrate? So what made you choose fat over carbohydrate? **What makes me choose fat?** Yeah. **Well, I think there’s fat in there.**

**2: Um, I think it’s sugar.** Ok. What made you pick sugar for that one?

3: **That’s protein.** Ok.

4: **Um, I think it’s pork chops, eggs, and cheese.** Ok.

5: **I think C, maybe.** Ok. And just kind of tell us what made you pick that one over the other options. How are you processing it? **I really wasn’t sure.** Ok. **So, carbohydrates, can you define a carbohydrate, or is that what you…?** What do you think? Can you tell us what you think a carbohydrate is? When you think of carbohydrates, what are some examples that come to mind? **I know it’s in bread, too.** Ok. **But, so bread (mumbles) choice.** So is carbohydrate an unfamiliar term, or just having a hard time thinking of foods that have carbohydrate in them? **Well, carbohydrate, you hear that all the time. I don’t pay much attention to it because I don’t have any problem with it, I don’t think. I don’t know why, not really (?).** So you’ve heard of the term, but you haven’t thought of it in terms of what foods? **I don’t see it as a problem for my health, so I don’t, I haven’t concentrated on it.** Oh, ok. Ok. So when you think of carbohydrate, then, do you think of foods that are unhealthy? **They can be, for certain situations.** Ok. Cause you said you saw it not as a problem for your health, so… **Yeah, I think it’s a problem wife’s health, so she watches her carbohydrate, you know, she looks at the bread, I know it says so many, nine grams of carbohydrates or something and she goes for that instead of a whole bunch of carbohydrate in it.** Ok. Ok, so then can you kind of tell us what you think, why you chose peanuts, bacon, and vegetable oil over the other options? **Cause I didn’t think carbohydrates were in the rest of them.** Ok. That’s fair. **Process of elimination.**

**6: I think that is correct [D].** Ok.

7: **Hmm.** What are you thinking about as you read those options? **Well, I’m trying to eliminate stuff again. You know, I guess, um, I’m gonna say A because of the bread.** Because of the toast? **Yeah.**

**8: I’d say A.** Is ‘healthy fat’ a familiar term for you? **Um, somewhat. I know saturated fat is bad.** Ok. **And so I try to avoid it.** Ok.

9: **I’d probably say salad dressing.**

**10: Hmm. Maybe it’s more healthful types of fat.** Ok. **It’s just a better choice than (?).**

So, for this section, on a scale of one to five, with one was ‘that was pretty easy,’ to five ‘that was difficult,’ how would you rate that section? **Oh, I didn’t think it was difficult, um, one to five, one being…** One being easy. **Easy.** Five being difficult. **Well I’d say it’s a one, I guess.** One? Ok. Any unfamiliar terms in there, or unfamiliar foods? **No.** No, ok. Good.

Portion sizes

1: **I’d say that’s about right.** Ok. And are you answering that based on what you would eat or what you think you should? **Probably what I should eat.** What you should consume? Ok. And then when you were looking at that did you look at both, did you look more at the text, more at the picture, or kind of looked at both to answer the question? **Well I glanced at both, but I really think I probably looked at the picture to make the decision.** Ok. You kind of hesitated when you read ‘eight.’ I don’t know, I’m curious to the word in parentheses, was that confusing to have the number and the word or…? **Um, it might be a little overkill, you know, it just made me hesitate a little. I don’t know why. But I probably wouldn’t have needed both, but…** Do you think it’s better to have the number—**the numeral ‘8’ probably is—**the number would be better? Not the word? **It’s fine with me, yeah.** Ok.

2: **Um, for me it’s probably not quite enough for supper.** Ok. So you’re looking at it for how much you would want to eat? **Yeah, I probably would, I probably would want more than that.** Ok. Ok. So how would you answer that then? **Um, I’d say it’s less than one.** Ok.

3: **Well that looks about right to me.** And again, is that what you would eat? **Probably.** What you would serve yourself? Ok. **I like rice.** Ok. **I’m not so sure it’s that good for me.**

**4: I like strawberries, too. I could probably eat more than a cup.** So you would answer that…? **Less than one.** Less than one portion. Of what you would eat, what you would like to eat? **Mmhmm, if the bowl was full I’d probably finish it.** Ok.

5: **I think it’s about right, really.** Based on what you would eat, what you would serve yourself. **Just for myself, yeah.**

**6: That doesn’t even really look like a half… I’d say it’s less than one.** Ok.

7: **About right.**

**8: Less than one portion.**

**9: I like hamburgers. Probably the eight.** Eight ounces? Based on what you would eat? **Yeah.** Ok. **I probably would.** Ok.

10: **Is the right portion. I mean, right? Or what I would eat? Would I be healthier?** How would you read it? **I’m confused there, I was looking at (mumbles) is this healthier or pretty healthy I’d do a half cup; I might eat a cup (?).** You would eat a cup, so you would serve yourself a cup? **Yeah, that’s a cup of cooked, right? Not a cup before it’s cooked? Because a cup before it’s cooked is much smaller.** So you’d like to have that information, that it’s cooked? **Yeah, I think so.** Ok. **Cause that makes a big difference in the measurement. Cause, you know, it puffs up so much.** Mmhmm. **So…** But you’re trying to decide between half a cup and one cup? **Mmhmm. So probably if it was cooked, I’d (?) a cup.** Ok. And is that how much you think you should have or how much you would have, or is it the same? **I pig out at supper. If you’re talking about supper, you know, I’d probably eat that much if it was put in front of me. I mean I try to watch it some.** Ok, um, what do you think of when you, what does the word ‘portion’ mean to you? Tell us about ‘portion,’ what does…? **It sounds like limits to me.** It sounds like limits? **(?) being limited. Um, again I’m kind of thinking of supper more than anything.** If we used the word ‘serving,’ would that communicate anything different than the word ‘portion?’ **Probably. It would seem more liberal.** ‘Serving’ could be bigger and ‘portion’ is more restricted? **Yeah, exactly.** Ok.

Can you go back up to those instructions up there? **Ok.** Read through those again and tell us what you think we’re asking you to do. **(reads) Right portion size…** Do you think we’re asking you what you should eat or what you would eat? Or neither one? Or something else? **It says ‘what you think is the right portion size,’ so if you pay attention to that sentence, the end of that sentence, it’s not necessarily what you eat, it’s what you think is the right one. So maybe I didn’t (?) that clear enough.** Is that confusing, how those are structured, or how those instructions are presented? Is there a better way we could state that? **Hmm…** Cause you started off with the first question with what you think you should drink with the milk, and then you moved on to what you would eat. **Hmm, well, I have a problem drinking enough fluids, so it might be biased. I mean I know I should have more. I’ve been told especially because (?) and I don’t actually drink a lot. It drives my wife crazy. I can eat a whole meal and hardly take a drink. She’s saying what in the world is wrong with this guy, you know. It’s like I’m trying to consciously drink more, but it’s, it’s an effort.** Yeah. **So, I (mumbles).** So, it’s just kind of automatic to look at these and think, ‘how does this compare to what I eat?’ **Yeah, I guess that’s probably… yeah, cause I’ve looked at that and I’m saying the ‘right’ portion cause that’s probably, although I should drink the whole glass, (?) eight ounces is a glass, it’s a little deceptive because if that’s, how big is that glass? You know, is it a thirty-two ounce glass? I can’t really tell from the picture.** So the picture isn’t really helpful in this one, do you think? **Well you might be able, since that’s eight ounces, it looks like half of a, half of a sixteen-ounce glass is what I was gonna say, but it might be milk in an eight-ounce glass…** So when you looked at that, did you base your answer on ‘eight ounces’ or the picture? **Well, my mind picked up the ‘eight ounces’ that I read, but then on to the glass and I think it looks smaller. I don’t know if I’m being concurrent here or not.** This is good, this is good. So, if, if we, if we were trying to ask you what you think you should eat, like as far as what would be the correct portion, how could we phrase that, do you think, to make it more clear? So that you’re not answering ‘well, this is what I would eat,’ you know, ‘this is what I would serve myself.’ **That’s what you want, what I would serve myself?** No, actually we want to know if people can identify whether the portion is a recommended portion size or, um, if it’s too much or too little, compared to the recommendation. So it seems to us the instructions are unclear. Or, are people just not familiar with what recommended portion sizes are and so they just think about how it compares to what they eat? **Well that could be me, it could be relative to every person if you’re not really conscious of, of what that would be, what the correct portion is.** Ok. So it might not be the instructions itself, it’s just how people interpret, or what people know, prior knowledge, maybe? **I think so, or lack of what a portion is.** Ok. **I really haven’t had too much trouble with my weight, so (mumbles) I could eat a lot, (mumbles).** So do you think most people would answer these questions based on what they would serve themselves? **Probably so.** Ok. But it sounds like if we use the word ‘serving’ they would definitely think about how much I portion, how much I put on my plate. **Yeah.** But ‘portion’ communicates I have a more restricted amount. **Yeah. Unless you’re really into nutrition, you know, if you’re reading nutrition magazines and you know how much… and I’m (mumbles) I don’t really.** Ok.

Um, so for that section, let’s go back to that scale of one to five, one pretty easy to five that was pretty difficult. How would you rate that section? **I don’t know if I did that well on it, but, I’m not sure if I did what you were wanting…** You’re doing perfectly. This is exactly what… this is what we need, so it’s, yeah, we’re just, we’re trying to figure out what people are thinking when they go through this section, so based on, before I asked you all these questions, how would you have rated that section? **Oh, I didn’t, I didn’t think it was difficult.** Ok. **Um…** Were the foods pretty familiar to you, any unfamiliar foods that we asked about? **Oh, yeah. The foods were fine.** Ok. And would you say in terms of the information in it, did you look at the, um, amounts in the question or the pictures? What were you using to help you answer or decide on your answer? **Well, both, I guess the picture, because I’m kind of a visual person, probably had a bigger impact on me personally.** Really need the picture there? **Yeah.** Ok.

Food labels

Who’s the primary shopper in your home? **I like to shop, my wife hates it. It’s weird.** For groceries? **Oh, yeah, I like to read the labels for them.** Do you read food labels? **Mmhmm.**

**1: Serving size is a cup, you’re gonna make me think now. Well, per serving is, there’s two servings… is two-fifty, so it’s five hundred.** Ok.

2: **There’s four hundred and seventy in a serving, so (mumbles) you could only have one.**

**3: Grams total fat… (mumbles)…** So what are you thinking as you’re…? **I’m trying to do the math.** So what is, what math are you trying to do? **Twelve from sixty-five, trying to get the right number here. Can I use a pencil?** Um, sure. **The end of the day… ok, so we’ve got twelve, three… is forty-five, got more than that, fifty-nine, (continues mumbling)… sorry this is taking so long. … fifty-three.**

**4: Sixty-two.**

**5: Probably not. Zero.** Ok.

6: **Oh, wait… (mumbles)… two cups.**

**7: Oh, total fat. I’m looking at saturated.** So tell us what you did as you were thinking through that answer. **Well, I was looking, I wasn’t paying attention to the total fat and saturated fat. I had to—**Had to go back and read it? **Have to pay attention to what you’re asking for. Sorry, it’s the end of the day.**

**8: Protein. Two cups… five… Ten.** Ten? Ok.

9: Another one of those math questions. It’s ok, just tell us what you’re thinking about as you process through it. **Ok. I really need to read the thing a couple times. (reads question again). Ok, so limited to forty-five. And one serving is thirty. Well, fourteen grams is the closest, I guess.**

**10: Total fat’s twelve. Limiting to sixty. (mumbles)** What are you thinking about as you…? **Well I’m trying to do the math.** Ok. Can you kind of tell us what the math you’re doing is? **Trying to figure out how to divide that. I’m getting fifteen percent.** Ok. **That was difficult.** What is the equation that you’re roughly thinking that you need to do to answer that question? **Well you gotta put sixty over twelve… sixty… it’s been so long since I’ve done it.** That’s ok. You’re thinking right along the right lines. It’s just difficult math, yeah. **Off the top of my head it isn’t, (?).** So would it help to have a piece of paper? Would you want a piece of paper? **This is embarrassing.** Don’t be embarrassed. Very few people, er, most people have trouble with that question. Yeah. You’re actually processing the right equation there, so, yeah, so you’re along the right track.

Um, so for that section, let’s go to that scale of one to five. How would you rate that one, one easy, five difficult. **Ah, at least a three.** Ok. And is that because of the math and having to go back and forth from the food label. **Yeah. Not user-friendly, I guess.** Ok. Not user-friendly, having to flip back and forth? **Flip back and forth.** Ok. **It would be easier for me if I could see it without, and that’s just because I’m not as used to these things [iPad] as any other person.** Ok. Sure, sure.

Consumer skills

1: **I think they’re equal in nutrition. (mumbles).** Ok. **(mumbles)** Why did you decide apple over…? **It’s fresh and not processed.** Ok.

2: **Raisins.** Ok. Why did you choose the raisins? **Well it’s because it’s pure fruit. Nothing is added to it.**

**3: I would say the beef sirloin.** Is that because you know about beef? **Yeah.** Or did you, did you look at the picture at all or mostly from your experience? **It was experience, and it’s the most expensive of the two.** Ok. **Because you can see from the picture, there’s a lot less fat in it.** Ok.

4: **More calories, it would have to be the MinuteMaid. (reads question again) That’s a good question.** What are you thinking about? **I think the milk probably has more calories in it.** Ok.

5: **I think the frozen beans are probably lowest in sodium.**

**6: Fresh potatoes**

**7: Should be the kale.** Are you familiar with kale? **I don’t eat much of it, but I know there’s not a whole lot of food value in lettuce.** Ok. **In that one.** The iceberg? **Yeah.** Ok.

8: **Um, the nutrition panel.** Ok. And when you see ‘best information,’ … in the question where it says ‘best information,’ what does that mean to you? **Well I think that’s more reliable.** Ok, so reliability? **The Nutrition Facts and the front of the box.** Ok. **I think it’s got some legitimacy to it or isn’t advertising, maybe, as much.** Ok. **(?), but that could be a perception.** Ok.

9: **Well I think the blueberries do.**

**10: I think the ingredients list.** Ok. What made you choose that one? **Again, I just think it’s more reliable than…** Ok. Do you ever look at the ingredients list? **Oh, yeah.** Mmhmm.

So I noticed in this section you didn’t choose C for any of them. Um, did you…? **Yeah, I don’t think they’re equal. I don’t think any of them were equal.** So it’s because in all cases, you didn’t think any of them were equal? **Yeah, I just eliminated it right off the bat.** Ok. Ok. **To me it’s a wasted question.** A wasted answer? **A wasted answer, yeah.** Ok. So for that section, on that scale of one to five, how would you rate that? You moved through it pretty quickly. **Yeah, I (mumbles).** You did? **This is familiar (mumbles).** You didn’t have to do any math. **Yeah, no math.** So pretty easy for that one? Pretty easy for that section? **Yeah.** Ok.
